# Steric Effects of *N*-Alkyl Group on the Base-Induced Nitrogen to Carbon Rearrangement of Orthogonally Protected *N*-Alkyl Arylsulphonamides

**DOI:** 10.3390/molecules30081823

**Published:** 2025-04-18

**Authors:** Amie Saidykhan, Jenessa Ebert, Nathan W. Fenwick, William H. C. Martin, Richard D. Bowen

**Affiliations:** School of Chemistry and Biosciences, Faculty of Life Sciences, University of Bradford, Bradford BD7 1DP, UK; a.saidykhan1@bradford.ac.uk (A.S.); j.ebert@bradford.ac.uk (J.E.); n.fenwick@bradford.ac.uk (N.W.F.); w.martin@bradford.ac.uk (W.H.C.M.)

**Keywords:** sulphonamides, rearrangement, ortho metalation, steric effects, saccharins

## Abstract

The rearrangement of a total of 56 members of 22 series of orthogonally protected *N*-alkyl arylsulphonamides of general structure 4-XC_6_H_4_SO_2_NR^1^CO_2_R^2^ [X = H, CH_3_, F, Cl, Br, CH_3_O, CN, CF_3_ or C(CH_3_)_3_; R^1^ = CH_3_, CH_2_CH_3_, CH_2_CH_2_CH_3_, CH(CH_3_)_2_ or CH_2_CH(CH_3_)_2_; R^2^ = CH_3_, C_2_H_5_ or C(CH_3_)_3_] when treated with lithium di-isopropylamide in tetrahydrofuran at −78 °C has been studied. The competition between directed ortho metalated rearrangement, to form 4-X-2-(R^2^O_2_C)C_6_H_3_SO_2_NHR^1^ and the production of a substituted saccharin, is strongly influenced by the size of R^1^ and R^2^, especially in the series with X = CH_3_. When R^1^ = CH_3_ or to a lesser degree, C_2_H_5_, formation of the saccharin competes to a significant extent, especially when the migrating group is CO_2_CH_3_ or CO_2_C_2_H_5_. In contrast, when R^1^ is a larger alkyl group, particularly if it is branched at either the α- or β-carbon atom [CH(CH_3_)_2_ or CH_2_CH(CH_3_)_2_], the increased steric hindrance essentially prevents cyclisation, thus facilitating rearrangement to 4-X-2-(R^2^O_2_C)C_6_H_3_SO_2_NHR^1^ in high yield. The size of the migrating CO_2_R^2^ group also exerts an effect on the competition between the reactions: when R^2^ = C(CH_3_)_3_, clean rearrangement is possible even when R^1^ = CH_3_ in each series of X. These results have implications for further elaboration and rearrangement of 4-X-2-(R^2^O_2_C)C_6_H_3_SO_2_NHR^1^ in order to prepare substituted saccharins containing a 6-CO_2_R^3^ group.

## 1. Introduction

Aryl sulphonamides are a well-known class of compounds that have been applied in a variety of scientific endeavours for over a century. Prior to the advent of spectroscopic methods, the highly crystalline nature of several series of aryl sulphonamides was exploited to characterise amines by comparing the melting points of these derivatives with those of authentic samples [1]. Early synthetic applications of aryl sulphonamides include the preparation of unsymmetrical dialkyamines and trialkylamines by treatment of the derived anion with a reactive alkyl halide, thus obviating the undesirable di-alkylation that occurs when the parent amine is treated with the same alkylating agent [2,3] and facilitating successful efforts to prepare chiral tetralkylammonium salts [4,5]. A more general function of sulphonamides during the 20th and 21st centuries is as protecting groups, especially on nitrogen, as has been summarised in books reviewing their use [6,7]. Very recent efforts to design [8] and utilise [9,10] more selective protecting groups, as exemplified by 2,4,6-tris(trifluoromethyl)benzenesulphonamides, underline the contemporary significance of these species.

Exploitation of the biological activity of sulphonamides began with the discovery in the 1930s that sulphanilamide, which had first been prepared in 1908 [11], was the active form of the prodrug, Prontosil, which was effective as an antibiotic [12]. Sulphonamides and their derivatives are currently being applied in a wide range of pharmaceutical, medicinal and industrial contexts [13,14,15,16,17,18,19,20,21,22,23,24,25,26,27,28,29,30,31].

**Scheme 1:**
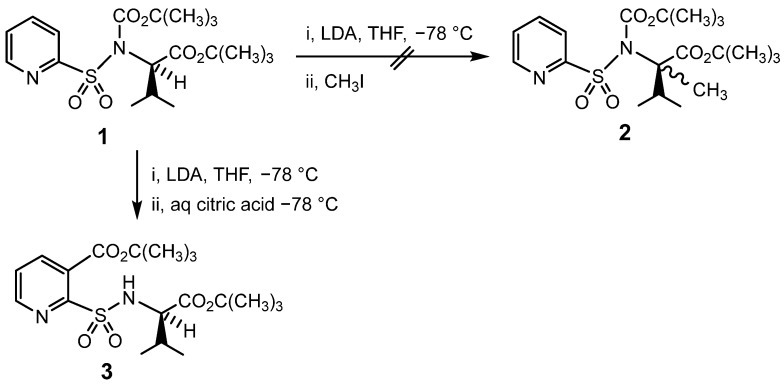
Intended and observed behaviour of orthogonally protected sulphonamide **1** derived from valine.

Many viable synthetic sequences involve the correct assumption that sulphonamides are generally inert to most basic reagents designed to modify functional groups elsewhere in the substrate. Thus, an attempt was made in this laboratory to elaborate the orthogonally protected pyridine-2-sulphonamide, **1**, by deprotonating the stereogenic centre derived from valine with lithium di-isopropylamide (LDA) in tetrahydrofuran (THF) at −78 °C and methylating the anion with methyl iodide to produce **2**, Scheme 1.

**Scheme 2:**
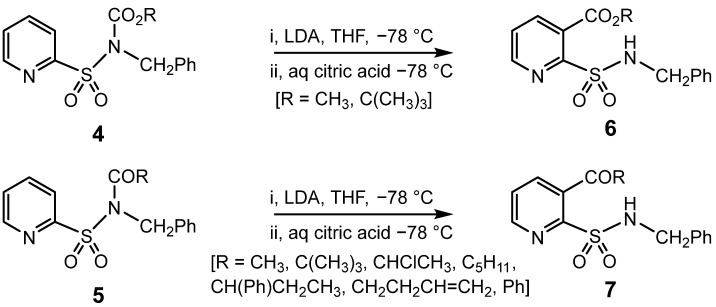
Rearrangement of ArSO_2_N(CH_2_Ph)CO_2_R and ArSO_2_N(CH_2_Ph)COR.

However, when the deprotonation was monitored by thin layer chromatography (TLC), it was found that a new product was formed essentially quantitatively before any methyl iodide had been added. This unexpected product was isolated after quenching with aqueous citric acid solution and characterised as **3**, an isomer of **1** formed by directed ortho metalation (DOM) [32], followed by eventual migration of the CO_2_C(CH_3_)_3_ group originally attached to nitrogen to the adjacent “ortho” (3) position. Further investigation revealed that this intramolecular rearrangement could be induced in good yield (75–95%) for numerous CO_2_R, **4**, and COR, **5**, groups attached to nitrogen to give **6** and **7**, respectively; Scheme 2 [33].

**Scheme 3:**
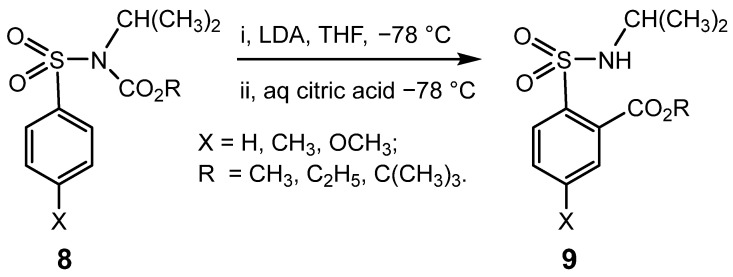
Rearrangement of orthogonally protected sulphonamides of general structure 4-XC_6_H_4_SO_2_N[CH(CH_3_)_2_]CO_2_R [39].

Intermolecular examples of DOM are known to occur for sulphonamides and related compounds, but typically under more forcing conditions with stronger bases, such as butyl lithium (BuLi) [32,34,35,36,37,38]. This intramolecular rearrangement actuated by the unusually mild base LDA was subsequently found to occur for carbocyclic sulphonamide derivatives, **8**, with an *N*-CH(CH_3_)_2_ group and a representative 4-substituent [X = H, CH_3_, and CH_3_O] to give **9**; Scheme 3. In contrast, the corresponding 4-nitrobenzenesulphonamide derivatives (X = NO_2_) did not undergo the rearrangement, thus revealing that this protecting group is compatible with modifications induced by LDA elsewhere in the molecule [39].

**Scheme 4:**
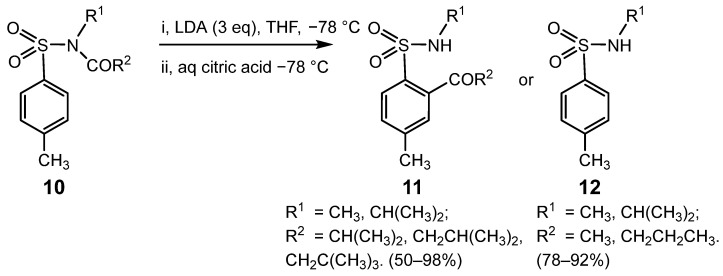
Competing rearrangement and cleavage of orthogonally protected sulphonamides of general structure 4-CH_3_C_6_H_4_SO_2_NR^1^CO_2_R^2^.

Rearrangement of **10** to **11** was feasible even if the migrating group contained an acidic hydrogen atom, provided excess LDA was used and the alkyl group was branched at the β- or γ-position to provide sufficient steric hindrance to prevent deprotonation at the α-position or nucleophilic attack at the carbonyl group from pre-empting DOM. Thus, in the 4-CH_3_C_6_H_4_SO_2_NR^1^COR^2^ series, COCH(CH_3_)_2_, COCH_2_CH(CH_3_)_2_ and COCH_2_C(CH_3_)_3_ groups migrated in fair to excellent yield (50–98%) when R^1^ = CH_3_ or CH(CH_3_)_2_; in contrast, the analogous derivatives containing COCH_3_ and COCH_2_CH_2_CH_3_ groups underwent predominant cleavage or decomposition; Scheme 4 [40]. The significance of these successful rearrangements when R^2^ contained acidic α-protons but also had branching at the β- or γ-carbon was further highlighted when attempts to replicate the process with BuLi substituted for LDA gave extensive cleavage or decomposition.

**Scheme 5:**
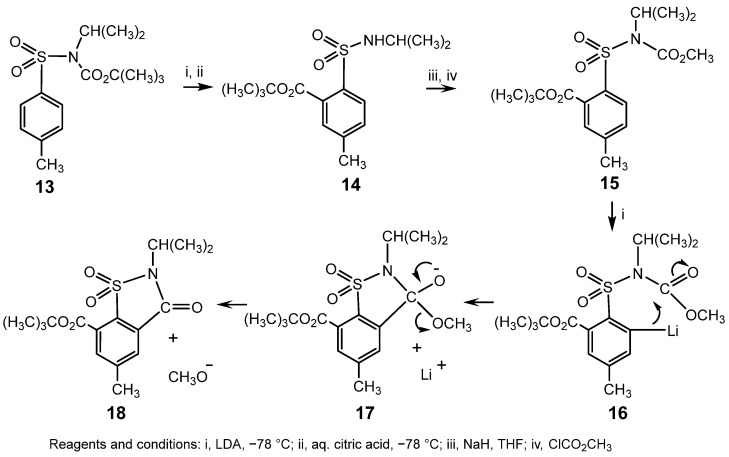
Consecutive rearrangement and cyclisation of orthogonally protected sulphonamide 4-CH_3_C_6_H_4_SO_2_N[CH(CH_3_)_2_]CO_2_C(CH_3_)_3_.

When the product of the first rearrangement was carboalkoxylated and treated with LDA, migration of a second CO_2_R^2^ group occurred to the other ortho position with eventual formation of a substituted saccharin, thus underlining the synthetic potential of this novel process [40]. The formation of the same product, regardless of the order in which the CO_2_R^1^ and CO_2_R^2^ groups migrate, has implications for the mechanism of this ‘double’ rearrangement. When the larger CO_2_C(CH_3_)_3_ group migrates first, cyclisation of the second organolithium species, **16**, gives an intermediate, **17**, which may directly expel the reasonable leaving group, CH_3_O^−^, to give the saccharin, **18**; Scheme 5.

**Scheme 6:**
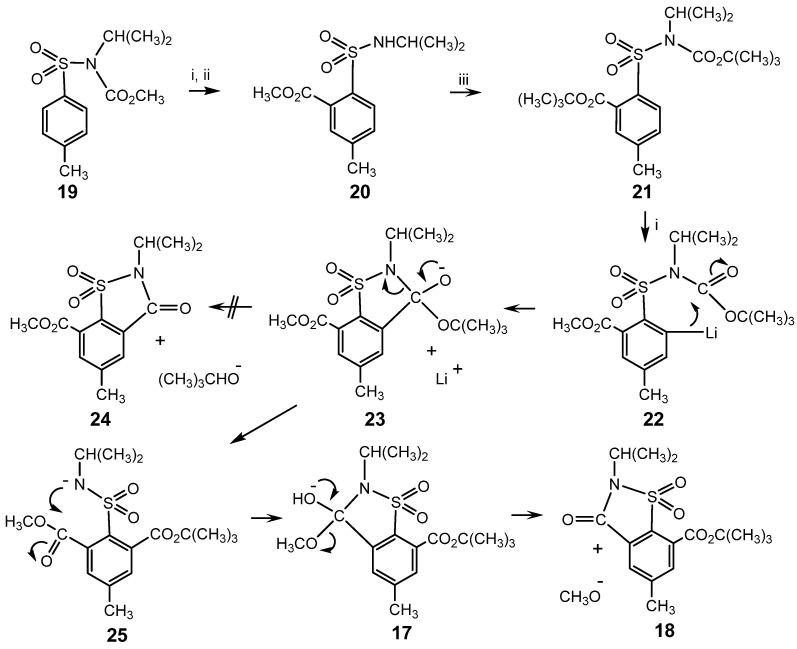
Consecutive rearrangement and cyclisation of orthogonally protected sulphonamide 4-CH_3_C_6_H_4_SO_2_N[CH(CH_3_)_2_]CO_2_CH_3_. Reagents and conditions: i, LDA, −78 °C; ii, aq. citric acid, −78 °C; iii, [(CH_3_)_3_COCO]_2_O, DMAP.

When the smaller CO_2_CH_3_ group migrates first, an analogous mechanism would yield a saccharin, **24**, containing a CO_2_CH_3_ substituent by elimination of the poor leaving group, (CH_3_)_3_CO^−^, from the anion, **23**, formed from the second organolithium species, **22**. This process is pre-empted by formation of **18**, possibly by ring opening of **23** to **25**, followed by cyclisation to **17** and elimination of CH_3_O^−^ to give **18**; Scheme 6. Alternatively, **25** might undergo protonation during the workup, to form a species with two ortho CO_2_R substituents, which would then eliminate the smaller ROH to form **18**.

When a few representative homologues and analogues of **19** containing an *N*-methyl or *N*-ethyl group were rearranged, unexpected by-products were sometimes found, thus limiting the synthetic route to saccharins corresponding to **18**. Consequently, this systematic survey of the influence of the size and structure of the *N*-alkyl group on the rearrangement of 22 series of 4-XC_6_H_4_SO_2_NR^1^CO_2_R^2^ derivatives was initiated in order to delineate the scope of the first step in the route illustrated in Scheme 5 and Scheme 6. Another objective was to investigate the influence, if any, of a variety of substituents, X, in the 4-position of the aryl group.

## 2. Results

**Scheme 7:**
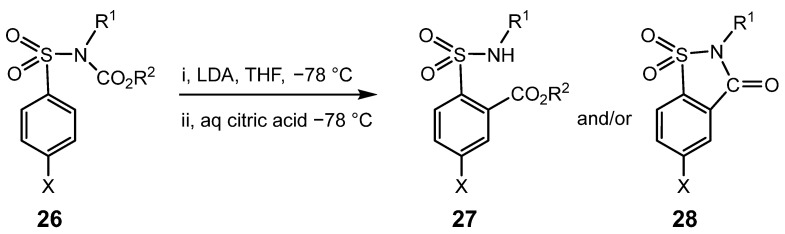
Competing rearrangement and cyclisation of orthogonally protected sulphonamides of general structure 4-XC_6_H_4_SO_2_NR^1^CO_2_R^2^.

Since less hindered members of the ArSO_2_NR^1^COR^2^ series tend to undergo cleavage of the N-C bond to give after workup the parent sulphonamide, ArSO_2_NHR^1^, [40], treatment of the analogous ArSO_2_NR^1^CO_2_R^2^, **26**, with LDA at −78 °C might have been expected to give similar undesirable by-products. However, when the least hindered member of the series, 4-CH_3_SO_2_N(CH_3_)CO_2_CH_3_, was investigated, ^1^H NMR analysis of the crude product revealed approximately equal proportions of **27** (X = R^1^ = R^2^ = CH_3_, Scheme 7), formed by the desired rearrangement, and another 1,2,4-trisubstituted benzene, which gave no signal corresponding to either a methyl ester or an N-H. This product was isolated and characterised as the *N*-alkyl saccharin, **28** (X = R^1^ = CH_3_). Similarly, when the higher homologue with R^1^ = C_2_H_5_ and R^2^ = CH_3_ was studied, a smaller proportion (~20%) of the *N*-ethyl saccharin, **28**, X = CH_3_, R^1^ = C_2_H_5_, was obtained, together with the desired major product, **27**, R^1^ = C_2_H_5_, X = R^2^ = CH_3_.

The unexpectedly facile formation of a saccharin in these cases when R^1^ and R^2^ are both small may appear at first sight to be advantageous. However, further derivatisation of **28**, to permit the second rearrangement and formation of *N*-alkyl saccharins with a 6-carboalkoxy substituent as shown in Scheme 5 and Scheme 6 would be difficult.

**Scheme 8:**
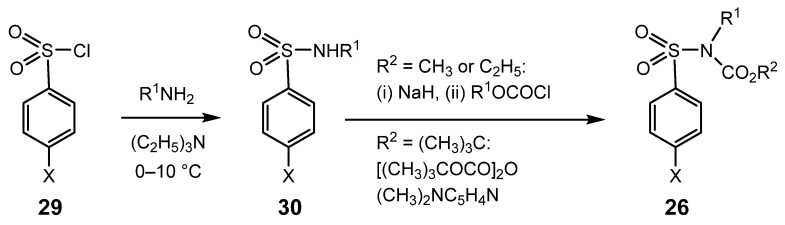
Synthetic routes to orthogonally protected sulphonamides of general structure 4-XC_6_H_4_SO_2_NR^1^CO_2_R^2^.

In order to establish which *N*-alkyl groups are compatible with clean rearrangement of **26** to **27**, without formation of **28**, 22 series of 4-XC_6_H_4_SO_2_NR^1^CO_2_R^2^ were prepared from the corresponding sulphonyl chlorides, **29**, via the requisite XC_6_H_4_SO_2_NHR^1^ parent compounds, **30**, by the synthetic routes summarised in Scheme 8. Each of these 56 derivatives was then treated with LDA under identical conditions (2 equivalents with a reaction time of 2 min before quenching with aqueous citric acid solution).

Table 1, Table 2 and Table 3 summarise the competition between intramolecular rearrangement and cyclisation (to form **27** and **28**, respectively, Scheme 7). Initial work focused on five series with X = 4-CH_3_, Table 1, so as to investigate the influence of a wide range of *N*-alkyl groups. Once the trends in these series had been established, a less extensive set of substrates with X = H was studied, in which the *N*-alkyl group was chosen to give the greatest or smallest proportion of cyclisation, Table 2. Finally, a further 14 series with a wide range of 4-substituents [X = F, Cl, Br, CH_3_O, CF_3_, CN or C(CH_3_)_3_] were also investigated to establish that the trends found in the series with X = CH_3_ and H were general and to ascertain whether any specific X suppressed or favoured saccharin formation, Table 3.

## 3. Discussion

**Scheme 9:**
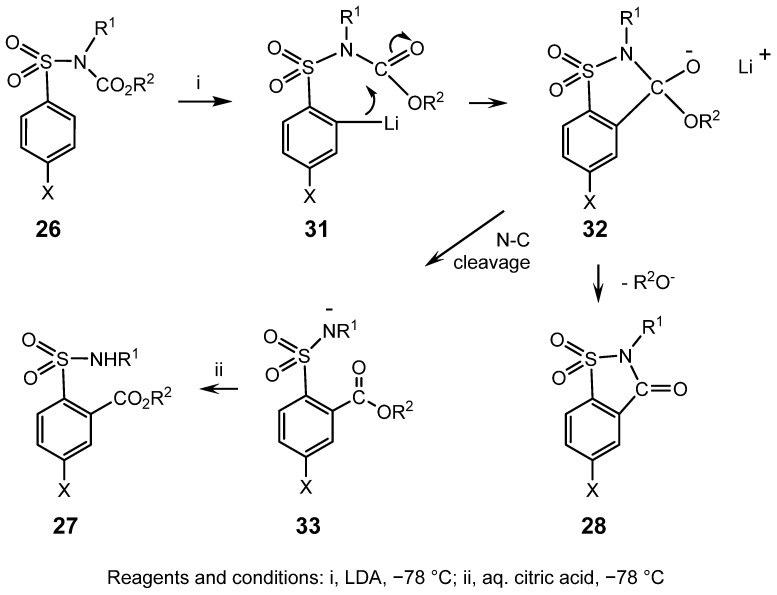
Mechanism for rearrangement and cyclisation of orthogonally protected sulphonamides of general structure 4-XC_6_H_4_SO_2_NR^1^CO_2_R^2^.

The data in Table 1, Table 2 and Table 3 reveal that the size and structure of the *N*-alkyl (R^1^) and the carboalkoxy group (CO_2_R^2^) each exert an influence on the competition between rearrangement and cyclisation, as does the nature of a substituent (X) in the 4-position of the aromatic ring. These results can be interpreted by considering the effect of R^1^, R^2^, and X on the competing reactions of the intermediate anion, **32**, formed by cyclisation of the organolithium species, **31**; Scheme 9.

The following trends are evident from Table 1.

Firstly, when R^1^ = CH_3_, the undesirable cyclisation occurs to a considerable extent when the migrating group is either CO_2_CH_3_ or CO_2_C_2_H_5_ (ratio of rearrangement to cyclisation of 1:1.1 or 2:1, respectively). The corresponding ratios for the next higher homologues with R^1^ = C_2_H_5_ are significantly greater (4:1 and 19:1, respectively). Extension of the length of the *N*-alkyl group to CH_3_CH_2_CH_2_ continues this effect, but cyclisation still competes to a minor extent when R^2^ = CH_3_ (ratio of 20:1), though less so when R^2^ = C_2_H_5_ (ratio of 50:1).

Secondly, the size of R^2^ in the migrating CO_2_R^2^ group also influences the competition. This effect is evident when R^1^ = CH_3_, for which the ratio of rearrangement to cyclisation increases from 1:1.1 when R^2^ = CH_3_ to 2:1 when R^2^ = C_2_H_5_. A stronger effect is evident in the series with R^2^ = C_2_H_5_, for which the ratio is 4:1 or 19:1, respectively, when R^2^ = CH_3_ or C_2_H_5_, presumably because, in the latter case, the combined effect of steric hindrance induced by two C_2_H_5_ groups almost suppresses cyclisation. Furthermore, branching of R^2^ has such a profound effect that cyclisation is effectively pre-empted, even when R^1^ = CH_3_, in the series with R^2^ = C(CH_3_)_3_. This trend is logically interpreted on the basis of the mechanism in Scheme 9: elimination of CH_3_O^−^, and to a lesser degree, C_2_H_5_O^−^_,_ from the anion **32** is easier than expulsion of the much poorer leaving group, (CH_3_)_3_CO^−^. This ‘electronic’ effect should reinforce the steric influence of the bulkier R^2^ group, which would be expected to favour ring opening to **33** by C-N cleavage, rather than loss of R^2^O^−^. This interpretation concurs with the explanation summarised in Scheme 6. One immediate and highly significant implication of this finding is that it would be advisable to migrate the CO_2_C(CH_3_)_3_ group in the first rearrangement, followed by either CO_2_CH_3_ or CO_2_C_2_H_5_ in the second rearrangement, if it is desired to prepare an *N*-methyl or *N*-ethyl saccharin corresponding to **18**, Scheme 5.

Thirdly, branching at the α-carbon atom in the R^1^ group also effectively suppresses cyclisation (as was previously found in the formation of one example of **18** with R^1^ = CH(CH_3_)_2_**,** in which the first of two consecutive reactions with LDA occurred without detectable cyclisation, but in which formation of a saccharin took place when the second derivative was treated with LDA [40]). In the series with R^1^ = CH(CH_3_)_2_, the ratio of rearrangement to cleavage is at least 50:1, even for the smallest migrating group, CO_2_CH_3_. Any cyclisation in the higher homologues with R^2^ = C_2_H_5_ or C(CH_3_)_3_ occurs to such a negligible extent that no sign of **28** could be detected in the ^1^H NMR spectra of the product, which appeared to be entirely **27** formed in isolated yields in the range 85–99%.

Fourthly, branching at the more distant β-carbon atom appears to be approximately equally effective in favouring rearrangement at the expense of cyclisation. Even when R^2^ = CH_3_, for which competition by the undesirable cyclisation does occur when R^1^ = CH_3_ or C_2_H_5_ (and, to a lesser extent, when R^1^ = *n*-C_3_H_7_), rearrangement occurs with very high selectivity in a ratio of 50:1 and an isolated yield of 90%.

These trends persist in the behaviour of the three series with X = H, Table 2, though the tendency to form a saccharin is reduced compared to that found for the analogous member of the corresponding series with X = CH_3_. Thus, when R^1^ = R^2^ = CH_3_, the ratio of **27**:**28** increases from 1.1:1 for X = CH_3_ to 9:1 for X = H. This result indicates that an electron-donating group in the aromatic ring favours cyclisation to **28**, perhaps because it destabilises the anion, **33**, formed by C-N cleavage of **32**.

Similarly, the strong influence of the branched R^1^ groups, CH(CH_3_)_2_ and CH_2_CH(CH_3_)_2_ in effectively suppressing cyclisation when 4-CH_3_C_6_H_4_SO_2_NR^1^CO_2_R^2^ is treated with LDA, regardless of the nature of the migrating CO_2_R^2^ group, persists in the behaviour of the lower homologues, C_6_H_5_SO_2_NR^1^CO_2_R^2^. These observations, though hardly surprising, are important in establishing the generality of the influence of these branched R^1^ groups.

Further confirmation of these trends is found in the behaviour of the 14 series in which a variety of electron-withdrawing (X = F, Cl, Br, CF_3_ and CN) or electron-donating substituents (X = CH_3_O or (CH_3_)_3_C) are present in the 4-position, Table 3. In every case, the ratio of **27** to **28** increases sharply on progressing from R^1^ = CH_3_ to CH(CH_3_)_2_. Indeed, in most cases with R^1^ = CH(CH_3_)_2_, the proportion of saccharin formed was so small that it was difficult or impossible to detect in the standard ^1^H NMR spectrum. When these orthogonally protected sulphonamides were treated with LDA, essentially pure **27** was obtained in 80–95% isolated yield. Consequently, if it is desired to prepare **27** for further elaboration (for example, to synthesise substituted saccharins of general structure **18**), it is desirable to select R^1^ = CH(CH_3_)_2_.

Another interesting trend is found in the results summarised in Table 3 in cases with R^1^ = R^2^ = CH_3_, for which formation of **28** is favoured when X = CH_3_ (as previously discussed). In each of the examples in Table 3, a significant, but smaller, proportion of **28** is formed. Moreover, in the halogeno series, the tendency to produce **27** declines as the electronegativity of the halogen increases (ratios of **27**:**28** = 17:1, 14:1 and 9:1, respectively, for X = F, Cl, and Br). This trend also is intelligible in terms of the mechanism suggested in Scheme 9. A more electron-withdrawing substituent (F, rather than Cl or Br) would be expected to stabilise the anion, **33**, formed by C-N cleavage, thus favouring formation of **27**. Similar effects are found with two other electron-withdrawing groups, i.e., when X = CN or CF_3_, which also favour rearrangement over cyclisation.

The case when X = CH_3_O, which is electron-withdrawing by the inductive effect but electron-donating by the mesomeric effect, is particularly interesting. Although the proportion of **27** is smaller than when X = CH_3_, it is greater than that for examples with any of the other substituents in Table 3. It is possible that any destabilising effect on the anion **33** caused by electron donation by the mesomeric effect is offset by a combination of the (weak) inductive effect and the possibility of “push–pull” interaction of the methoxy group with the S=O bonds in the sulphonamide moiety.

When X = C(CH_3_)_3_, cyclisation competes to some extent with rearrangement, as might have been expected because of the slight electron-donating effect of the large alkyl group. However, the effect is much less pronounced than when X = CH_3_. It appears, therefore, that formation of a saccharin is peculiarly favoured in the tosyl series.

Finally, attempts to explore other series with the even bulkier R^1^ group C(CH_3_)_3_ proved to be impracticable. Condensation of 4-CH_3_C_6_H_4_SO_2_Cl with (CH_3_)_3_CNH_2_ readily gave the desired parent sulphonamide, CH_3_C_6_H_4_SO_2_NHC(CH_3_)_3_, which could be effectively purified by recrystallisation. Unfortunately, treatment of 4-CH_3_C_6_H_4_SO_2_NHC(CH_3_)_3_ with [(CH_3_)_3_COCO]_2_O in the presence of 4-dimethylaminopyridine in dichloromethane gave no reaction, even after 48 h stirring under conditions in which other parent sulphonamides, including 4-CH_3_C_6_H_4_SO_2_NHCH_2_CH(CH_3_)_2_, gave 100% conversion to the *N*-CO_2_C(CH_3_)_3_ derivatives in a period of 10–60 min. Furthermore, attempts to prepare the lower homologue, 4-CH_3_C_6_H_4_SO_2_N[C(CH_3_)_3_]CO_2_CH_3_, were only partially successful, even after repeated treatment of 4-CH_3_C_6_H_4_SO_2_NHC(CH_3_)_3_ with NaH and ClCO_2_CH_3_, under conditions in which isomeric 4-CH_3_C_6_H_4_SO_2_N[CH_2_CH(CH_3_)_2_]CO_2_CH_3_ and 4-CH_3_C_6_H_4_SO_2_N[CH_2_CH(CH_3_)_2_]CO_2_C_2_H_5_ derivatives had been successfully made and purified.

## 4. Materials and Methods

Most of the parent sulphonamides, ArSO_2_NHR^1^, were prepared by dropwise addition of a dichloromethane solution of the corresponding commercial sulphonyl chloride, ArSO_2_Cl, to a magnetically stirred solution of a slight excess (1.1 equivalents) of the requisite amine, R^1^NH_2_ (for R^1^ = C_3_H_7_ or C_4_H_9_) in the presence of triethylamine (1.2–1.5 equivalents) as an acid scavenger, maintaining the temperature at 0–5 °C. Preparation of the lower homologues, with an *N*-CH_3_ or *N*-C_2_H_5_ group was achieved similarly, but with a solution of the parent amine in ethanol or water (in which case tetrahydrofuran was used instead of dichloromethane as the solvent). The addition typically required 20–40 min, after which stirring was continued until TLC showed that the reaction was complete. The crude sulphonamide was then isolated by a standard extractive procedure. In most cases, a solid product was obtained in close to quantitative yield; recrystallisation from ethanol/water (or in the case of 4-CH_3_C_6_H_4_SO_2_NHCH_2_CH_2_CH_3_, from light petroleum (bp: 40–60 °C)) gave pure product, usually in 75–85% yield.

The synthesis of the corresponding carbomethoxy and carboethoxy derivatives was achieved by dropwise addition under a nitrogen atmosphere of a solution of the parent sulphonamide in dry tetrahydrofuran to a magnetically stirred suspension in tetrahydrofuran of an excess (1.1–1.7 equivalents) of sodium hydride that had been washed by decantation with either petroleum ether or tetrahydrofuran to remove the mineral oil. Hydrogen gas was evolved. After allowing stirring to continue for 5–10 min, an excess (1.1–1.5 equivalents) of the requisite chloroformate was added in portions from a syringe. Once the reaction was complete, as revealed by TLC, the mixture was cautiously poured into cold water, and the product was isolated by extraction with dichloromethane. Solid products were purified by recrystallisation from ethanol/water; oils were purified by chromatography on silica, eluting with petroleum ether and ethyl acetate mixtures. Yields of crude product were normally at least 80%.

The carbo-*tert*-butoxy derivatives were prepared by stirring under a nitrogen atmosphere a dichloromethane solution of the parent sulphonamide and a slight excess (1.1–1.2 equivalents) of di-*tert*-butyldicarbonate in the presence of a catalytic quantity (0.02–0.05 equivalents) of 4-dimethylaminopyridine. Once TLC showed that the reaction was complete, water was added, followed by sufficient sodium carbonate solution (10% *w*/*v*) to raise the pH to 9. After stirring for a further 30–90 min, the dichloromethane layer was separated and the aqueous phase was extracted twice with dichloromethane. The combined organic phases were washed with dilute hydrochloric acid (to remove the 4-dimethylaminopyridine), dried (MgSO_4_), filtered and rotary evaporated to constant mass to give the crude product in 80–98% yield. Recrystallisation from ethanol/water gave pure product in 70–85% yield.

The following general procedure for treatment of the orthogonally protected sulphonamides with lithium di-isopropylamine was devised. A fresh solution of lithium di-isopropylamide was prepared by adding di-isopropylamine (0.65 mL) and THF (2.35 mL) to a septum-sealed flame-dried round-bottomed flask that had been flushed with nitrogen. The flask was cooled to −78 °C in a dry-ice/acetone bath and the contents were magnetically stirred as *n*-butyllithium (2.0 mL, 2.5 M in hexane) was added.

In a separate flask, the corresponding orthogonally protected sulphonamide (0.1–6.5 mmol) was dissolved in THF and cooled to −78 °C. Two equivalents of the lithium di-isopropylamine solution were transferred to the magnetically stirred sulphonamide solution by one of two methods. For small scale reactions (0.1–0.5 mmolar), the transfer was made by means of a disposable syringe, which was cooled by drawing up the solution and returning it to the cooled flask several times. For larger scale reactions (1.0–6.5 mmolar), the transfer was through a cannula by reducing the pressure in the flask containing the sulphonamide solution. Two minutes after the transfer was complete, the reaction was quenched by addition of aqueous citric acid solution. After allowing the reaction mixture to attain ambient temperature, the product was extracted with dichloromethane, dried (MgSO_4_), filtered, and rotary evaporated to constant mass to give the crude product at 70–99% yield. Solid products were purified by recrystallisation from ethanol/water; in cases where the reaction gave two products, the components were separated by chromatography on silica, eluting with petroleum ether and ethyl acetate mixtures.

Full characterisation data for all synthesised compounds are available in the Supplementary Materials.

## 5. Conclusions

Taken together, these trends lead to the following conclusions about the selection of the R^1^ and R^2^ groups if the undesirable cyclisation is to be avoided.

Firstly, if the *N*-alkyl group, R^1^, must be small, it is necessary to have a bulky R^2^ group, ideally C(CH_3_)_3_, for which rearrangement of CO_2_R^2^ generally occurs essentially without interference from cyclisation. If both R^1^ and R^2^ are small, cyclisation will compete with rearrangement for a wide range of substituents in the 4-position of the aromatic ring, thus reducing the yield and causing complications in purifying the desired product.

Secondly, if the migrating CO_2_R^2^ entity contains a small R^2^ group, it is desirable to choose a branched R^1^ substituent. In these cases, either CH(CH_3_)_2_ or CH_2_CH(CH_3_)_2_ appear to be equally viable.

Thirdly, although it might seem attractive to exploit the strong steric influence of R^1^ on the competition between the desired rearrangement and the unwanted cyclisation by choosing R^1^ = C(CH_3_)_3_, this option is impracticable because elaboration of the parent sulphonamide (which is easily prepared) to the required CO_2_R^2^ derivatives is much more difficult than for smaller R^1^ groups.

Finally, the two series with R^1^ = CH(CH_3_)_2_ or CH_2_CH(CH_3_)_2_ appear to offer the best prospects of securing clean rearrangement, irrespective of the nature of the migrating CO_2_R^2^ group and the nature of any substituent other than NO_2_ (for which rearrangement does not occur [39]). Analogues with smaller R^1^ groups (CH_3_ or C_2_H_5_) are more easily prepared, but do not always facilitate clean rearrangement without interference from cyclisation. The presence of the much bulkier C(CH_3_)_3_ group would probably lead to exclusive rearrangement, regardless of the nature of the migrating CO_2_R^2^ group, but steric hindrance is so pronounced in these cases that preparing the desired derivatives is much more difficult. The series with R^1^ = CH(CH_3_)_2_ or CH_2_CH(CH_3_)_2_ are advantageous in at least one analytical way: the presence of distinctive signals in the NMR spectra is useful (a one proton septet, and a six proton doublet, when R^1^ = CH(CH_3_)_2_, or a two proton doublet, a one proton nonet, and a six proton doublet, when R^1^ = CH_2_CH(CH_3_)_2_).

## Figures and Tables

**Table 1:** Rearrangement and cyclisation of 4-CH_3_C_6_H_4_SO_2_NR^1^CO_2_R^2^.

Substrate			Products		
			Relative Yield (%) ^a^		Ratio ^a^
Compound	R^1^	R^2^	27	28	27:28
**26ai**	CH_3_	CH_3_	48	52	1:1.1
**26aii**	CH_3_	C_2_H_5_	67	33	2:1
**26aiii**	CH_3_	C(CH_3_)_3_	≳99 ^b^	≲1	≳99:1
**26bi**	C_2_H_5_	CH_3_	80	20	4:1
**26bii**	C_2_H_5_	C_2_H_5_	95	5	19:1
**26biii**	C_2_H_5_	C(CH_3_)_3_	≳99 ^b^	≲1	≳99:1
**26ci**	CH_2_CH_2_CH_3_	CH_3_	95	~5	~20:1
**26cii**	CH_2_CH_2_CH_3_	C_2_H_5_	98	2	~50:1
**26ciii**	CH_2_CH_2_CH_3_	C(CH_3_)_3_	≳99 ^b^	≲1	≳99:1
**26di**	CH(CH_3_)_2_	CH_3_	~98	~2	~50:1
**26dii**	CH(CH_3_)_2_	C_2_H_5_	≳99 ^b^	≲1	≳99:1
**26diii**	CH(CH_3_)_2_	C(CH_3_)_3_	≳99 ^b^	≲1	≳99:1
**26ei**	CH_2_CH(CH_3_)_2_	CH_3_	≳98	≳1	~98:1
**26eii**	CH_2_CH(CH_3_)_2_	C_2_H_5_	≳99 ^b^	≲1	≳99:1
**26eiii**	CH_2_CH(CH_3_)_2_	C(CH_3_)_3_	≳99 ^b^	≲1	≳99:1

^a^ Yields determined from the relative intensity of the relevant signals in the ^1^H NMR spectrum of the crude mixture of products; the quoted ratios (a measure of chemoselectivity) are the means of at least two independent reactions. Relative yield values are rounded to the nearest integer; the quoted ratio is taken from the unrounded relative yields. ^b^ Only a single product was detected by ^1^H NMR.

**Table 2:** Rearrangement and cyclisation of C_6_H_5_SO_2_NR^1^CO_2_R^2^.

Substrate			Products		
			Relative Yield (%) ^a^		Ratio ^a^
Compound	R^1^	R^2^	27	28	27:28
**26fi**	CH_3_	CH_3_	90	10	9:1
**26fii**	CH_3_	C(CH_3_)_3_	≳99 ^b^	≲1	≳99:1
**26gi**	CH(CH_3_)_2_	CH_3_	≳99 ^b^	≲1	≳99:1
**26gii**	CH(CH_3_)_2_	C_2_H_5_	≳99 ^b^	≲1	≳99:1
**26giii**	CH(CH_3_)_2_	C(CH_3_)_3_	≳99 ^b^	≲1	≳99:1
**26hi**	CH_2_CH(CH_3_)_2_	CH_3_	~98	2	~50:1
**26hii**	CH_2_CH(CH_3_)_2_	C_2_H_5_	≳99 ^b^	≲1	≳99:1
**26hiii**	CH_2_CH(CH_3_)_2_	C(CH_3_)_3_	≳99 ^b^	≲1	≳99:1

^a^ Yields determined from the relative intensity of the relevant signals in the ^1^H NMR spectrum of the crude mixture of products; the quoted ratios (a measure of chemoselectivity) are the means of at least two independent reactions. Relative yield values are rounded to the nearest integer; the quoted ratio is taken from the unrounded relative yields. ^b^ Only a single product was detected by ^1^H NMR.

**Table 3:** Rearrangement and cyclisation of 4-XC_6_H_4_SO_2_NR^1^CO_2_R^2^.

Substrate				Products		
				Relative Yield (%) ^a^		Ratio ^a^
Compound	X	R^1^	R^2^	27	28	27:28
**26ji**	F	CH_3_	CH_3_	95	5	17:1
**26jii**	F	CH_3_	C(CH_3_)_3_	99	1	99:1
**26jiii**	F	CH(CH_3_)_2_	CH_3_	~99	~1	~95:1
**26jiv**	F	CH(CH_3_)_2_	C_2_H_5_	99	1	99:1
**26jv**	F	CH(CH_3_)_2_	C(CH_3_)_3_	≳99 ^b^	≲1	≳99:1
**26ki**	Cl	CH_3_	CH_3_	94	6	14:1
**26kii**	Cl	CH_3_	C(CH_3_)_3_	≳99 ^b^	≲1	≳99:1
**26kiii**	Cl	CH(CH_3_)_2_	CH_3_	~99	~1	98:1
**26kiv**	Cl	CH(CH_3_)_2_	C_2_H_5_	≳99 ^b^	≲1	≳99:1
**26kv**	Cl	CH(CH_3_)_2_	C(CH_3_)_3_	≳99 ^b^	≲1	≳99:1
**26li**	Br	CH_3_	CH_3_	90	10	9:1
**26lii**	Br	CH_3_	C(CH_3_)_3_	~99	~1	~99:1
**26liii**	Br	CH(CH_3_)_2_	CH_3_	~99	~1	~99:1
**26liv**	Br	CH(CH_3_)_2_	C_2_H_5_	≳99 ^b^	≲1	≳99:1
**26lv**	Br	CH(CH_3_)_2_	C(CH_3_)_3_	≳99 ^b^	≲1	≳99:1
**26mi**	CH_3_O	CH_3_	CH_3_	88	12	7:1
**26mii**	CH_3_O	CH_3_	C(CH_3_)_3_	~99	~1	~99:1
**26miii**	CH_3_O	CH(CH_3_)_2_	CH_3_	~99	~1	~99:1
**26miv**	CH_3_O	CH(CH_3_)_2_	C_2_H_5_	≳99 ^b^	≲1	≳99:1
**26mv**	CH_3_O	CH(CH_3_)_2_	C(CH_3_)_3_	≳99 ^b^	≲1	≳99:1
**26ni**	CF_3_	CH_3_	CH_3_	90	10	9:1
**26nii**	CF_3_	CH_3_	C(CH_3_)_3_	≳99 ^b^	≲1	≳99:1
**26niii**	CF_3_	CH(CH_3_)_2_	CH_3_	~99	~1	~99:1
**26niv**	CF_3_	CH(CH_3_)_2_	C(CH_3_)_3_	≳99 ^b^	≲1	≳99:1
**26oi**	CN	CH_3_	CH_3_	93	7	13:1
**26oii**	CN	CH_3_	C(CH_3_)_3_	~99	~1	99:1
**26oiii**	CN	CH(CH_3_)_2_	CH_3_	≳99 ^b^	≲1	≳99:1
**26oiv**	CN	CH(CH_3_)_2_	C_2_H_5_	≳99 ^b^	≲1	≳99:1
**26ov**	CN	CH(CH_3_)_2_	C(CH_3_)_3_	≳99 ^b^	≲1	≳99:1
**26pi**	C(CH_3_)_3_	CH_3_	CH_3_	92	8	11:1
**26pii**	C(CH_3_)_3_	CH_3_	C(CH_3_)_3_	~99	~1	~99:1
**26piii**	C(CH_3_)_3_	CH(CH_3_)_2_	CH_3_	≳99 ^b^	≲1	≳99:1
**26piv**	C(CH_3_)_3_	CH(CH_3_)_2_	C(CH_3_)_3_	≳99 ^b^	≲1	≳99:1

^a^ Yields determined from the relative intensity of the relevant signals in the ^1^H NMR spectrum of the crude mixture of products; the quoted ratios (a measure of chemoselectivity) are the means of at least two independent reactions. Relative yield values are rounded to the nearest integer; the quoted ratio is taken from the unrounded relative yields. ^b^ Only a single product was detected by ^1^H NMR.

## Data Availability

The original contributions presented in this study are included in the article/Supplementary Materials. Further inquiries can be directed to the corresponding author.

## Supplementary Materials

The following supporting information can be downloaded at: https://www.mdpi.com/article/10.3390/molecules30081823/s1, further details of the synthetic procedures, including representative examples and characterisation data.
