# Supplementary material for: Steric Effects of N-Alkyl Group on the Base-Induced Nitrogen to Carbon Rearrangement of Orthogonally Protected N-Alkyl Arylsulphonamides
Source: Molecules. 2025 Apr 18;30(8):1823. doi: 10.3390/molecules30081823 (PMC12029463; doi:10.3390/molecules30081823)
Supplement: Supplementary file 1 [file molecules-30-01823-s001.zip › molecules-3372589-supplementary.pdf]

Steric Effects of *N*-Alkyl Group on the Base-induced Nitrogen to Carbon Rearrangement of Orthogonally Protected N-Alkyl Arylsulphonamides

Amie Saidykhan, Jenessa Ebert, Nathan W. Fenwick, William H. C. Martin and Richard D. Bowen *

School of Chemistry and Biosciences, Faculty of Life Sciences, University of Bradford, Bradford BD7 1DP, UK;

Supplementary Data

1 General information

Accurate molecular mass information was obtained on $[M+H]^+$ or $[M+Na]^+$ ions formed in a Orbitrap instrument operating under positive ion electrospray ionization conditions.

^1H and ^{13}C NMR spectra were recorded on a Bruker instrument operating at 400 MHz. Raw data can be found at the NMRXiv repository link: https://nmrxiv.org/project/JoegihHGrEzSAYQhBWktr46iLlWGZ4Bj7MA9Auap

Melting points were determined on a Gallenkamp melting point apparatus. Data are uncorrected.

Compounds not characterised in the literature are reported with an asterisk “*”

Crystalline compounds identified as white (to the naked eye) were colourless and transparent when examined through a magnifying glass or microscope.

2 Synthesis of parent sulphonamides, $XC_6H_4SO_2NHR^1$

Detailed illustrative examples: **30b**, **30j** and **30k**.

30a

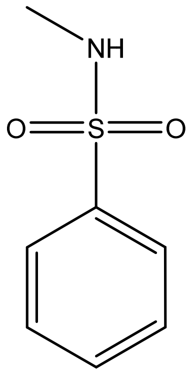

Prepared from benzenesulphonyl chloride (30.8 g, 174 mmol) in THF (60 mL) and aqueous methylamine (40% w/v, 60 mL, 770 mmol) and triethylamine (28.2 g, 280 mmol) in THF (200 mL) in the manner described in illustrative procedure 2. The product was an almost colourless oil (29.2 g, 91.7%), which could be recrystallised from ethanol/water at low temperatures (ca -25 °C), but melted on being allowed to attain ambient temperature (mp lit 30 °C). This material was pure by ^{1}H NMR and was satisfactory for elaboration into its $PhSO_2N(CH_3)CO_2R$ derivatives.

30b

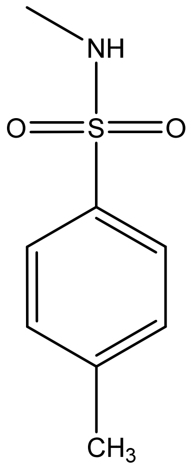

Illustrative procedure 1

A solution of 4-toluenesulphonyl chloride (19.1 g, 100 mmol) in dichloromethane (100 ml) was added dropwise during 75 minutes to a magnetically stirred solution of methylamine in ethanol (33%, w/v, 15 ml, ~ 125 mmol) and triethylamine (20.2 g, 198 mmol) in dichloromethane (150 ml) at 5-6 °C under a nitrogen atmosphere in a 1 litre, three necked, round-bottomed flask in an ice/water bath. After a further 1 hour string, during which a white precipitate of amine hydrochloride formed, tlc (on silica, eluting with 25% ethyl acetate in petroleum ether), showed no starting material, only a slower running spot for product. The mixture was poured onto ice/water (300 ml) and acidified with dilute hydrochloric acid. The organic phase was separated and the aqueous phase was extracted with dichloromethane (3 x 50 ml). The combined organic phases were dried ($MgSO_4$), filtered and evaporated to constant mass to give a white solid (19.0 g, 102%), which was recrystallised from ethanol/water to give white plates (16.3 g, 83%), mp 78-9 °C (lit. 78-9 °C).

HRMS (ESI) *m/z* calcd for $C_8H_{12}O_2NS^+$: 186.05833 $[M+H]^+$; found: 186.05779.

30c

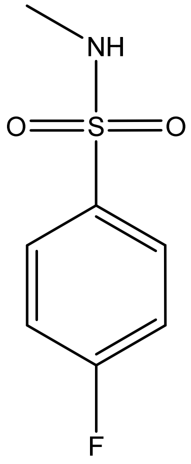

Prepared from 4-fluorobenzenesulphonyl chloride (23.5 g, 120 mmol) in THF (60 mL) and aqueous methylamine (40% w/v, 25 mL, 290 mmol) and triethylamine (19.0 g, 188 mmol) in THF (200 mL) in the manner described in illustrative procedure 2.

The product was a pale yellow oil (22.4 g, 93%), which solidified overnight to an almost white crystalline mass. Recrystallisation from ethanol/water gave white needles (19.5 g, 80.5%), mp 76-7 °C, lit 76 °C.

HRMS (ESI) *m/z* calcd for $C_7H_9O_2NSF^+$: 290.03325 $[M+H]^+$; found: 190.03320.

30d

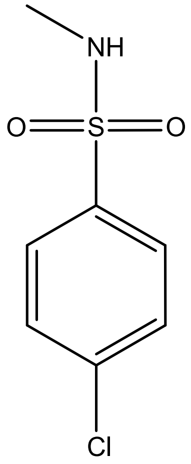

Prepared from 4-chlorobenzenesulphonyl chloride (22.5 g, 106 mmol) in THF (50 mL) and aqueous methylamine (40% w/v, 25 mL, 290 mmol) and triethylamine (22.0 g, 218 mmol) in THF (200 ml) in the manner described in illustrative procedure 2. The product was a white solid (22.4 g, 92.8%). Recrystallisation from ethanol/water gave white needles (17.0 g, 77.5%), mp 63-4 °C, lit 62-4 °C.

HRMS (ESI) *m/z* calcd for $C_7H_9O_2NSBrNa^+$: 227.98565 $[M+Na]^+$; found: 228.10472.

30e

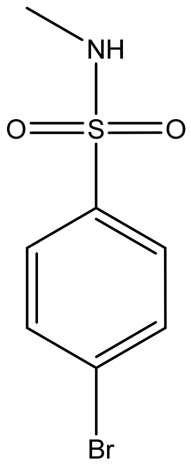

Prepared from 4-bromobenzenesulphonyl chloride (27.5 g, 107 mmol) in THF (80 mL) and aqueous methylamine (40% w/v, 25 mL, 290 mmol) and triethylamine (21.0 g, 208 mmol) in THF (200 ml) in the manner described in illustrative procedure 2. The product was a white solid (25.1 g, 93.4%). Recrystallisation from ethanol/water gave white needles (22.1 g, 82.2%), mp 74-5 °C, lit 74 °C.

HRMS (ESI) *m/z* calcd for $C_7H_9O_2NSBr^+$: 249.95319 $[M+H]^+$; found: 249.95317.

30f

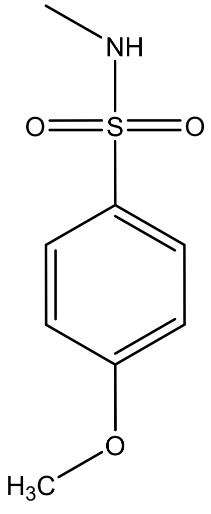

Prepared from 4-methoxybenzenesulphonyl chloride (20.5 g, 100 mmol) in THF (80 mL) and aqueous methylamine (40% w/v, 25 mL, 290 mmol) and triethylamine (20.2

g, 200 mmol) in THF (200 ml) in the manner described in illustrative procedure 2. The product was a white solid (19.5 g, 97.7%). Recrystallisation from ethanol/water gave white needles (18.8 g, 94.2%), mp 96-7 °C, lit 94-5 °C.

HRMS (ESI) *m/z* calcd for $C_8H_{12}O_3NS^+$: 202.05324 $[M+H]^+$; found: 202.05302.

30g

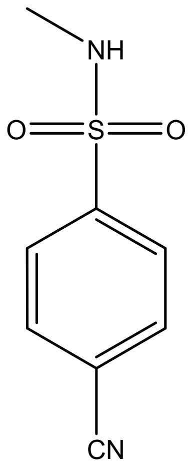

Prepared from 4-cyanobenzenesulphonyl chloride (20.5 g, 102 mmol) in THF (100 mL) and aqueous methylamine (40% w/v, 25 mL, 290 mmol) and triethylamine (21.0 g, 208 mmol) in THF (200 ml) in the manner described in illustrative procedure 2. The product was a cream solid (19.0 g, 90.2%). Recrystallisation from ethanol/water gave pale cream plates (18.0 g, 85.5%), mp 124-5 °C, lit 127-8 °C.

30h

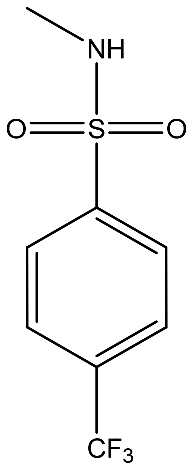

Prepared from 4-trifluoromethylbenzenesulphonyl chloride (25.0 g, 102 mmol) in THF (60 mL) and aqueous methylamine (40% w/v, 25 mL, 290 mmol) and triethylamine (22.5 g, 223 mmol) in THF (200 ml) in the manner described in illustrative procedure 2. The product was a pale yellow oil (23.1 g, 95.4%). Recrystallisation from ethanol/water gave white needles (20.9 g, 86.3%), mp 78-9 °C, lit 81-3 °C.

HRMS (ESI) *m/z* calcd for $C_8H_9O_2NSF_3Na^+$: 262.01201 $[M+Na]^+$; found: 262.01193.

30i*

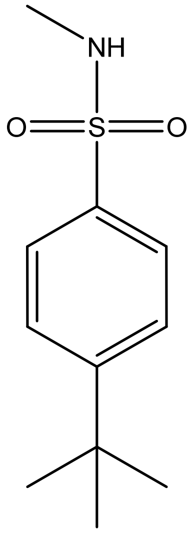

Prepared from 4-*t*-butylbenzenesulphonyl chloride (20.7 g, 106 mmol) in THF (80 mL) and aqueous methylamine (40% w/v, 25 mL, 290 mmol) and triethylamine (19.5 g, 193 mmol) in THF (200 ml) in the manner described in illustrative procedure 2. The product was a pale oil (23.1 g, 91.2%), which slowly solidified on standing overnight. Recrystallisation from ethanol/water gave white crystals (21.8 g, 86.1%), mp 118-9 °C.

HRMS (ESI) *m/z* calcd for $C_{11}H_{18}O_2NS^+$: 228.10528 $[M+H]^+$; found: 228.10468.

30j

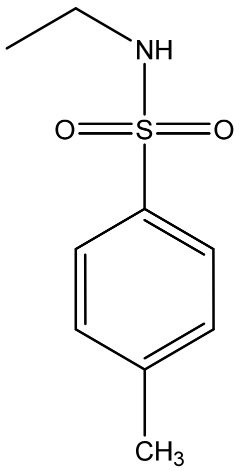

Illustrative procedure 2

A solution of 4-toluenesulphonyl chloride (19.5 g, 102 mmol) in tetrahydrofuran (100 ml) was added dropwise during 100 minutes to a magnetically stirred solution of ethylamine in water (70%, w/v, 15 ml, ~ 125 mmol) and triethylamine (20.2 g, 198 mmol) in tetrahydrofuran (150 ml) at 5-8 °C under a nitrogen atmosphere in a 1 litre, three necked, round-bottomed flask in an ice/water bath. After a further 3 hour string, during which a white precipitate of amine hydrochloride formed, tlc (on silica, eluting with 25% ethyl acetate in petroleum ether), showed no starting material, only a slower running spot for product. The mixture was diluted with a large volume of water (600 ml) and acidified with dilute hydrochloric acid. After cooling the warm solution, the product was extracted with dichloromethane (4 x 80 ml). The combined organic phases were washed with aqueous sodium carbonate solution (5% w/v, 50 ml), dried ($MgSO_4$), filtered and evaporated to constant mass to give a colourless oil, which solidified on standing to a white solid (20.4 g, 100%), which was recrystallised from ethanol/water to give white plates (19.3 g, 90%), mp 64-5 °C (lit. 64 °C).

HRMS (ESI) *m/z* calcd for $C_9H_{14}O_2NS^+$: 200.07398 $[M+H]^+$; found: 200.07320.

30k

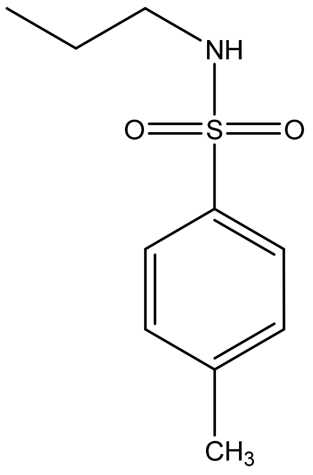

Illustrative procedure 3

A solution of 4-toluenesulphonyl chloride (30.0 g, 157 mmol) in dichloromethane (150 ml) was added dropwise during 25 minutes to a magnetically stirred solution of n-propylamine (10.5 g, 178 mmol) and triethylamine (20.2 g, 200 mmol) in dichloromethane (300 ml) at 0-5 °C under a nitrogen atmosphere in a 1 litre, three necked, round-bottomed flask in an ice/water bath. After a further 2 hours string, during which the mixture was allowed to attain ambient temperature (30 °C), tlc (on silica, eluting with 25% ethyl acetate in petroleum ether), showed no starting material, only a slower running spot for product. The mixture was diluted with a large volume of water (600 ml) and acidified with dilute hydrochloric acid. The mixture was poured onto ice/water (500 ml) and acidified with dilute hydrochloric acid. The organic phase was separated and the aqueous phase was extracted with dichloromethane (3 x 80 ml). The combined organic phases were dried ($MgSO_4$), filtered and evaporated to constant mass to give a pale yellow oil (37.9 g, 100%), which solidified on standing to a white crystalline mass, mp 49 – 50 °C (lit 52 °C), which could not be effectively recrystallised from ethanol/water (crystals were formed on chilling, but they reverted to an oil on warming to ambient temperature). This

material was sufficiently pure for elaboration to the requisite derivatives. Recrystallisation of a portion from a large volume of light petroleum (40 – 60 °C fraction) gave white crystals, mp 51 – 52 °C (lit 52 °C).

HRMS (ESI) *m/z* calcd for $C_{10}H_{16}O_2NS^+$: 214.08963 [M+H]$^+$; found: 214.08969.

30l

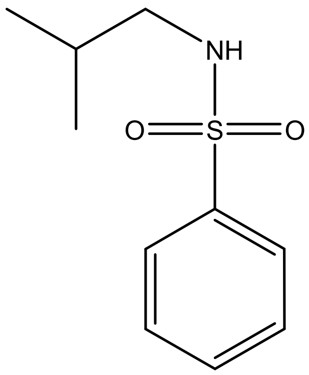

Prepared from benzenesulphonyl chloride (35.5 g, 201 mmol) in dichloromethane (60 ml) and iso-butylamine (18.3 g, 250 mmol) and triethylamine (25.2 g, 250 mmol) dichloromethane (300 ml) in the manner described in illustrative procedure 3. An almost colourless oil (43.2 g, 99%) was obtained, which solidified on standing for 48 hours; recrystallisation from ethanol/water gave white leaflets (33.5g, 77%), mp 52-3 °C, (lit 53 °C).

HRMS (ESI) *m/z* calcd for $C_{10}H_{16}O_2NS^+$: 214.08963 [M+H]$^+$; found: 214.09297.

30m

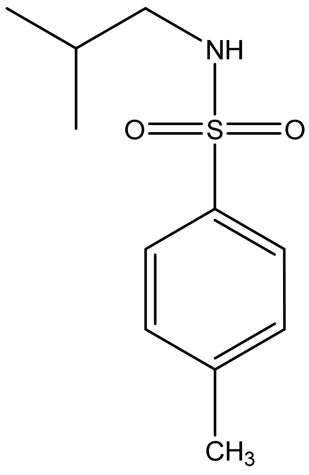

Prepared from 4-toluenesulphonyl chloride (39.0 g, 204 mmol) in dichloromethane (150 ml) and isobutylamine (16.0 g, 226 mmol) and triethylamine (27.6 g, 272 mmol) dichloromethane (250 ml) in the manner described in illustrative procedure 3. A colourless oil that quickly solidified to a white solid (43.8 g, 95%), which was sufficiently pure for elaboration to the required derivatives. The residual material (30.11 g) was recrystallised from ethanol/water to give white needles (24.5 g, 78%), mp 78-9 °C, (lit 78 °C).

HRMS (ESI) *m/z* calcd for $C_{11}H_{18}O_2NS^+$: 228.10528 $[M+H]^+$; found: 228.10448.

30n

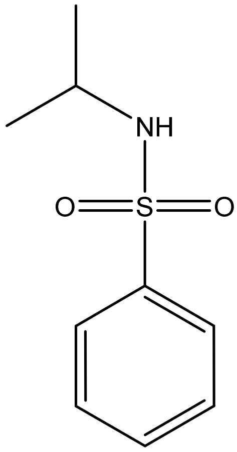

Prepared from benzenesulphonyl chloride (38.1 g, 215 mmol) in dichloromethane (80 ml) and iso-propylamine (14.5 g, 245 mmol) and triethylamine (26.5 g, 262 mmol) dichloromethane (100 ml) in the manner described in illustrative procedure 3. A very pale yellow oil (43.7 g, 102%) was obtained, which did not solidify, but was sufficiently pure for elaboration into the required derivatives. [Lit mp 30 °C.]

HRMS (ESI) *m/z* calcd for $C_9H_{14}O_2NS^+$: 200.07398 $[M+H]^+$; found: 200.07373.

30o

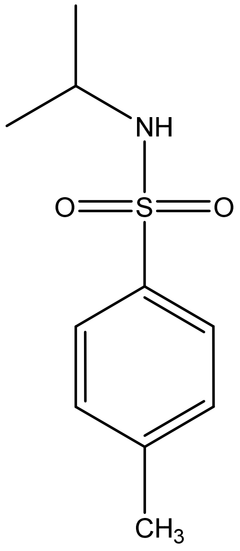

Prepared from 4-toluenesulphonyl chloride (38.1 g, 200 mmol) in dichloromethane (150 ml) and isopropylamine (14.0 g, 237 mmol) and triethylamine (25.3 g, 250 mmol) dichloromethane (250 ml) in the manner described in illustrative procedure 3. A colourless oil (42.2 g, 99%) was obtained that solidified on standing and was recrystallised from ethanol/water to give white needles (34.5 g, 81%), mp 50-1 °C, (lit 51 °C).

HRMS (ESI) *m/z* calcd for $C_{10}H_{16}O_2NS^+$: 214.08963 $[M+H]^+$; found: 214.09311.

30p*

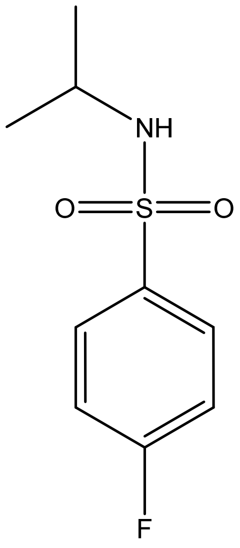

Prepared from 4-fluorobenzenesulphonyl chloride (25.0 g, 128 mmol) in dichloromethane (100 ml) and iso-propylamine (25.0 g, 423 mmol) and dichloromethane (200 ml) in the manner described in illustrative procedure 3. A white solid (27.3 g, 97.9%) was obtained, was recrystallised from ethanol/water to give white needles (21.4 g, 76.7%), mp 80-1 °C.

HRMS (ESI) *m/z* calcd for $C_9H_{13}O_2NSF^+$: 218.06455 $[M+H]^+$; found: 218.06464.

30q

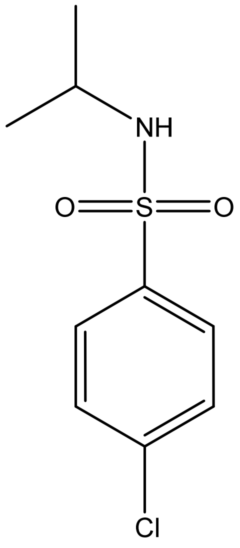

Prepared from 4-chlorobenzenesulphonyl chloride (21.2 g, 100 mmol) in dichloromethane (100 ml) and iso-propylamine (14.5 g, 245 mmol) and dichloromethane (150 ml) in the manner described in illustrative procedure 3. A white solid (22.9 g, 97.7%) was obtained, was recrystallised from ethanol/water to give white needles (21.4 g, 87.4%), mp 88-9 °C, lit 100-1 °C.

HRMS (ESI) *m/z* calcd for $C_9H_{13}O_2NSCl^+$: 234.03500 $[M+H]^+$; found: 234.03481.

30r

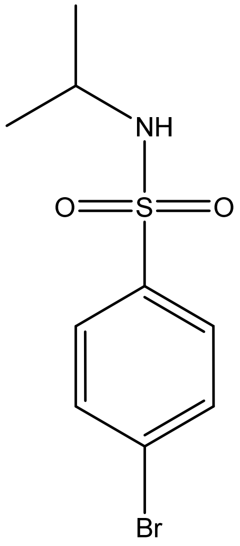

Prepared from 4-bromobenzenesulphonyl chloride (25.5 g, 100 mmol) in dichloromethane (100 ml) and iso-propylamine (16.2 g, 274 mmol) and dichloromethane (150 ml) in the manner described in illustrative procedure 3. A white solid (27.5 g, 98.9%) was obtained, which was recrystallised from ethanol/water to give white needles (22.3 g, 80.2%), mp 99-100 °C, lit 105-6°C.

HRMS (ESI) *m/z* calcd for $C_9H_{13}O_2NSBr^+$: 277.98449 $[M+H]^+$; found: 277.98419.

30s

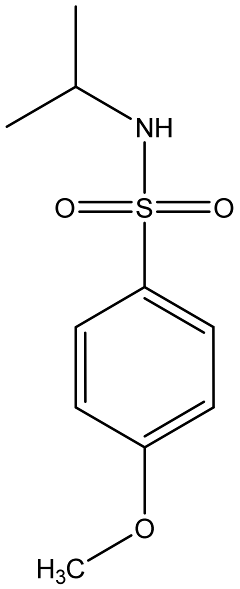

Prepared from 4-methoxybenzenesulphonyl chloride (24.3 g, 117 mmol) in dichloromethane (100 ml) and iso-propylamine (9.5 g, 161 mmol), triethylamine (15.0 g, 148 mmol) and dichloromethane (200 ml) in the manner described in illustrative procedure 3. An almost colourless oil (22.9 g, 85.0%) was obtained, which solidified on standing overnight. This material was pure by ^{1}H NMR; it was difficult to recrystallise from ethanol/water, but was satisfactory for derivatisation. Slow recrystallisation from a large volume of petroleum ether (bp 40-60 °C fraction) gave white leaflets, mp 58-9 °C, lit 55-7 °C.

HRMS (ESI) *m/z* calcd for $C_{10}H_{16}O_3NS^+$: 230.08454 $[M+H]^+$; found: 230.09414.

30t*

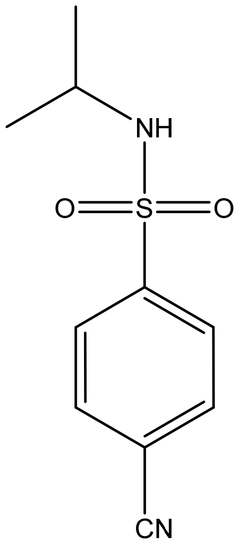

Prepared from 4-cyanobenzenesulphonyl chloride (14.3 g, 70.9 mmol) in dichloromethane (50 ml) and iso-propylamine (7.2 g, 122 mmol), triethylamine (10.2 g, 101 mmol) and dichloromethane (130 ml) in the manner described in illustrative procedure 3. An almost colourless oil (16.1 g, 101%) was obtained, which quickly solidified. Recrystallisation from ethanol/water gave fine white crystals (12.5 g, 78.6%), mp 90-1 °C.

HRMS (ESI) *m/z* calcd for $C_{10}H_{12}O_2N_2SNa^+$: 247.05117 $[M+Na]^+$; found: 247.05128.

30u*

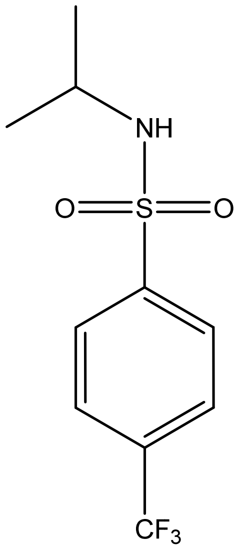

Prepared from 4-trifluoromethylbenzenesulphonyl chloride (10.1 g, 41.3 mmol) in dichloromethane (35 ml) and iso-propylamine (7.65 g, 129 mmol), triethylamine (11.3 g, 112 mmol) and dichloromethane (75 ml) in the manner described in illustrative procedure 3. An almost colourless oil (11.0 g, 99.7%) was obtained, which solidified on exposure to diaphragm pump pressure. Recrystallisation from ethanol/water gave long white needles (9.45 g, 85.6%), mp 68-9 °C.

HRMS (ESI) *m/z* calcd for $C_{10}H_{13}O_2NSF_3^+$: 268.06136 $[M+H]^+$; found: 268.06116.

30v*

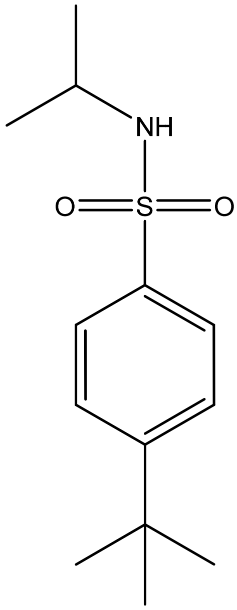

Prepared from 4-t-butylbenzenesulphonyl chloride (25.1 g, 108 mmol) in dichloromethane (100 ml) and iso-propylamine (8.59 g, 145 mmol), triethylamine (12.5 g, 123 mmol) and dichloromethane (150 ml) in the manner described in illustrative procedure 3. An off-white solid (26.5 g, 96.3%) was obtained. Recrystallisation from ethanol/water gave white crystals (25.1 g, 91.2%), mp 107-8 °C.

HRMS (ESI) *m/z* calcd for $C_{13}H_{22}O_2NS^+$: 256.13658 $[M+H]^+$; found: 256.13626.

3. Synthesis of derivatised sulphonamides

Illustrative procedures: **26ai**, **ii**, and **iii**.

26ai

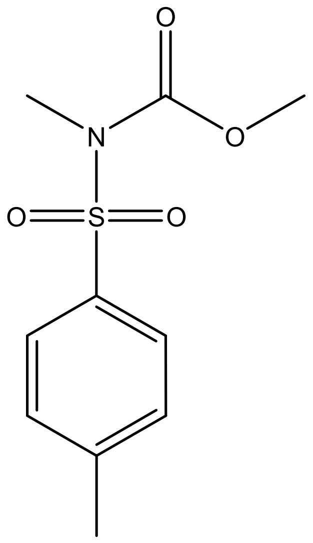

Illustrative procedure 4

A solution of N-methyl-4-toluenesulphonamide, **30b**, (3.72 g, 20.1 mmol) in tetrahydrofuran (50 ml) was added dropwise from a pressure equalising dropping funnel during 15 minutes to a magnetically stirred suspension in tetrahydrofuran (50 ml) of sodium hydride (60% dispersion in mineral oil, 0.95 g, 23.7 mmol, that had been washed by decantation twice with petroleum ether, 10 ml) under a nitrogen atmosphere in a 250 ml three necked round bottomed flask equipped with a septum cap and a balloon adaptor. Hydrogen gas was evolved. After a further 5 minutes string, methyl chloroformate (3.80 g, 40.2 mmol) was added in four portions from a syringe through the septum cap. A fine white precipitate formed in the flask. After a further 45 minutes string, tlc (on silica, eluting with 33% ethyl acetate in petroleum ether), showed only a trace of starting material, with a faster running spot for product. The mixture was cautiously poured onto cold water (400 ml) and the product was extracted with dichloromethane (4 x 75 ml). The combined organic extracts

were washed sequentially with aqueous sodium carbonate solution (10% w/v, 50 ml) and dilute hydrochloric acid (1M, 50 ml), dried ($MgSO_4$), filtered and evaporated to constant mass to give a very pale yellow oil (4.82 g, 99%), which solidified on standing for several days. Recrystallisation from ethanol/water gave white needles (3.66 g, 75%), mp 69-70 °C (lit 75-6 °C).

HRMS (ESI) *m/z* calcd for $C_{10}H_{14}O_4NS^+$: 244.06381 $[M+H]^+$; found: 244.06302.

26aii

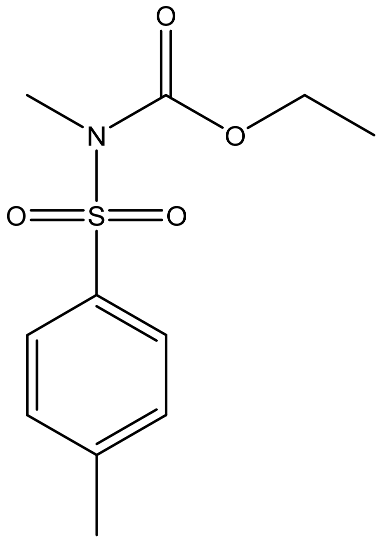

Illustrative procedure 5

A solution of N-methyl-4-toluenesulphonamide, **30b**, (3.70 g, 20.0 mmol) in tetrahydrofuran (40 ml) was added dropwise from a pressure equalising dropping funnel during 10 minutes to a magnetically stirred suspension in tetrahydrofuran (60 ml) of sodium hydride (60% dispersion in mineral oil, 1.11 g, 27.7 mmol, that had been washed by decantation twice with petroleum ether, 10 ml) under a nitrogen atmosphere in a 250 ml three necked round bottomed flask equipped with a septum cap and a balloon adaptor. Hydrogen gas was evolved and a precipitate began to form in the flask. After a further 10 minutes string, ethyl chloroformate 4.71 g, 43.4

mmol) was added in five portions from a syringe through the septum cap, inducing the dissolution of most of the precipitate. After a further 60 minutes string, tlc (on silica, eluting with 33% ethyl acetate in petroleum ether), showed no starting material, only a faster running spot for product. The mixture was cautiously poured onto cold water (400 ml) and the product was extracted with dichloromethane (4 x 75 ml). The combined organic extracts were washed sequentially with aqueous sodium carbonate solution (10% w/v, 50 ml) and dilute hydrochloric acid (1M, 50 ml), dried ($MgSO_4$), filtered and evaporated to constant mass to give a pale yellow oil (5.13 g, 100%) which solidified to a white solid, which could not be recrystallised, mp 35-6 °C (lit 105-7 °C).

HRMS (ESI) *m/z* calcd for $C_{11}H_{16}O_4NS^+$: 258.07946 $[M+H]^+$; found: 258.07889.

26aiii*

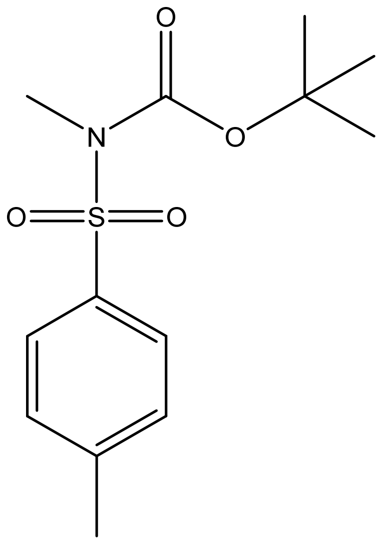

Illustrative procedure 6

A solution of N-methyl-4-toluenesulphonamide, **30b**, (3.60 g, 19.5 mmol), di-t-butyldicarbonate (5.50, 25.2 mmol) and 4-dimethylaminopyridine (0.20 g, 1.6 mmol) in dichloromethane (100 ml) was stirred magnetically under a nitrogen atmosphere.

After 50 minutes, tlc (on silica, eluting with 33% ethyl acetate in petroleum ether), showed no starting material, only a faster running spot for product. Water (100 ml) was added, followed by sufficient aqueous sodium carbonate solution (10% w/v, ~ 0.5 ml) to raise the pH to 9. After string for 90 minutes, the organic phase was separated and the aqueous phase was extracted with dichloromethane (2 x 50 ml). The combined organic extracts were washed sequentially with aqueous sodium carbonate solution (10% w/v, 25 ml) and dilute hydrochloric acid (1M, 25 ml), dried ($MgSO_4$), filtered and evaporated to constant mass to give a white solid (5.66 g, 102%), which was recrystallised from ethanol/water to give white needles (4.84 g, 87%), mp 73-4 °C.

HRMS (ESI) *m/z* calcd for $C_{13}H_{19}O_4NNaS^+$: 308.09270 $[M+Na]^+$; found: 308.09540.

26bi*

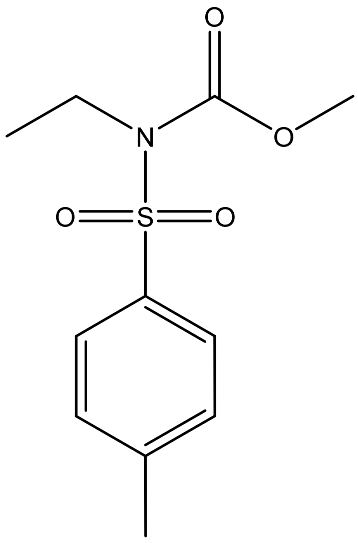

Prepared from N-ethyl-4-toluenesulphonamide, **30j**, (4.01 g, 20.1 mmol) and sodium hydride (60% dispersion in mineral oil, 1.40 g, 35.0 mmol) and methyl chloroformate (5.10 g, 54.0 mmol) in the manner described in illustrative procedure 5. An almost colourless oil (6.01 g) was obtained, which solidified on standing for several days. Recrystallisation from ethanol/water gave white crystals (4.37 g, 83.2 %).

HRMS (ESI) *m/z* calcd for $C_{12}H_{18}O_4NS^+$: 272.09511 $[M+H]^+$; found: 272.09448.

26bii*

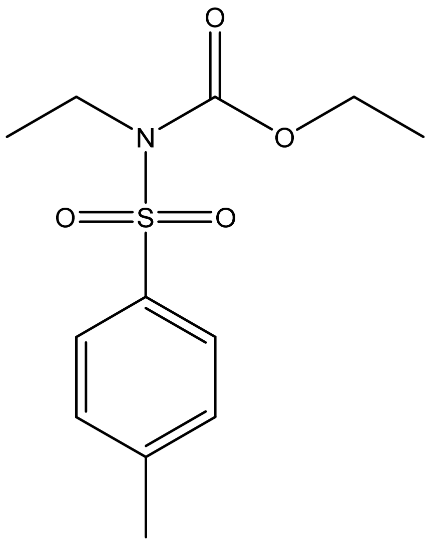

Prepared from N-ethyl-4-toluenesulphonamide, **30j**, (4.01 g, 20.1 mmol) and sodium hydride (60% dispersion in mineral oil, 1.40 g, 35.0 mmol) and ethyl chloroformate (5.80 g, 55.5 mmol) in the manner described in illustrative procedure 5. A gelatinous precipitate which impeded string formed in the flask, but addition of the chloroformate dispersed this material and led to efficient string. A pale yellow oil (5.30 g, 97.3 %) was obtained, which contained residual ethyl chloroformate. Chromatography (on SiO_2, eluting with ethyl acetate in petroleum ether) gave pure product as a colourless oil (4.60 g, 84%).

HRMS (ESI) *m/z* calcd for $C_{12}H_{18}O_4NS^+$: 272.09511 $[M+H]^+$; found: 272.09448.

26biii*

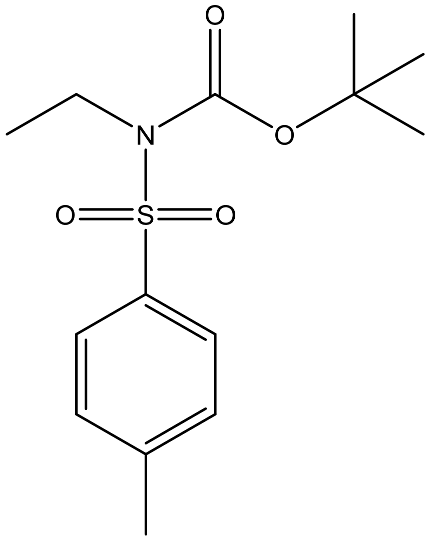

Prepared from N-ethyl-4-toluenesulphonamide, **30j**, (4.02 g, 20.2 mmol), di-t-butyldicarbonate (6.01, 27.5 mmol) and 4-dimethylaminopyridine (0.20 g, 1.6 mmol) in the manner described in illustrative procedure 6.   An almost colourless oil (5.87 g, 97%) was obtained, which solidified on standing for several days; slow recrystallisation from ethanol/water gave white needles (3.73 g, 62%), mp 42-3 °C.

HRMS (ESI) *m/z* calcd for $C_{14}H_{21}O_4NNaS^+$: 322.10835 $[M+Na]^+$; found: 322.11099.

26ci*

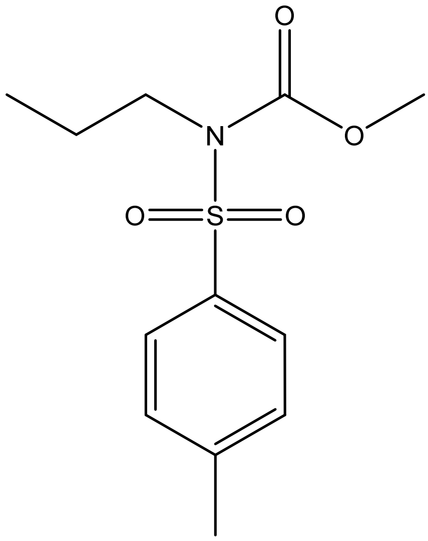

Prepared from unrecrystallised N-n-propyl-4-toluenesulphonamide, **30k**, (6.38 g, 29.9 mmol) and sodium hydride (60% dispersion in mineral oil, 2.40 g, 60.0 mmol) and methyl chloroformate (6.61 g, 69.9 mmol) in the manner described in illustrative procedure 4. Unfortunately, despite using a larger volume of tetrahydrofuran (250 ml total), a gelatinous precipitate which impeded string formed in the flask, but addition of the chloroformate dispersed this material and led to efficient string.   An almost colourless oil (9.20 g, 113%) was obtained, which contained both starting material and residual methyl chloroformate. Treatment of this material with a second portion of sodium hydride (60% dispersion in mineral oil, 0.80 g, 20.0 mmol) and methyl chloroformate (2.20 g, 23.2 mmol) in the same manner gave a pale yellow oil (8.70 g, 108%), which quickly solidified. Recrystallisation from ethanol/water gave white needles (6.05 g, 74%), mp 72-4 °C.

HRMS (ESI) *m/z* calcd for $C_{12}H_{18}O_4NS^+$: 272.09511 $[M+H]^+$; found: 272.09500.

26cii*

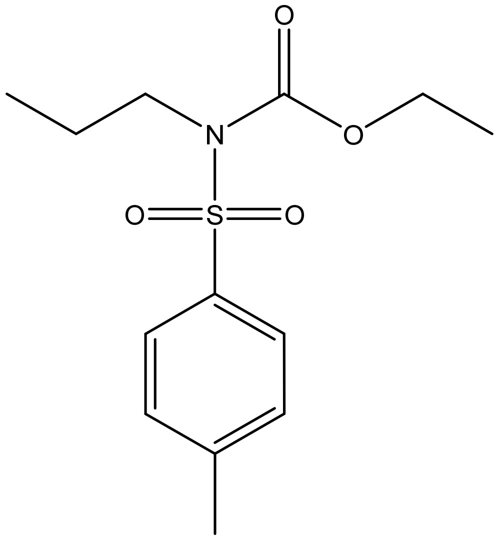

Prepared from unrecrystallised N-n-propyl-4-toluenesulphonamide, **30k**, (6.51 g, 30.5 mmol) and sodium hydride (60% dispersion in mineral oil, 1.80 g, 45.0 mmol) and ethyl chloroformate (3.83 g, 35.3 mmol) in the manner described in illustrative

procedure 5 but with a larger volume of tetrahydrofuran (250 ml). A gelatinous precipitate which impeded string formed in the flask, but addition of the chloroformate dispersed this material and led to efficient string. A pale yellow oil (9.05 g, 104%) was obtained, which contained residual ethyl chloroformate, but which slowly solidified on standing for several weeks. Recrystallisation from ethanol/water gave white needles (7.43 g, 85%), mp 43-44 °C.

HRMS (ESI) *m/z* calcd for $C_{13}H_{20}O_4NS^+$: 286.11076 $[M+H]^+$; found: 286.11111.

26ciii*

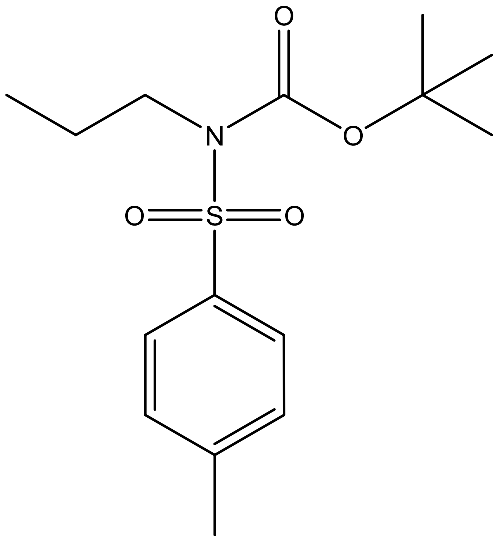

Prepared from unrecrystallised N-n-propyl-4-toluenesulphonamide, **30k**, (6.40 g, 30.0 mmol), di-t-butyldicarbonate (7.25, 33.2 mmol) and 4-dimethylaminopyridine (0.25 g, 2.0 mmol) in the manner described in illustrative procedure 6. An almost colourless oil (9.35 g, 99%) was obtained, which solidified on standing for several days; recrystallisation from ethanol/water gave white needles (6.25 g, 66%), mp 45-46 °C.

HRMS (ESI) *m/z* calcd for $C_{15}H_{24}O_4NS^+$: 314.14206 $[M+H]^+$; found: 314.14258.

26di*

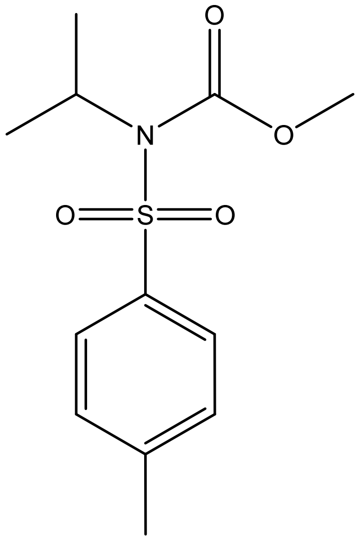

Prepared from N-isopropyl-4-toluenesulphonamide, **30o**, (5.75 g, 27.0 mmol) and sodium hydride (50% dispersion in mineral oil, 2.9 g, 60.4 mmol) and methyl chloroformate (3.0 g, 31.7 mmol) in the manner described in illustrative procedure 4, but with a larger volume of tetrahydrofuran (total of 450 ml). The usual extractive workup gave a colourless oil (7.26 g, 99%), which slowly solidified to a creamy white solid on standing for two days; recrystallisation from ethanol/water gave white needles (6.85 g, 94%), mp 154-6 °C.

HRMS (ESI) *m/z* calcd for $C_{12}H_{18}O_4NS^+$: 272.09511 $[M+H]^+$; found: 272.09503.

26dii*

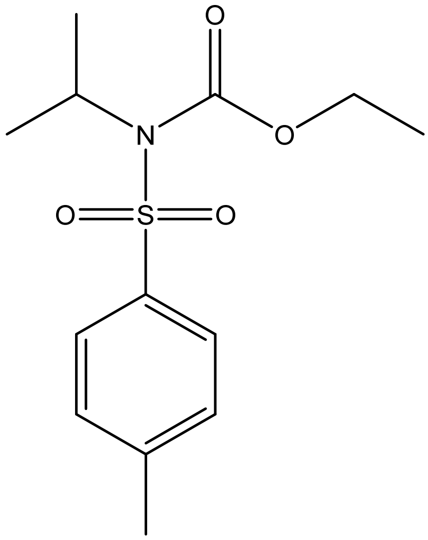

Prepared from N-isopropyl-4-toluenesulphonamide, **30o**, (6.11 g, 28.6 mmol) and sodium hydride (50% dispersion in mineral oil, 3.5 g, 61.5 mmol) and ethyl chloroformate (3.5 g, 32.3 mmol) in the manner described in illustrative procedure 5, but with a larger volume of tetrahydrofuran (total of 400 ml). The usual extractive workup gave a colourless oil (8.03 g, 103%), which solidified on standing overnight; recrystallisation from ethanol/water gave white needles (7.01 g, 90%), mp 44-5 °C, lit 114-5 °C.

HRMS (ESI) *m/z* calcd for $C_{13}H_{20}O_4NS^+$: 286.11076 $[M+H]^+$; found: 286.11130.

26diii

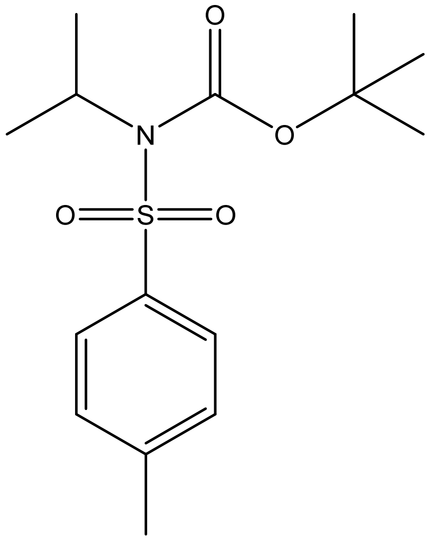

Prepared from N-isopropyl-4-toluenesulphonamide, **30o**, (6.51 g, 30.5 mmol), di-t-butyldicarbonate (8. 51, 37.4 mmol) and 4-dimethylaminopyridine (0.25 g, 2.0 mmol) in the manner described in illustrative procedure 6. A white solid (9.15 g, 96%) was obtained, which was recrystallised from ethanol/water to give white needles (7.95 g, 83%), mp 87-8 °C, lit 83-5 °C.

HRMS (ESI) *m/z* calcd for $C_{15}H_{24}O_4NS^+$: 314.14206 $[M+H]^+$; found: 314.14182.

26ei*

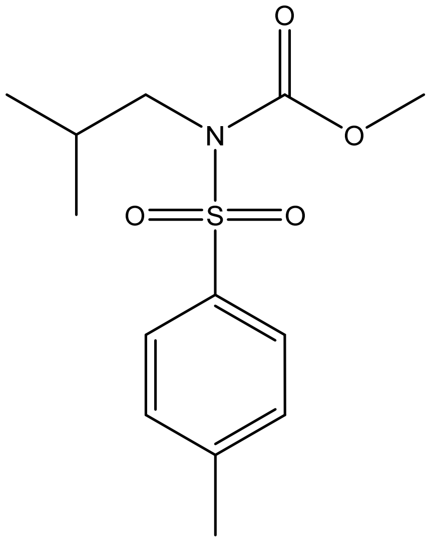

Prepared from unrecrystallised N-isobutyl-4-toluenesulphonamide, **30m**, (4.54 g, 20.0 mmol) and sodium hydride (60% dispersion in mineral oil, 2.0 g, 50.0 mmol) and methyl chloroformate (5.50 g, 58.2 mmol) in the manner described in illustrative procedure 4. After string overnight, tlc revealed only a trace of starting material. The usual extractive workup gave a pale yellow oil (5.76 g, 101%), which solidified on standing for two days. was obtained. Recrystallisation from ethanol/water gave white florets (4.35 g, 74%), mp 98-9 °C.

HRMS (ESI) *m/z* calcd for $C_{13}H_{20}O_4NS^+$: 286.11076 $[M+H]^+$; found: 286.11072.

26eii*

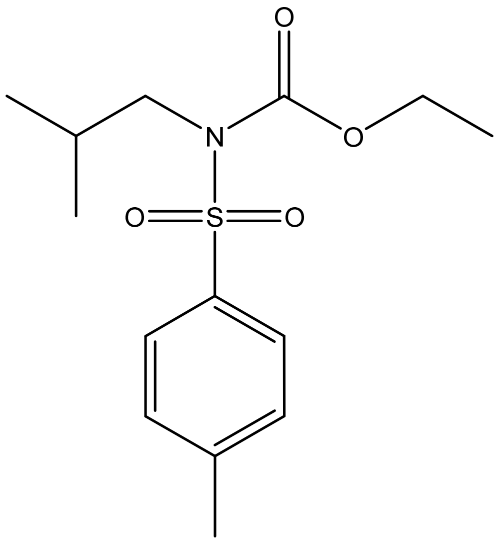

Prepared from unrecrystallised N-isobutyl-4-toluenesulphonamide, **30m**, (4.54 g, 20.0 mmol) and sodium hydride (60% dispersion in mineral oil, 1.9 g, 47.5 mmol) and ethyl chloroformate (5.0 g, 46.1 mmol) in the manner described in illustrative procedure 5. Despite using an greater volume of tetrahydrofuran (a total of 250 ml), difficulties were encountered with the formation of a gelatinous precipitate that impeded efficient string. After string for two days, tlc revealed only a trace of starting material. The usual extractive workup gave a pale yellow oil (6.19 g, 103%), which deposited large crystals (1.45 g, 35%) on standing for two further days;

recrystallisation of these crystals from ethanol/water gave white florets (1.45 g, 24%). The residual oil, which was found to contain a significant quantity of starting material, was treated a second time with sodium hydride (60% dispersion in mineral oil, 1.0 g, 25 mmol) and ethyl chloroformate (3.5 g, 32.2 mmol) in the manner described in illustrative procedure 5 to give a pale yellow oil (4.56 g), which solidified almost entirely on addition of a seed crystal from the recrystallised product that had previously been isolated. Recrystallisation from ethanol/water gave white florets (3.05 g, 51%), mp 46-8 °C. The overall yield of recrystallised product was 4.50 g, 75%. [The difficulties with preparing this derivative appear to reflect several factors, including the use of the last residue of an old sample of redistilled ethyl chloroformate.]

HRMS (ESI) *m/z* calcd for $C_{14}H_{22}O_4NS^+$: 300.12641 $[M+H]^+$; found: 300.12671.

26eiii*

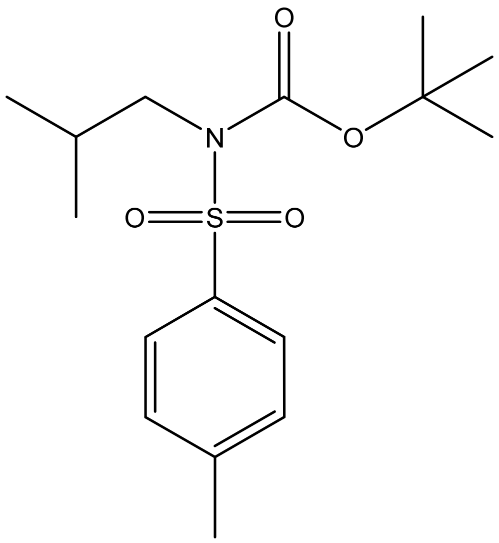

Prepared from unrecrystallised N-isobutyl-4-toluenesulphonamide, **30m**, (4.61 g, 20.3 mmol), di-t-butyldicarbonate (5.25, 24.1 mmol) and 4-dimethylaminopyridine (0.15 g, 1.2 mmol) in the manner described in illustrative procedure 6. A white solid

(6.41 g, 97%) was obtained, which was recrystallised from ethanol/water to give white florets (5.55 g, 84%), mp 81-3 °C.

HRMS (ESI) *m/z* calcd for $C_{16}H_{26}O_4NS^+$: 328.15771 [M+H]$^+$; found: 328.15811.

26fi*

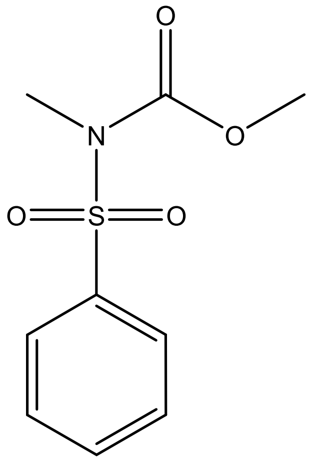

Prepared from N-methylbenzenesulphonamide, **30a**, (3.40 g, 19.9 mmol) in THF (50 mL) and sodium hydride (60% dispersion in mineral oil, 1.80 g, 45.0 mmol) in THF (50 mL) and methyl chloroformate (3.30 g, 34.9 mmol) in the manner described in illustrative procedure 4. The standard workup gave an almost colourless oil (4.25 g), which contained about 25% starting material; this mixture was dissolved in THF (60 mL) and treated with a second portion of NaH (0.95 g, 23.7 mmol) in THF 100 mL) and methyl chloroformate (2.25 g, 26.6 mmol). The product was obtained as a pale yellow oil (4.45 g, 97.7%), which could not be induced to solidify, but was pure by ^{1}H NMR.

HRMS (ESI) *m/z* calcd for $C_9H_{12}O_4NS^+$: 230.04816 [M+H]$^+$; found: 230.04767.

26fii*

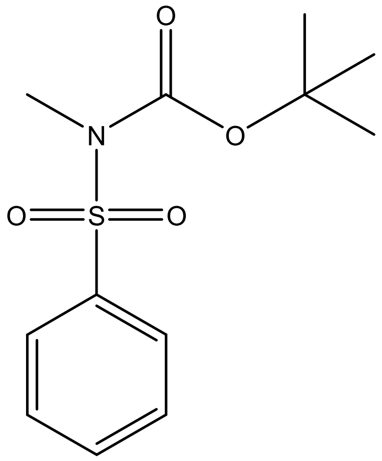

Prepared from N-methylbenzenesulphonamide, **30a**, (3.70 g, 21.6 mmol), di-t-butyldicarbonate (4.81, 22.1 mmol) and 4-dimethylaminopyridine (0.050 g, 0.41 mmol) in the manner described in illustrative procedure 6. A pale yellow oil (5.50 g, 93.7%) was obtained, which solidified on exposure to diaphragm pump pressure. Recrystallisation from ethanol/water gave white needles (4.54 g, 77.4%), mp 56-7 °C.

HRMS (ESI) *m/z* calcd for $C_{12}H_{18}O_4NS^+$: 272.09511 $[M+H]^+$; found: 272.09473.

26gi*

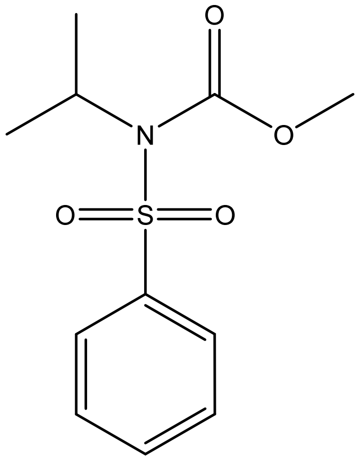

Prepared from N-isopropylbenzenesulphonamide, **30n**, (10.0 g, 50.2 mmol), and sodium hydride (50% dispersion in mineral oil, 4.6 g, 96 mmol) and methyl

chloroformate (6.30 g, 66.6 mmol) in the manner described in illustrative procedure 4. An almost colourless oil (12.5 g, 97%) was obtained.

HRMS (ESI) *m/z* calcd for $C_{11}H_{16}O_4NS^+$: 258.07946 [M+H]$^+$; found: 258.07938.

26gii*

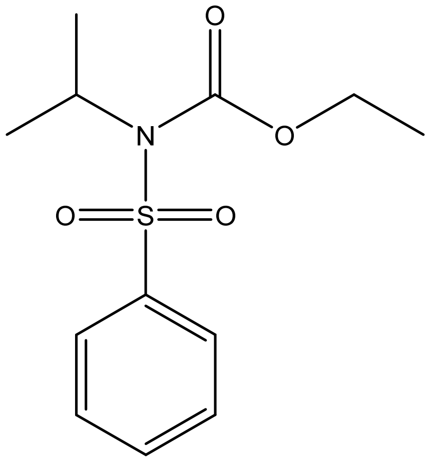

Prepared from N-isopropylbenzenesulphonamide, **30n**, (10.0 g, 50.2 mmol), and sodium hydride (50% dispersion in mineral oil, 5.2 g, 108 mmol) and ethyl chloroformate (7.00 g, 64.5 mmol) in the manner described in illustrative procedure 5. The usual extractive workup gave an almost colourless oil (13.5 g, 99%).

HRMS (ESI) *m/z* calcd for $C_{12}H_{18}O_4NS^+$: 272.09511 [M+H]$^+$; found: 272.09521.

26giii*

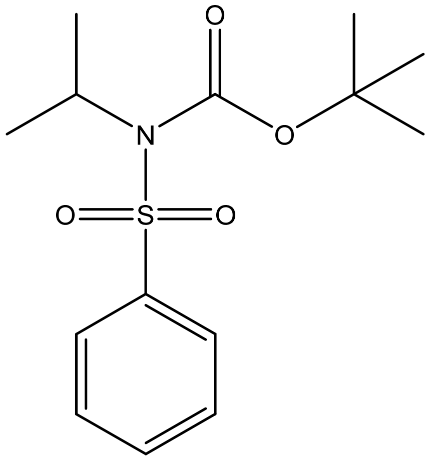

Prepared from N-isopropylbenzenesulphonamide, **30n**, (10.0 g, 50.2 mmol), di-t-butyldicarbonate (13.5, 61.1 mmol) and 4-dimethylaminopyridine (0.20 g, 1.6 mmol) in the manner described in illustrative procedure 6. A white solid (14.7g, 98%) was obtained, which was recrystallised from ethanol/water to give white florets (12.3 g, 82%), mp 60-1 °C.

HRMS (ESI) *m/z* calcd for $C_{14}H_{22}O_4NS^+$: 300.12641 $[M+H]^+$; found: 300.12637.

26hi*

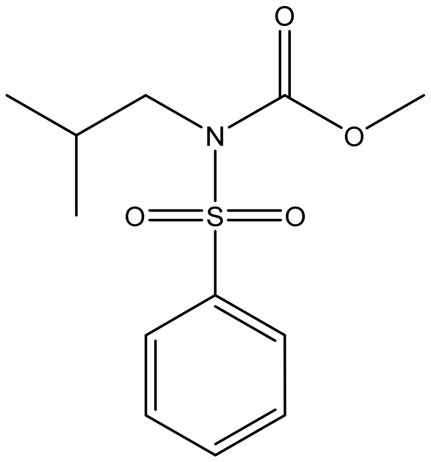

Prepared from N-isobutylbenzenesulphonamide, **30l**, (6.39 g, 30.0 mmol) and sodium hydride (60% dispersion in mineral oil, 2.0 g, 50.0 mmol) and methyl chloroformate (4.5 g, 47.6 mmol) in the manner described in illustrative procedure 4. A pale yellow oil (8.08 g, 99%), which slowly solidified over three days; recrystallisation from ethanol/water gave white needles (6.06 g, 74%), mp 51-2 °C.

HRMS (ESI) *m/z* calcd for $C_{12}H_{18}O_4NS^+$: 272.09511 $[M+H]^+$; found: 272.09518.

26hii*

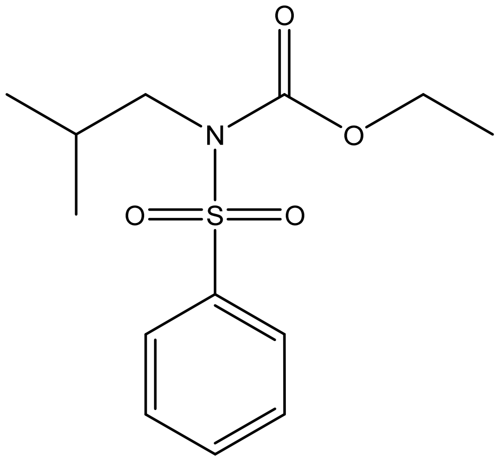

Prepared from N-isobutylbenzenesulphonamide, **30l**, (6.39 g, 30.0 mmol) and sodium hydride (60% dispersion in mineral oil, 2.0 g, 50.0 mmol) and ethyl chloroformate (4.5 g, 41.4 mmol) in the manner described in illustrative procedure 5. A pale yellow oil (8.50 g, 99%) was obtained.

HRMS (ESI) *m/z* calcd for $C_{13}H_{20}O_4NS^+$: 286.11076 $[M+H]^+$; found: 286.11093.

26hiii*

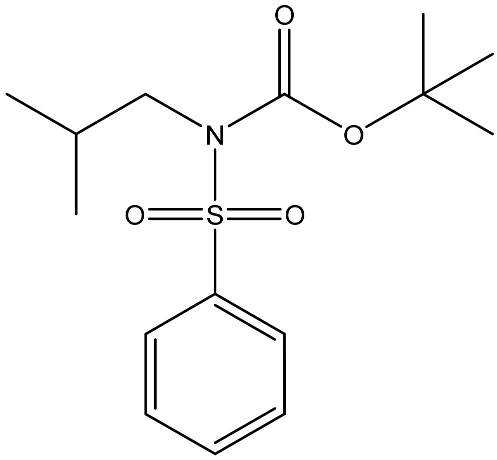

Prepared from N-isobutylbenzenesulphonamide, **30l**, (4.26 g, 20.0 mmol) di-t-butyldicarbonate (5.65, 25.9 mmol) and 4-dimethylaminopyridine (0.18 g, 1.5 mmol) in the manner described in illustrative procedure 6. A white solid (5.95 g, 95%) was

obtained, which was recrystallised from ethanol/water to give white florets (5.21 g, 83%), mp 88-9 °C.

HRMS (ESI) *m/z* calcd for $C_{13}H_{11}O_4NS^+$: 314.14206 [M+H]$^+$; found: 314.14175.

26ji*

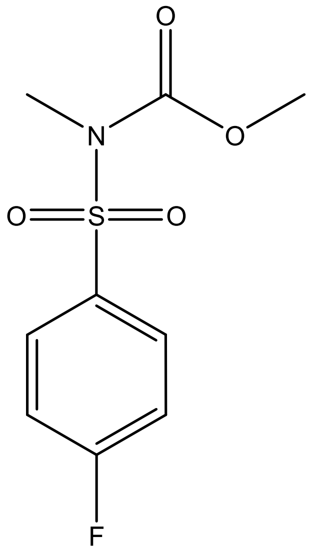

Prepared from N-methyl-4-fluorobenzenesulphonamide, **30c**, (3.78 g, 20.0 mmol) in THF (40 mL) and sodium hydride (60% dispersion in mineral oil, 1.20 g, 30.0 mmol) in THF (100 mL) and methyl chloroformate (3.00 g, 31.7 mmol) in the manner described in illustrative procedure 4. After stirring for two days, the standard workup gave an almost colourless oil (4.22 g), which contained about 30% starting material; this mixture was dissolved in THF (40 mL) and treated with a second portion of NaH (1.00 g, 25.0 mmol) in THF (50 mL) and methyl chloroformate (2.91 g, 30.7 mmol). The product was obtained as an almost colourless oil (4.93 g, 99.8%), which suddenly solidified; recrystallisation from ethanol/water gave white leaflets (3.71 g, 75.1%), mp 57-8 °C.

HRMS (ESI) *m/z* calcd for $C_9H_{11}O_4NSF^+$: 248.03873 [M+H]$^+$; found: 248.03844.

26jii*

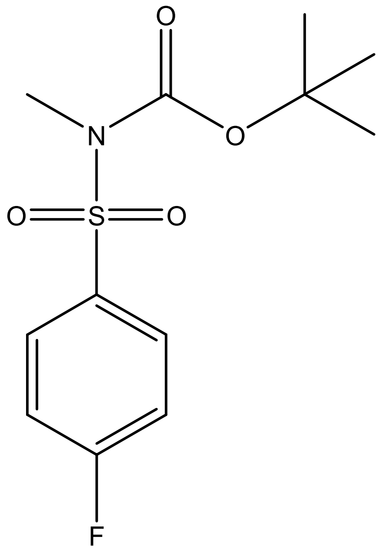

Prepared from N-methyl-4-fluorobenzenesulphonamide, **30c**, (3.78 g, 20.0 mmol), di-t-butyldicarbonate (4.81, 22.1 mmol) and 4-dimethylaminopyridine (0.060 g, 0.049 mmol) in the manner described in illustrative procedure 6. The product was a colourless oil (5.46 g, 94.5%), which solidified overnight. Recrystallisation from ethanol/water gave white needles (4.93 g, 85.3%), mp 59-60 °C.

HRMS (ESI) *m/z* calcd for $C_{12}H_{17}O_4NSF^+$: 290.08568 $[M+H]^+$; found: 290.08545.

26jiii*

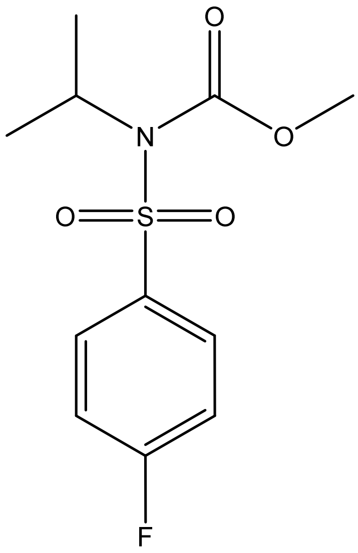

Prepared from N-methyl-4-fluorobenzenesulphonamide, **30p**, (7.90 g, 36.4 mmol) in THF (70 mL) and sodium hydride (60% dispersion in mineral oil, 3.90 g, 97.5 mmol) in THF (230 mL) and methyl chloroformate (4.65 g, 49.2 mmol) in the manner described in illustrative procedure 4. A gelatinous precipitate which impeded stirring formed in the flask, but addition of the chloroformate dispersed this material and led to efficient stirring. After stirring for a total of five days, tlc revealed no starting material. The product was obtained as a pale oil (9.68 g, 96.7%), which solidified on standing overnight. Recrystallisation from ethanol/water to gave white crystals, mp 54-5 °C (8.05 g, 80.4%).

HRMS (ESI) *m/z* calcd for $C_{11}H_{15}O_4NSF^+$: 276.07003 $[M+H]^+$; found: 276.07211.

26jiv*

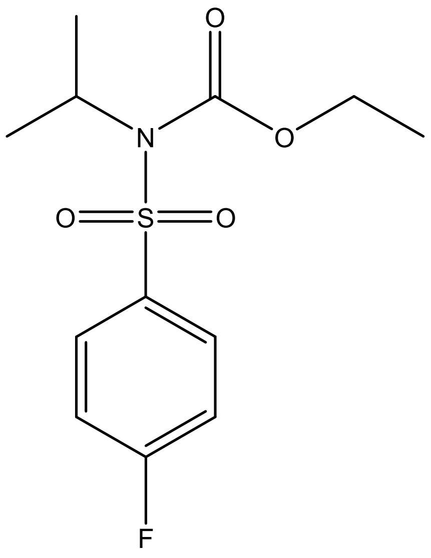

Prepared from N-isopropyl-4-fluorobenzenesulphonamide, **30p**, (4.30 g, 19.8 mmol) in THF (50 mL) and sodium hydride (60% dispersion in mineral oil, 1.20 g, 30.0 mmol) in THF (50 mL) and ethyl chloroformate (3.5 g, 37.0 mmol) in the manner described in illustrative procedure 5. A gelatinous precipitate which impeded stirring

formed in the flask, but addition of the chloroformate dispersed this material and led to efficient stirring. After stirring for a total of five days, tlc revealed mainly product. The standard workup gave a pale yellow oil (5.97 g), which 1H NMR revealed contained approximately 30% starting material. This mixture was dissolved in THF ((50 mL) and treated with a second portion of NaH (1.05 g, 26.2 mmol) in THF (50 mL) and ethyl chloroformate (1.80 g, 1.66 mmol). After stirring for to days, tlc showed no residual starting material. The standard workup gave a pale yellow oil (5.36 g, 93.5%), which could not be induced to solidify, but which was pure by ^{1}H NMR.

HRMS (ESI) *m/z* calcd for $C_{12}H_{17}O_4NSF^+$: 290.08568 $[M+H]^+$; found: 290.08554.

26jv*

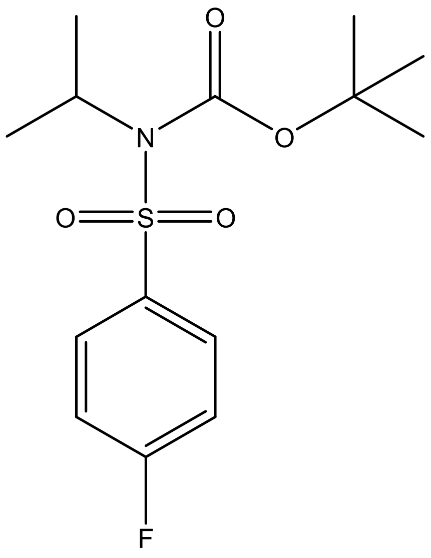

Prepared from N-isopropyl-4-fluorobenzenesulphonamide, **30p**, (6.80 g, 31.3 mmol), di-t-butyldicarbonate (8.00, 36.7 mmol) and 4-dimethylaminopyridine (0.20 g, 1.60 mmol) in the manner described in illustrative procedure 6. A pale peach coloured oil (10.35 g, 104%) was obtained, which contained residual solvent, but solidified

overnight to an almost white crystalline mass. Recrystallisation from ethanol/water gave white needles, 82-3 °C (8.10 g, 81.5%).

HRMS (ESI) *m/z* calcd for $C_{14}H_{21}O_4NSF^+$: 318.11698 [M+H]$^+$; found: 318.11679.

26ki*

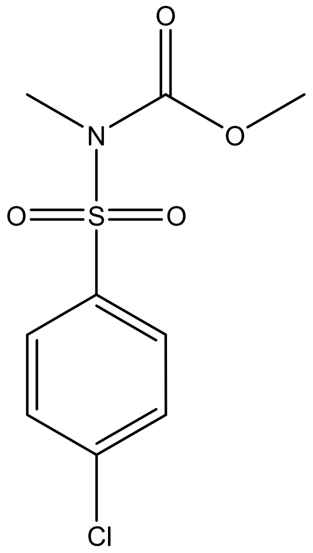

Prepared from N-methyl-4-chlorobenzenesulphonamide, **30k**, (4.11 g, 20.0 mmol) in THF (50 mL) and sodium hydride (60% dispersion in mineral oil, 1.80 g, 45.0 mmol) in THF (100 mL) and methyl chloroformate (4.00 g, 42.3 mmol) in the manner described in illustrative procedure 4. After stirring overnight, the standard workup gave an almost colourless oil (5.05 g), which contained about 30% starting material; this mixture was dissolved in THF (60 mL) and treated with a second portion of NaH (0.95 g, 23.7 mmol) in THF (100 mL) and methyl chloroformate (2.25 g, 23.8 mmol). The product was obtained as an almost colourless oil (4.98 g, 94.7%), which solidified on standing for two nights; recrystallisation from ethanol/water gave white needles (4.23 g, 80.2%), mp 62-3 °C.

HRMS (ESI) *m/z* calcd for $C_9H_{11}O_4NSCl^+$: 264.00918 [M+H]$^+$; found: 264.00903.

26kii*

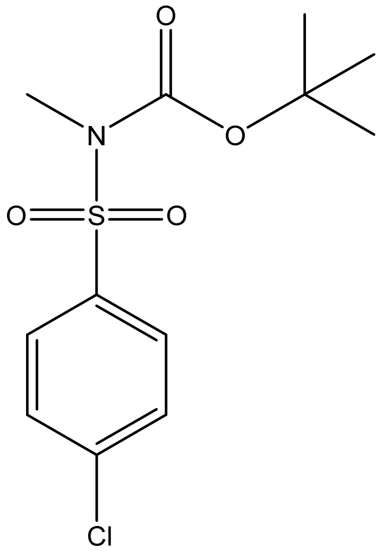

Prepared from N-methyl-4-chlorobenzenesulphonamide, **30k**, (4.11 g, 20.0 mmol), di-t-butyldicarbonate (4.65, 21.3 mmol) and 4-dimethylaminopyridine (0.048 g, 0.039 mmol) in the manner described in illustrative procedure 6. The product was a colourless oil (6.02 g, 98.5%), which quickly solidified. Recrystallisation from ethanol/water gave white needles (5.60 g, 91.7%), mp 99-100 °C.

HRMS (ESI) *m/z* calcd for $C_{12}H_{17}O_4NSCl^+$: 306.05613 $[M+H]^+$; found: 306.05502.

26kiii*

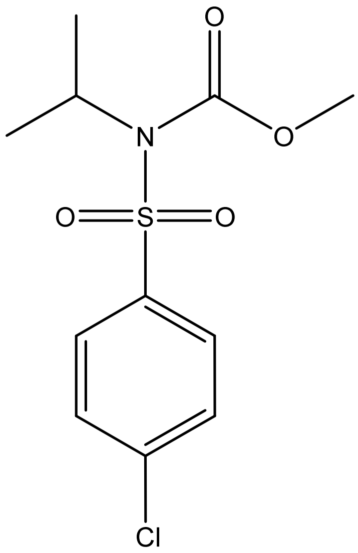

Prepared from N-isopropyl-4-chlorobenzenesulphonamide, **30q**, (5.60 g, 24.0 mmol) in THF (100 mL) and sodium hydride (50% dispersion in mineral oil, 2.80 g, 58.3 mmol) in THF (100 mL) and methyl chloroformate (4.30 g, 45.5 mmol) in the manner described in illustrative procedure 4. The product was obtained as a white solid (6.72 g, 96.1%), which was recrystallised from ethanol/water to gave white crystals (5.52 g, 79.3%) mp 75-6 °C.

HRMS (ESI) *m/z* calcd for $C_{11}H_{15}O_4NSCl^+$: 292.04048 $[M+H]^+$; found: 292.04056.

26kiv*

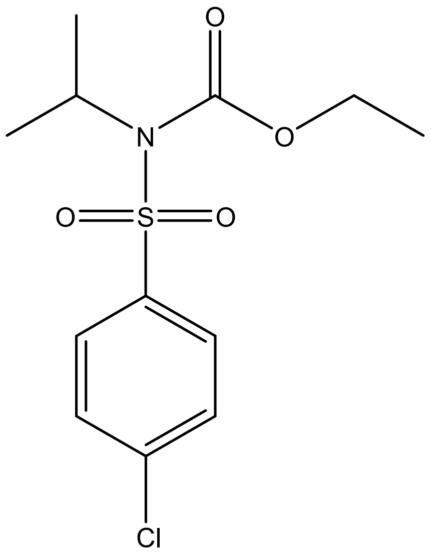

Prepared from N-isopropyl-4-chlorobenzenesulphonamide, **30q**, (5.50 g, 23.5 mmol) in THF (100 mL) and sodium hydride (50% dispersion in mineral oil, 1.60 g, 33.3 mmol) in THF (200 mL) and methyl chloroformate (4.30 g, 45.5 mmol) in the manner described in illustrative procedure 5. After stirring for a total of 3 h, tlc showed no starting material. The product was obtained as a pale yellow oil (7.08 g, 97.9%), which solidified on standing overnight. Recrystallisation from ethanol/water gave white crystals (5.83 g, 81.2%) mp 60-1 °C.

HRMS (ESI) *m/z* calcd for $C_{12}H_{17}O_4NSCl^+$: 306.05613 $[M+H]^+$; found: 306.05621.

26kv*

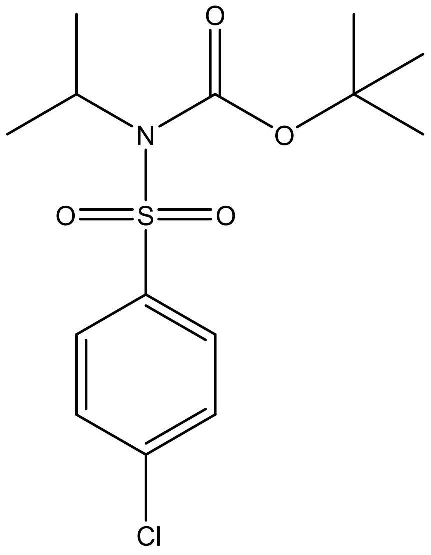

Prepared from N-isopropyl-4-chlorobenzenesulphonamide, **30q**, (5.60 g, 24.0 mmol), di-t-butyldicarbonate (12.2, 56.0 mmol) and 4-dimethylaminopyridine (0.35 g, 2.87 mmol) in the manner described in illustrative procedure 6. A white solid (8.01 g, 100.0%) was obtained. Recrystallisation from ethanol/water gave white needles (6.42 g, 80.0%) mp 96-7 °C.

HRMS (ESI) *m/z* calcd for $C_{14}H_{20}O_4NSClNa^+$: 356.06938 $[M+Na]^+$; found: 356.06915.

26li*

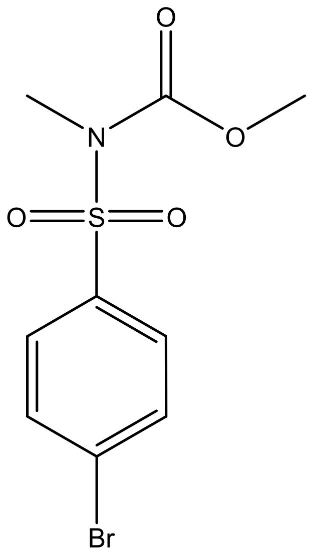

Prepared from N-methyl-4-bromobenzenesulphonamide, **30e**, (5.00 g, 20.0 mmol) in THF (50 mL) and sodium hydride (60% dispersion in mineral oil, 1.65 g, 41.2 mmol) in THF (100 mL) and methyl chloroformate (4.55 g, 48.1 mmol) in the manner described in illustrative procedure 4. After stirring for two nights, the standard workup gave an almost colourless oil (6.05 g), which contained about 25% starting material; this mixture was dissolved in THF (60 mL) and treated with a second portion of NaH (1.00 g, 25.0 mmol) in THF (100 mL) and methyl chloroformate (2.39 g, 25.3 mmol). The product was obtained as very pale oil (6.02 g, 97.7%), which could not be induced to solidify, but was pure by ^{1}H NMR.

HRMS (ESI) *m/z* calcd for $C_9H_{11}O_4NSBr^+$: 307.95867 $[M+H]^+$; found: 307.95367.

26lii*

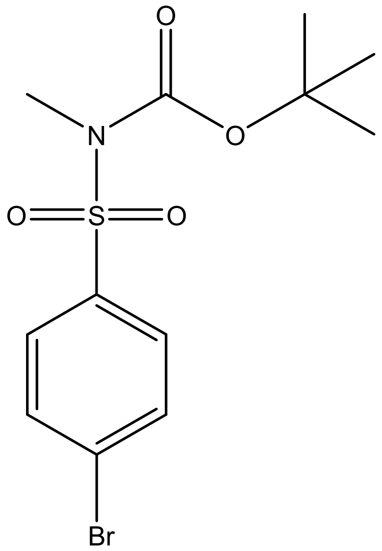

Prepared from N-methyl-4-bromobenzenesulphonamide, **30e**, (5.00 g, 20.0 mmol), di-t-butyldicarbonate (4.72, 21.6 mmol) and 4-dimethylaminopyridine (0.055 g, 0.045 mmol) in the manner described in illustrative procedure 6. The product was a colourless oil (6.90 g, 98.6%), which quickly solidified. Recrystallisation from ethanol/water gave white needles (6.57 g, 93.9%), mp 101-102 °C.

HRMS (ESI) *m/z* calcd for $C_{12}H_{17}O_4NSBr^+$: 350.00562 [M+H]$^+$; found: 350.00635.

26liii*

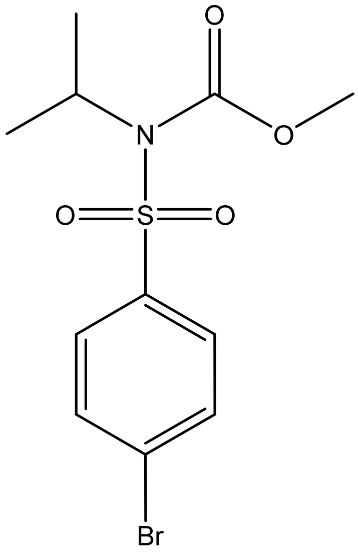

Prepared from N-isopropyl-4-bromobenzenesulphonamide, **30r**, (4.70 g, 16.9 mmol) in THF (100 mL) and sodium hydride (50% dispersion in mineral oil, 3.20 g, 66.6 mmol) in THF (100 mL) and methyl chloroformate (4.00 g, 42.3 mmol) in the manner described in illustrative procedure 4. The product was obtained as a white solid (5.62 g, 98.9%), which was recrystallised from ethanol/water to give white crystals (4.25 g, 75.1%) mp 66-7 °C.

HRMS (ESI) *m/z* calcd for $C_{11}H_{15}O_4NSBr^+$: 335.98997 [M+H]$^+$; found: 335.98978.

26liv*

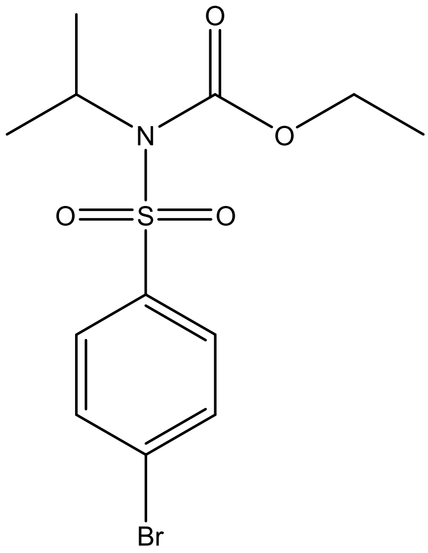

Prepared from N-isopropyl-4-bromobenzenesulphonamide, **30r**, (5.45 g, 19.6 mmol) in THF (100 mL) and sodium hydride (50% dispersion in mineral oil, 1.60 g, 33.3 mmol) in THF (200 mL) and ethyl chloroformate (3.00 g, 27.6 mmol) in the manner described in illustrative procedure 5. After stirring for only 45 min, tlc showed no starting material. The product was obtained as a pale yellow oil (6.93 g, 97.5%), which solidified on scratching. Recrystallisation from ethanol/water gave white crystals (5.57 g, 80.8%) mp 61-2 °C.

HRMS (ESI) *m/z* calcd for $C_{12}H_{17}O_4NSBr^+$: 350.00562 $[M+H]^+$; found: 350.00543.

26lv*

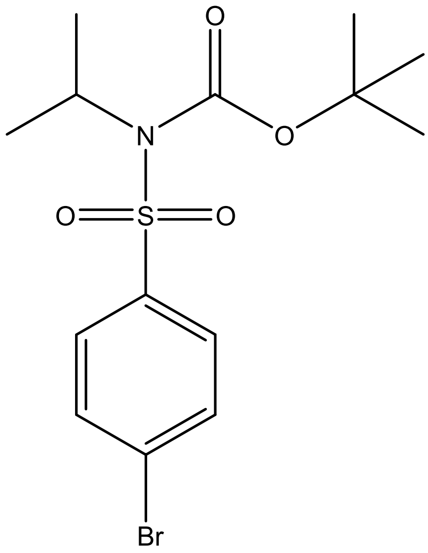

Prepared from N-isopropyl-4-bromobenzenesulphonamide, **30r**, (4.50 g, 16.2 mmol), di-t-butyldicarbonate (6.00, 27.5 mmol) and 4-dimethylaminopyridine (0.15 g, 1.23 mmol) in the manner described in illustrative procedure 6. A white solid (6.05 g, 99.0%) was obtained. Recrystallisation from ethanol/water gave white needles (4.85 g, 78.7%) mp 136-7 °C.

HRMS (ESI) *m/z* calcd for $C_{14}H_{20}O_4NSBrNa^+$: 400.01886 $[M+Na]^+$; found: 400.01889.

26mi*

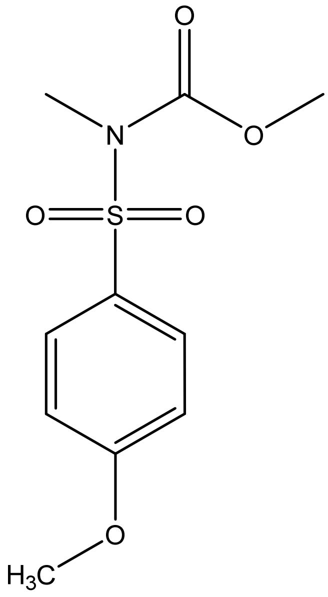

Prepared from N-methyl-4-methoxybenzenesulphonamide, **30f**, (4.02 g, 20.0 mmol) in THF (40 mL) and sodium hydride (60% dispersion in mineral oil, 1.00 g, 25.0 mmol) in THF (100 mL) and methyl chloroformate (3.00 g, 31.7 mmol) in the manner described in illustrative procedure 4. After stirring for two nights, the standard workup gave an almost colourless oil (4.85 g), which contained about 40% starting material; this mixture was dissolved in THF (40 mL) and treated with a second portion of NaH (1.00 g, 25.0 mmol) in THF (100 mL) and methyl chloroformate (2.62 g, 27.7 mmol). The product was obtained as very pale oil (5.05 g, 97.5%), which could not be induced to solidify, but was pure by ^{1}H NMR).

HRMS (ESI) *m/z* calcd for $C_{10}H_{14}O_5NS^+$: 260.05872 $[M+H]^+$; found: 260.05898.

26mii*

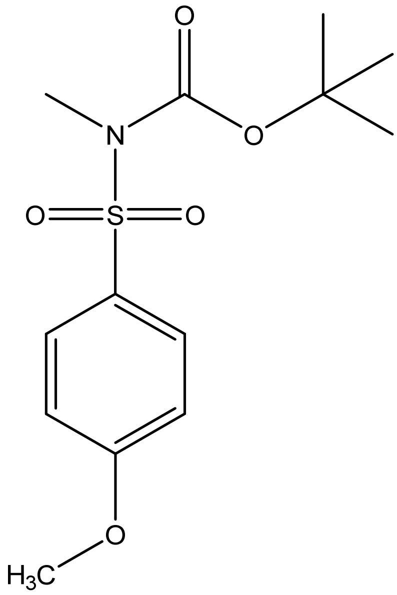

Prepared from N-methyl-4-methoxybenzenesulphonamide, **30f**, (4.02 g, 20.0 mmol), di-t-butyldicarbonate (4.65, 21.3 mmol) and 4-dimethylaminopyridine (0.105 g, 0.086 mmol) in the manner described in illustrative procedure 6. The product was a colourless oil (5.59 g, 90.6%). This material could not be induced to crystallise, but was pure by ^{1}H NMR.

HRMS (ESI) *m/z* calcd for $C_{13}H_{20}O_5NS^+$: 302.10567 $[M+H]^+$; found: 302.10635.

26miii*

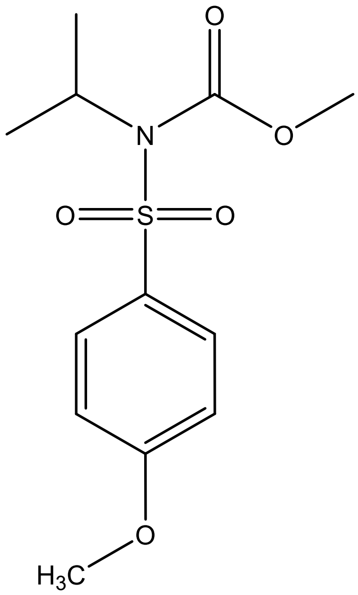

Prepared from N-isopropyl-4-methoxybenzenesulphonamide, **30s**, (5.34 g, 23.3 mmol) in THF (70 mL) and sodium hydride (60% dispersion in mineral oil, 2.50 g, 62.5 mmol) in THF (160 mL) and methyl chloroformate (3.57 g, 37.8 mmol) in the manner described in illustrative procedure 4. After stirring for 4 hours, tlc revealed only a trace of starting material. The standard workup gave a straw-coloured oil (6.27 g) which solidified on standing for 2 days. Recrystallisation from ethanol/water gave white crystals (4.35 g, 74.7%) mp 61-2 °C.

HRMS (ESI) *m/z* calcd for $C_{12}H_{18}O_5NS^+$: 288.09002 $[M+H]^+$; found: 288.09003.

26miv*

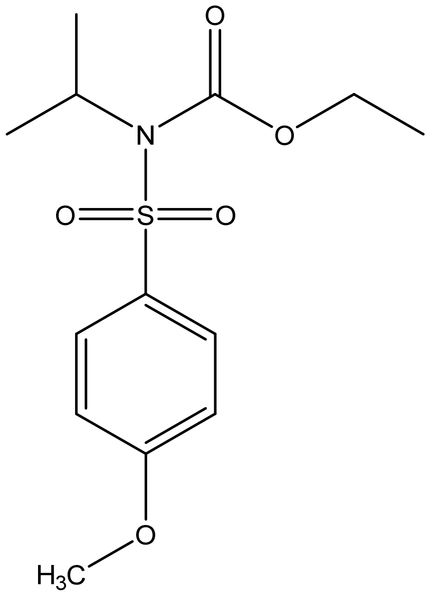

Prepared from N-isopropyl-4-methoxybenzenesulphonamide, **30s**, (5.32 g, 23.2 mmol) in THF (60 mL) and sodium hydride (60% dispersion in mineral oil, 3.60 g, 90.0 mmol) in THF (120 mL) and ethyl chloroformate (5.00 g, 52.9 mmol) in the manner described in illustrative procedure 5. After stirring for a total of 120 min, tlc revealed no starting material. The standard workup gave a pink oil (6.94 g, 98.2%), which could not be induced to solidify, but which ^{1}H NMR revealed to be pure.

HRMS (ESI) *m/z* calcd for $C_{13}H_{20}O_5NS^+$: 302.10567 $[M+H]^+$; found: 302.10550.

26mv*

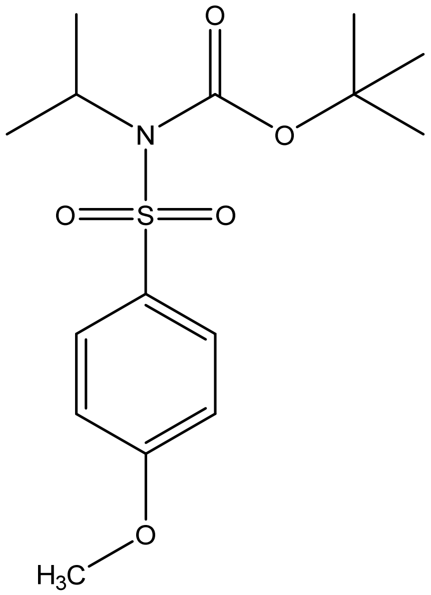

Prepared from N-isopropyl-4-methoxybenzenesulphonamide, **30s**, (7.85 g, 34.2 mmol), di-t-butyldicarbonate (8.15, 37.4 mmol) and 4-dimethylaminopyridine (0.25 g, 2.05 mmol) in the manner described in illustrative procedure 6. A pale yellow oil (11.25 g, 97.1%), which solidified on cooling to an almost white crystalline mass, was obtained. Recrystallisation from ethanol/water gave white needles (10.41 g, 89.8%) mp 74-5 °C.

HRMS (ESI) *m/z* calcd for $C_{15}H_{24}O_5NS^+$: 330.13697 $[M+H]^+$; found: 330.13687.

26ni*

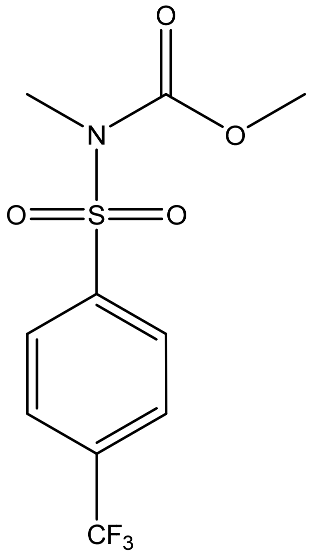

Prepared from N-methyl-4-trifluorobenzenesulphonamide, **30h**, (4.78 g, 20.0 mmol) in THF (50 mL) and sodium hydride (60% dispersion in mineral oil, 1.00 g, 25.0 mmol) in THF (100 mL) and methyl chloroformate (3.20 g, 33.8 mmol) in the manner described in illustrative procedure 4. A gelatinous precipitate formed after about half of the N-methyl-4-trifluorobenzenesulphonamide had been added, but it slowly dissolved as the methyl chloroformate was added. After stiring for a further 90 min, tlc showed no starting material (in contrast to the analogous reactions, which did not go to completion on the first treatment with NaH and methyl chloroformate). The standard workup gave a pale yellow oil (5.93 g, 99.8%), which solidified on standing over a weekend. Recrystallisation from ethanol/water gave white leaflets (4.55 g, 76.6%), mp 50-1 °C.

HRMS (ESI) *m/z* calcd for $C_{10}H_{11}O_4NSF_3^+$: 298.03554 $[M+H]^+$; found: 298.03574.

26nii*

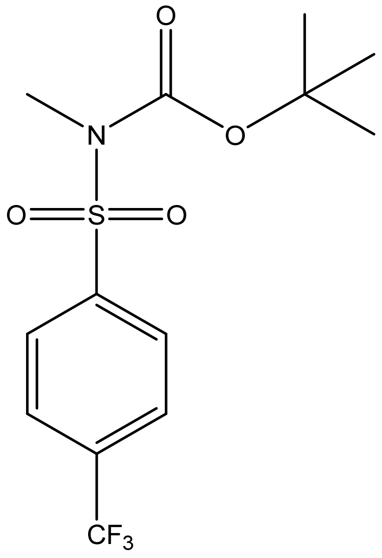

Prepared from N-methyl-4-trifluorobenzenesulphonamide, **30h**, (4.78 g, 20.0 mmol), di-t-butyldicarbonate (4.66, 21.3 mmol) and 4-dimethylaminopyridine (0.055 g, 0.045 mmol) in the manner described in illustrative procedure 6. The product was a crystalline solid (6.61 g, 97.5%). Recrystallisation from ethanol/water gave white needles (5.93 g, 87.5%), mp 66-7 °C.

HRMS (ESI) *m/z* calcd for $C_{13}H_{17}O_4NSF_3^+$: 340.08249 $[M+H]^+$; found: 340.08264.

26niii*

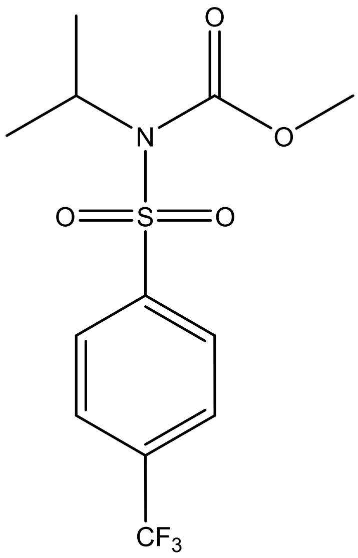

Prepared from N-isopropyl-4-trifluoromethylbenzenesulphonamide, **30u**, (5.47 g, 20.5 mmol) in THF (100 mL) and sodium hydride (60% dispersion in mineral oil, 3.20 g, 80.0 mmol) in THF (100 mL) and methyl chloroformate (76.95 g, 73.7 mmol) in the manner described in illustrative procedure 4. After stirring for a total of 9 days, tlc revealed mainly product, but some starting material remained. The standard workup gave a pale yellow oil (7.10 g), which ^{1}H NMR revealed contained approximately 25% starting material. This mixture was dissolved in THF (30 mL) and treated with a second portion of NaH (1.40 g, 35.0 mmol) in THF (40 mL) and methyl chloroformate (3.20 g, 33.9 mmol). After stirring for 30 min, tlc showed mainly product. The standard workup gave a yellow oil (7.25 g), which still contained approximately 5% starting material. This mixture was dissolved in THF (30 mL) and subjected to a third treatment with NaH (0.70 g, 17.5 mmol) in THF (40 mL) and methyl chloroformate (2.70 g, 28.5 mmol). After stirring for 3 h, tlc showed essentially only product. The standard workup gave a yellow oil (6.26 g, 94.0%). An aliquot of this oil was chromatographed on silica, eluting with ethyl acetate (25%) in petroleum ether (bp 40-60 °C fraction) to obtain a solid product, which acted as seed crystals to induce the main portion to solidify. Recrystallisation from ethanol/water gave cream crystals (7.25 g, 94.2%) mp 49-50 °C.

HRMS (ESI) *m/z* calcd for $C_{12}H_{15}O_4NSF_3^+$: 326.06684 [M+H]$^+$; found: 326.06696.

26niv*

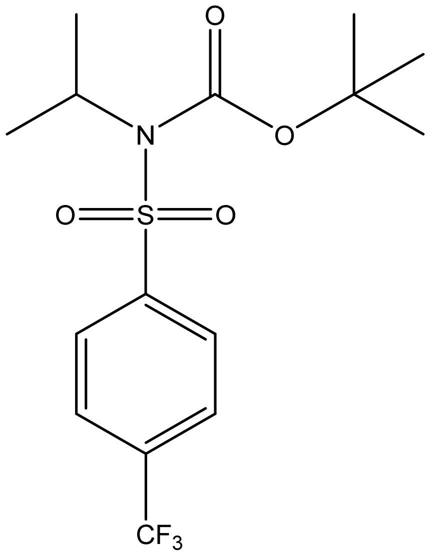

Prepared from N-isopropyl-4-trifluoromethylbenzenesulphonamide, **30u**, (5.71 g, 20.5 (7.85 g, 34.2 mmol), di-t-butyldicarbonate (8.15, 37.4 mmol) and 4-dimethylaminopyridine (0.25 g, 2.05 mmol) in the manner described in illustrative procedure 6. A pale yellow oil (11.25 g, 97.1%), which solidified on cooling to an almost white crystalline mass, was obtained. Recrystallisation from ethanol/water gave white needles (10.41 g, 89.8%) mp 131-2 °C.

26oi*

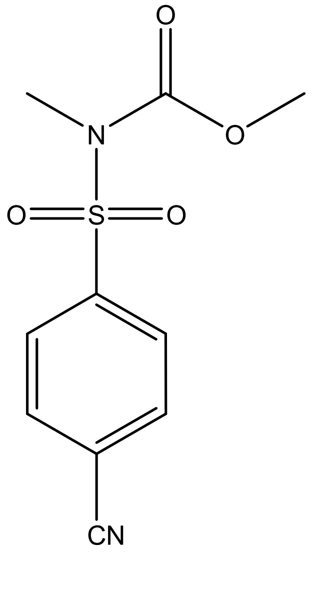

Prepared from N-methyl-4-cyanobenzenesulphonamide, **30g**, (3.92 g, 20.0 mmol) in THF (50 mL) and sodium hydride (60% dispersion in mineral oil, 1.00 g, 25.0 mmol) in THF (100 mL) and methyl chloroformate (3.15 g, 33.3 mmol) in the manner described in illustrative procedure 4. The reaction proceeded extraordinarily slowly, but was complete by tlc after a total of 13 days. The standard workup gave a pale cream solid (5.81 g, 114 %), which appeared from its 1H NMR spectrum to contain at most 1% starting material and traces of methyl chloroformate and THF. Recrystallisation from ethanol/water gave white needles (4.75 g, 93.5%), mp 101-2 °C.

HRMS (ESI) *m/z* calcd for $C_{10}H_{11}O_4N_2S^+$: 255.04340 $[M+H]^+$; found: 255.04419.

26oii*

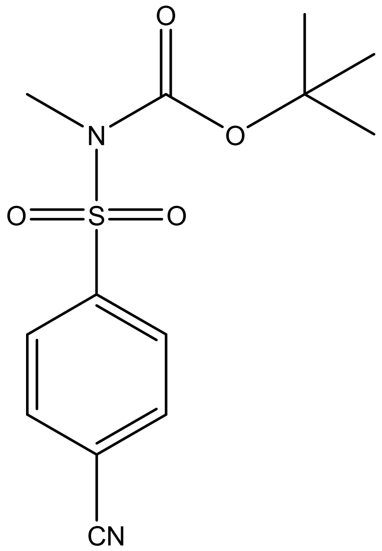

Prepared from N-methyl-4-cyanobenzenesulphonamide, **30g**, (3.92 g, 20.0 mmol), di-t-butyldicarbonate (4.77, 21.9 mmol) and 4-dimethylaminopyridine (0.057 g, 0.047 mmol) in the manner described in illustrative procedure 6. The product was a colourless oil (5.63 g, 95.1%), which solidified overnight. Recrystallisation from ethanol/water gave white leaflets (5.13 g, 86.7%), mp 134-5 °C.

HRMS (ESI) *m/z* calcd for $C_{13}H_{17}O_4N_2S^+$: 297.09035 $[M+H]^+$; found: 297.09039.

26oiii*

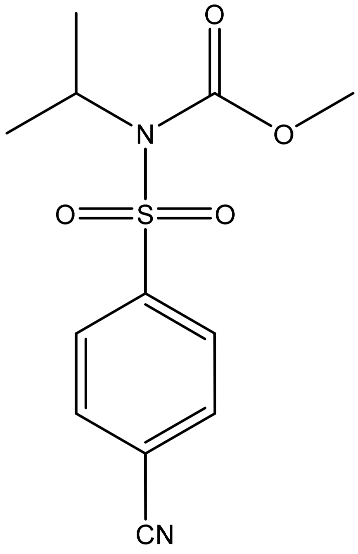

Prepared from N-isopropyl-4-cyanobenzenesulphonamide, **30t**, (6.17 g, 27.3 mmol) in THF (50 mL) and sodium hydride (60% dispersion in mineral oil, 2.05 g, 51.2 mmol) in THF (100 mL) and methyl chloroformate (3.10 g, 32.8 mmol) in the manner described in illustrative procedure 4. After stirring for five days, tlc revealed mainly product, but some starting material remained. The standard workup gave a pale yellow oil (8.25 g), which ^{1}H NMR revealed contained approximately 20% starting material. This mixture was dissolved in THF (40 mL) and treated with a second portion of NaH (1.40 g, 35.0 mmol) in THF (40 mL) and methyl chloroformate (3.45 g, 36.5 mmol). After stirring for 90 min, tlc showed no starting material. The standard workup gave a yellow oil (7.25 g), which solidified on standing overnight. Recrystallisation from ethanol/water gave white crystals (6.82 g, 75.2%) mp 81-2 °C.

HRMS (ESI) *m/z* calcd for $C_{12}H_{15}O_4N_2S^+$: 283.07470 $[M+H]^+$; found: 283.07709.

26oiv*

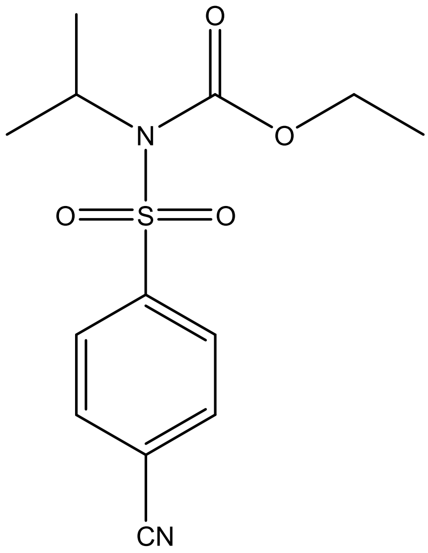

Prepared from N-isopropyl-4-cyanobenzenesulphonamide, **30t**, (6.72 g, 30.0 mmol) in THF (60 mL) and sodium hydride (60% dispersion in mineral oil, 2.05 g, 51.2 mmol) in THF (100 mL) and methyl chloroformate (7.68 g, 81.1 mmol) in the manner described in illustrative procedure 5. After stirring for 6 h, tlc revealed mainly product, but a trace of starting material remained. The standard workup gave a pale yellow oil (9.00 g). An aliquot of this oil was chromatographed on silica, eluting with ethyl acetate (25%) in petroleum ether (bp 40-60 °C fraction) to obtain a solid product, which acted as seed crystals to induce the main portion to solidify. Recrystallisation from ethanol/water gave white rhombs (6.78 g, 76.3%) mp 85-6 °C.

HRMS (ESI) *m/z* calcd for $C_{13}H_{16}O_4N_2SNa^+$: 319.07230 $[M+Na]^+$; found: 319.07227.

26ov*

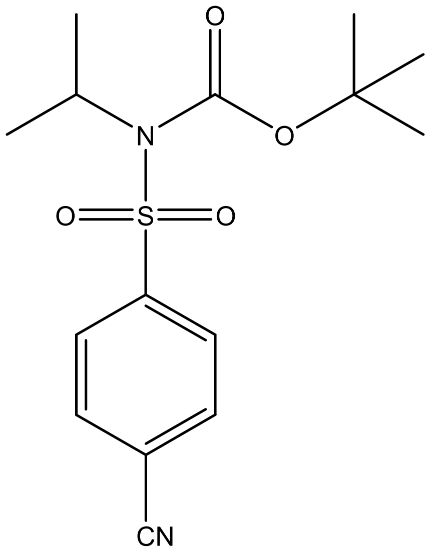

Prepared from N-isopropyl-4-cyanobenzenesulphonamide, **30t**, (3.36 g, 15.0 mmol), di-t-butyldicarbonate (4.50, 20.6 mmol) and 4-dimethylaminopyridine (0.30 g, 2.46 mmol) in the manner described in illustrative procedure 6. A white solid (4.48 g, 92.1%) was obtained. Recrystallisation from ethanol/water gave white needles (3.93 g, 80.8%).

26pi*

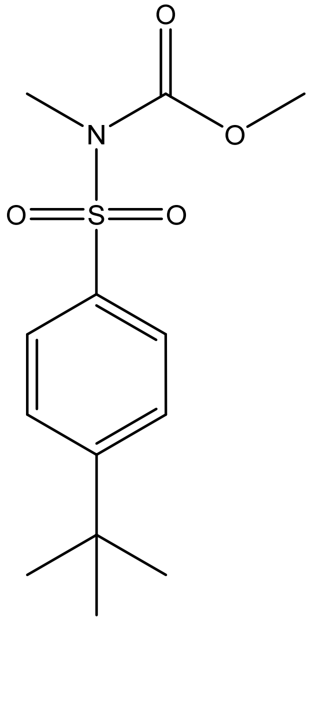

Prepared from N-methyl-4-t-butylbenzenesulphonamide, **30i**, (4.54 g, 20.0 mmol) in THF (50 mL) and sodium hydride (60% dispersion in mineral oil, 1.10 g, 27.5 mmol) in THF (100 mL) and methyl chloroformate (3.30 g, 34.9 mmol) in the manner described in illustrative procedure 4. After stirring for 3 h, the standard workup gave a pale yellow oil (5.55 g), which contained about 35% starting material; this mixture was dissolved in THF (40 mL) and treated with a second portion of NaH (1.00 g, 25.0 mmol) in THF (100 mL) and methyl chloroformate (3.05 g, 32.2 mmol). The product was obtained as very pale oil (5.65 g, 99.1%), which solidified on standing overnight. Recrystallisation from ethanol/water gave white crystals (4.04 g, 70.9%) mp 61-2 °C.

26pii*

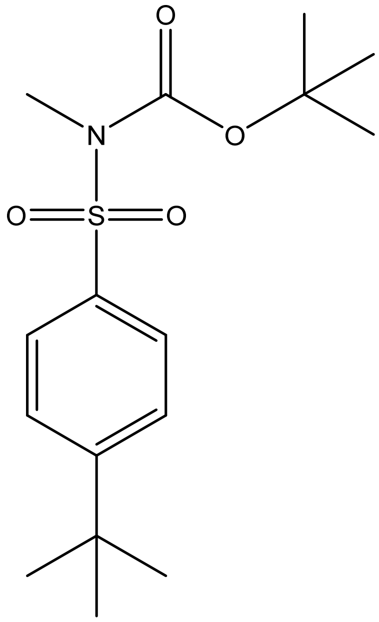

Prepared from N-methyl-4-t-butylbenzenesulphonamide, **30i**, (4.54 g, 20.0 mmol), di-t-butyldicarbonate (4.90, 22.5 mmol) and 4-dimethylaminopyridine (0.063 g, 0.052 mmol) in the manner described in illustrative procedure 6. The product was a colourless oil (6.23 g, 95.3%), which quickly solidified. Recrystallisation from ethanol/water gave white leaflets (6.04 g, 92.4%), mp 103-4 °C.

HRMS (ESI) *m/z* calcd for $C_{16}H_{26}O_4NS^+$: 328.15771 $[M+H]^+$; found: 328.15802.

26piii*

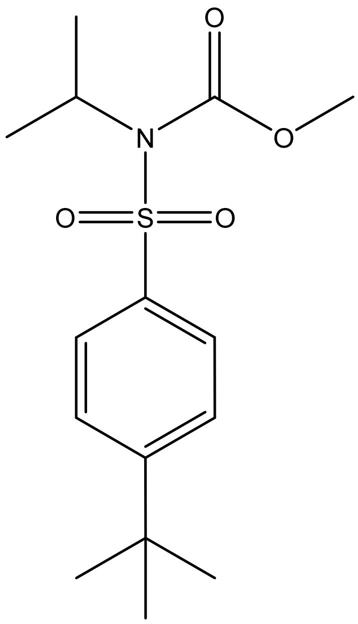

Prepared from N-isopropyl-4-t-butylbenzenesulphonamide, **30r**, (5.50 g, 21.5 mmol) in THF (30 mL) and sodium hydride (60% dispersion in mineral oil, 2.70 g, 67.5 mmol) in THF (80 mL) and methyl chloroformate (6.45 g, 68.3 mmol) in the manner described in illustrative procedure 4. After stirring for a total of 210 min, tlc revealed approximately 50% conversion to product. The standard workup gave a very pale yellow oil (6.03 g), which solidified on standing, but which ^1H NMR revealed contained approximately 40% starting material. This mixture was dissolved in THF (40 mL) and treated with a second portion of NaH (2.10 g, 52.5 mmol) in THF (50 mL) and methyl chloroformate (3.20 g, 33.9 mmol). After stirring for a total of 5 hr, tlc showed mainly product. The standard workup gave a very pale yellow oil (6.39 g), which still ^1H NMR revealed still contained approximately 12% starting material. This mixture was dissolved in THF (40 mL) and subjected to a third treatment with NaH (1.30 g, 32.5 mmol) in THF (100 mL) and methyl chloroformate (4.00 g, 42.3 mmol).

After stirring for 75 min, tlc showed essentially only product. The standard workup gave a pale oil (6.47 g, 95.8%), which was pure by ^{1}H NMR.

HRMS (ESI) *m/z* calcd for $C_{15}H_{24}O_4NS^+$: 314.14206 $[M+H]^+$; found: 314.14435.

26piv*

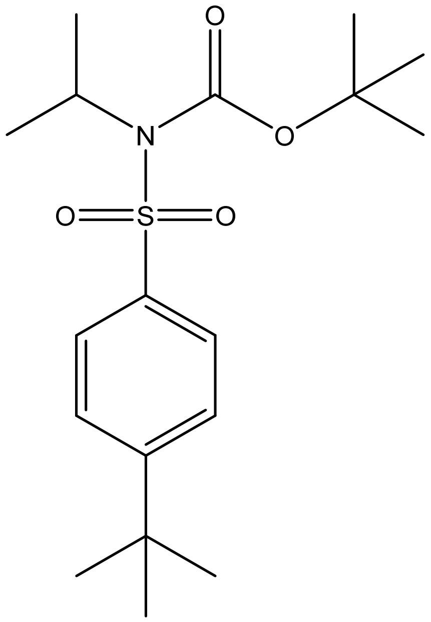

Prepared from N-isopropyl-4-t-butylbenzenesulphonamide, **30r**, (4.80 g, 18.8 mmol) di-t-butyldicarbonate (4.35, 20.0 mmol) and 4-dimethylaminopyridine (0.20 g, 1.64 mmol) in the manner described in illustrative procedure 6. A very pale yellow oil (6.85 g, 102%), containing traces of solvent, was obtained. This material solidified on cooling overnight in a freezer, Recrystallisation from ethanol/water gave white needles (5.73 g, 85.7%).

HRMS (ESI) *m/z* calcd for $C_{18}H_{30}O_4NS^+$: 356.18901 $[M+H]^+$; found: 356.18878.

4 Rearranged Sulphonamides

27ai*

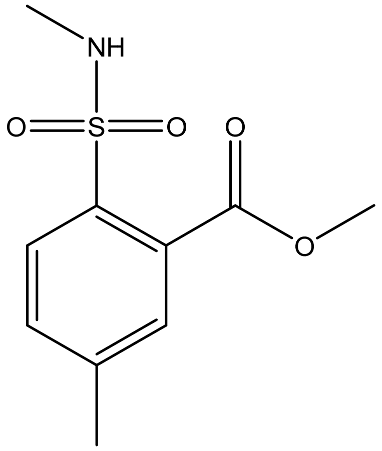

Illustrative procedure 7.

A fresh solution of lithium di-isopropylamide was prepared by adding di-isopropylamine (1.3 ml) and THF (4.7 ml) to a septum-sealed flame-dried round-bottomed flask that had been flushed with nitrogen. The flask was cooled to –78 °C in a dry-ice/acetone bath and the contents were magnetically stirred as n-butyllithium (4.0 ml, 2.5 M in hexane) was added.

In a separate flask, N-methylmethyl(tosyl)carbamate **26ai** ((1.217 g, 5.0 mmol)) was dissolved in THF (10 ml) and cooled to –78 °C. Lithium di-isopropylamine solution (1 M, 2 eq, 10 ml) was transferred to the magnetically stirred sulphonamide solution through a cannula by reducing the pressure. Two minutes after the transfer was complete, the reaction was quenched by addition of aqueous citric acid solution. After allowing the reaction mixture to attain ambient temperature, the product was extracted with dichloromethane, dried ($MgSO_4$), filtered and rotary evaporated to constant mass to give the crude product as a pale yellow oil (0.99 g, 81%). The

components were then separated by column chromatography on silica, eluting with petroleum ether (40 – 60 °C fraction) and ethyl acetate (2.5:1).The slower running component (the desired product) was obtained as a viscous oil (0.46 g, 45%).

HRMS (ESI) *m/z* calcd for $C_{10}H_{14}NO_4S^+$: 244.06370 [M+H]$^+$; found: 244.06375.

27aii*

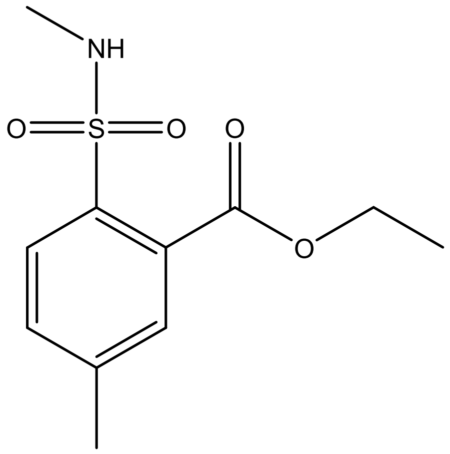

Prepared by treating **26aii** (1.292 g, 5.0 mmol) in THF (20 ml) with LDA (1 M, 2 eq, 10 ml) in the manner described in illustrative procedure 7. The crude product was obtained as cloudy yellow oil (1.13 g, 86%); the components were then separated by column chromatography on silica eluting with petroleum ether (40 – 60 °C fraction) and ethyl acetate (2.5:1). The slower running component was the desired product which was obtained as a pale yellow oil (0.64 g, 24%).

HRMS (ESI) *m/z* calcd for $C_{11}H_{16}NO_4S^+$: 258.07931 [M+H]$^+$; found: 258.07938.

27aiii*

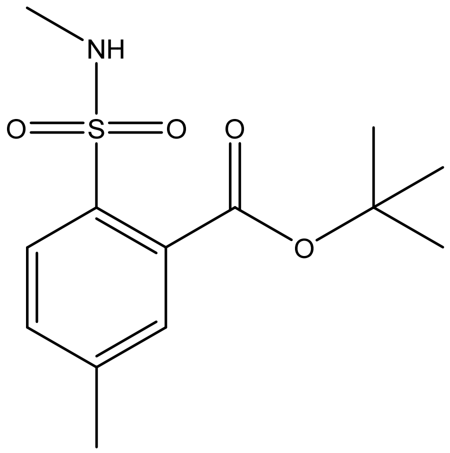

Prepared by treating **26aiii** (71.29 mg, 0.25 mmol) in THF (1 ml) with LDA (1 M, 2 eq, 0.5 ml) in the manner described in illustrative procedure 7. An orange oil (59 mg, 84%) was obtained.

HRMS (ESI) *m/z* calcd for $C_{13}H_{19}O_4NNaS^+$: 308.09270 $[M+Na]^+$; found: 308.09201.

27bi*

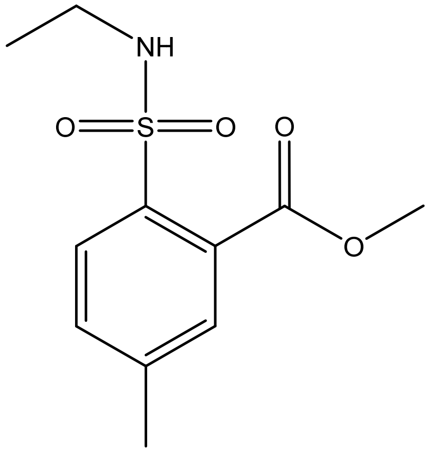

Prepared by treating **26bi** (0.65 g, 2.5 mmol) in THF (10 ml) with LDA (1 M, 2 eq, 5 ml) in the manner described in illustrative procedure 7. The crude product was obtained as orange oil (0.51 g, 79%); the components were then separated by column chromatography on silica eluting with petroleum ether (40 – 60 °C fraction) and ethyl acetate (2:1). The desired product which obtained as a colourless oil (0.33 g, 51%).

HRMS (ESI) *m/z* calcd for $C_{11}H_{16}NO_4S^+$: 258.07815 $[M+H]^+$; found: 258.07880.

27bii*

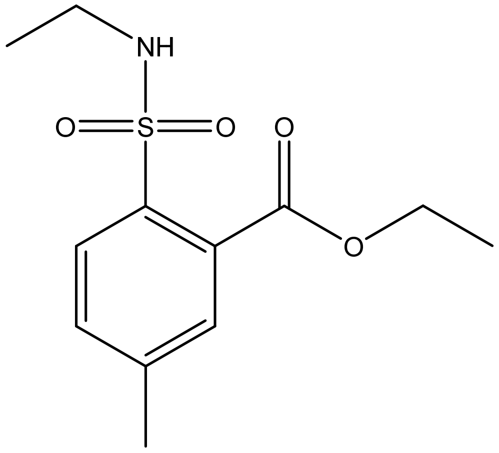

Prepared by treating **26bii** (0.679 g, 2.5 mmol) in THF (10 ml) with LDA (1 M, 2 eq, 5 ml) in the manner described in illustrative procedure 7. A yellow oil (73 %). was obtained. The crude product was obtained as orange oil (0.51g, 79 (0.49 g, 79%); the components were then separated by column chromatography on silica eluting with petroleum ether (40 – 60 °C fraction) and ethyl acetate (2:1). The desired product which obtained as a colourless oil (0.44 g, 64%).

HRMS (ESI) *m/z* calcd for $C_{12}H_{18}NO_4S^+$: 272.09344 $[M+H]^+$; found: 272.09427.

27biii*

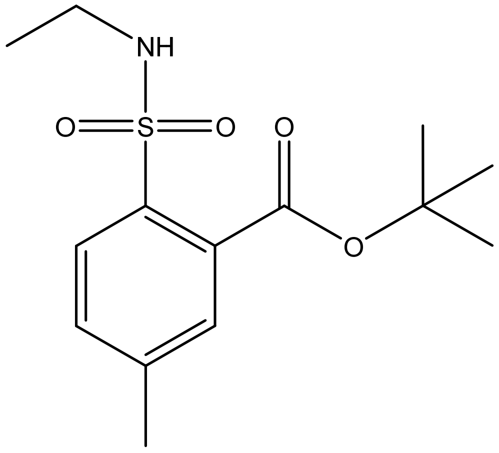

Prepared by treating **26biii** (78 mg, 0.25 mmol) in THF (1 ml) with LDA (1M, 2 eq, 0.5 ml) in the manner described in illustrative procedure 7. A viscous oil (67 mg, 86%) was obtained.

HRMS (ESI) *m/z* calcd for $C_{14}H_{22}O_4NS^+$: 300.12641 $[M+H]^+$; found: 300.12955.

27ci*

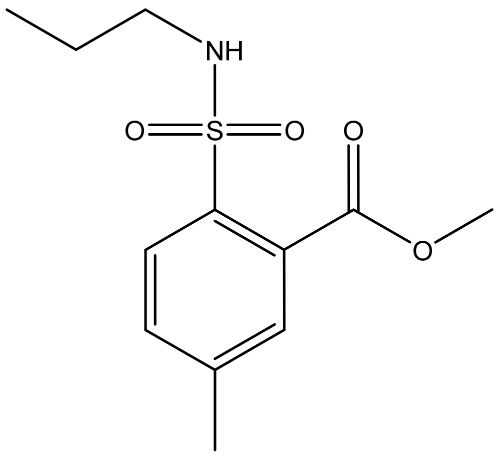

Prepared by treating **27ci** (1.36 g, 5.0 mmol) in THF (20 ml) with LDA (1 M, 2 eq, 10 ml) in the manner described in illustrative procedure 7. The crude product was obtained as pale yellow solid (0.95 g, 83%); the components were then separated by column chromatography on silica eluting with petroleum ether (40 – 60 °C fraction) and ethyl acetate (3:1). The desired product which obtained as white needles (0.44 g, 38%).

HRMS (ESI) *m/z* calcd for $C_{12}H_{18}O_4NS^+$: 272.09511 $[M+H]^+$; found: 272.09555.

27cii*

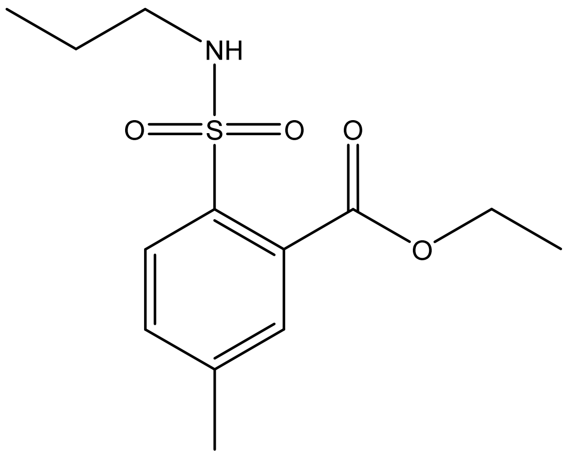

Prepared by treating **26cii** (71.5 mg, 0.25 mmol) in THF (1 ml) with LDA (1 M, 2 eq, 0.5 ml) in the manner described in illustrative procedure 7. A colourless oil (46.5 mg, 65%) was obtained.

HRMS (ESI) *m/z* calcd for $C_{13}H_{20}O_4NS^+$: 286.11076 [M+H]$^+$; found: 286.11132.

27ciii*

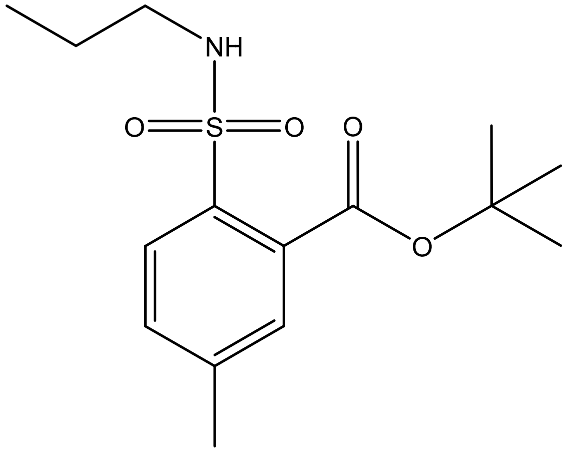

Prepared by treating **26ciii** (78.5 mg, 0.25 mmol) in THF (1 ml) with LDA (1 M, 2 eq, 0.5 ml) in the manner described in illustrative procedure 7. A pale yellow oil (71 mg, 91%) was obtained.

HRMS (ESI) *m/z* calcd for $C_{15}H_{23}O_4NS^+$: 336.12400 [M+H]$^+$; found: 336.12405.

27di*

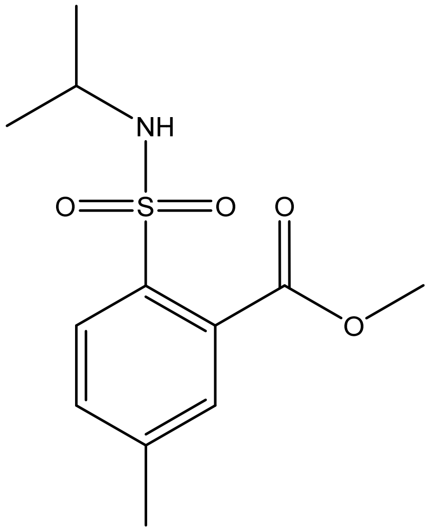

Prepared by treating **26di** (1.38 g, 5.0 mmol) in THF (20 ml) with LDA (1 M, 2 eq, 10 ml) in the manner described in illustrative procedure 7. A colourless oil (85%) which solidified on standing was obtained. Recrystallisation from ethanol/water gave white crystalline solid (0.94g, 68%); mp: 66 – 68 °C.

HRMS (ESI) *m/z* calcd for $C_{12}H_{18}NO_4S^+$: 272.09526 [M+H]$^+$; found: 272.09518.

27dii*

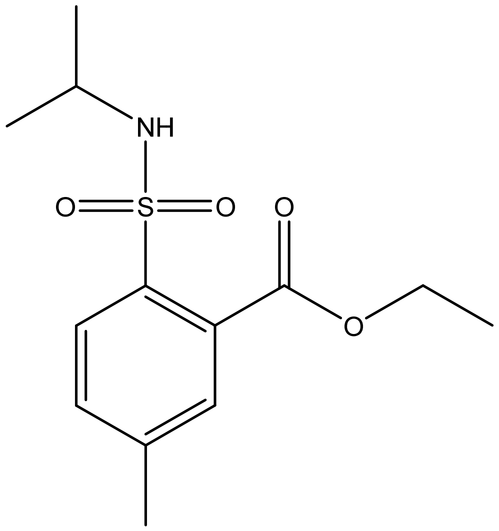

Prepared by treating **26dii** (1.43 g, 5.0 mmol) in THF (20 ml) with LDA (1 M, 2 eq, 10 ml) in the manner described in illustrative procedure 7. A white crystalline solid (1.15g, 78 %); mp: 92–93 °C was obtained.

HRMS (ESI) *m/z* calcd for $C_{13}H_{20}NO_4S^+$: 286.11117 [M+H]$^+$; found: 286.11096.

27diii*

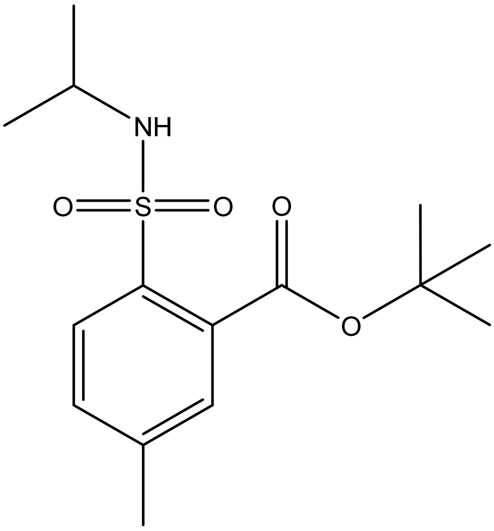

Prepared by treating **26diii** (1.58 g, 5.0 mmol) in THF (20 ml) with LDA (1 M, 2 eq, 10 ml) in the manner described in illustrative procedure 7. A colourless oil (1.31g, 83 %) was obtained.

HRMS (ESI) *m/z* calcd for $C_{15}H_{24}NO_4S^+$: 314.14225 [M+H]$^+$; found: 314.14215.

27ei*

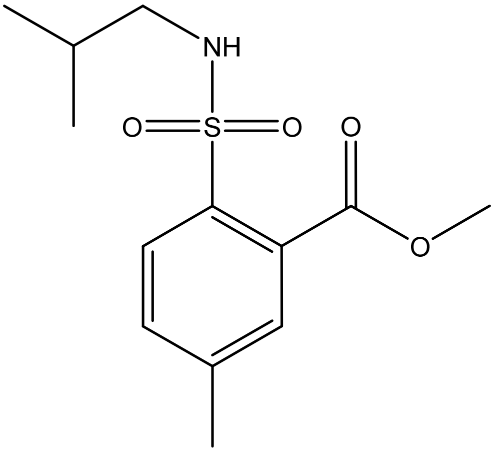

Prepared by treating **26ei** (0.713 g, 2.5 mmol) in THF (10 ml) with LDA (1 M, 2 eq, 5 ml) in the manner described in illustrative procedure 7. A colourless oil (0.59g, 83%) was obtained.

HRMS (ESI) *m/z* calcd for $C_{13}H_{20}O_4NS^+$: 286.11076 [M+H]$^+$; found: 286.11096.

27eii*

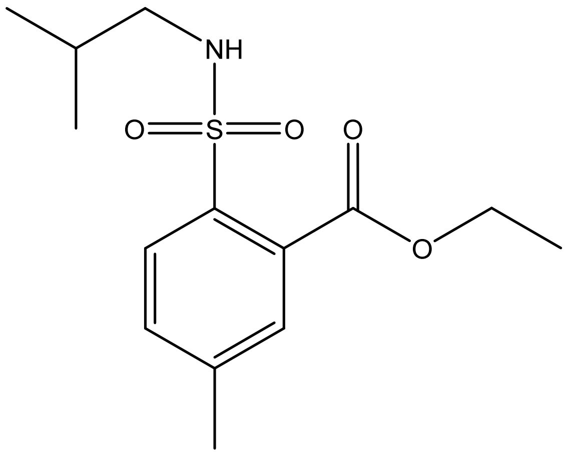

Prepared by treating **26eii** (74.8 mg, 0.25 mmol) in THF (1 ml) with LDA (1 M, 2 eq, 0.5 ml) in the manner described in illustrative procedure 7. A yellow oil (66.5 mg, 89%) was obtained.

HRMS (ESI) *m/z* calcd for $C_{14}H_{22}O_4NS^+$: 300.12641 $[M+H]^+$; found: 300.12650.

27eiii*

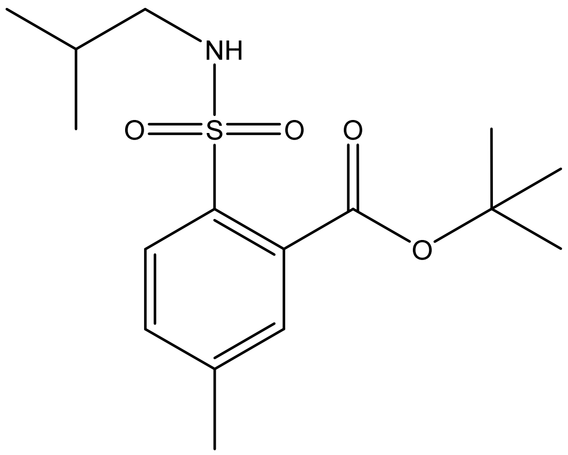

Prepared by treating **26eiii** (81.9 mg, 0.25 mmol) in THF (1 ml) with LDA (1 M, 2 eq, 0.5 ml) in the manner described in illustrative procedure 7. A pale orange oil (59 mg, 72%) was obtained.

27fi*

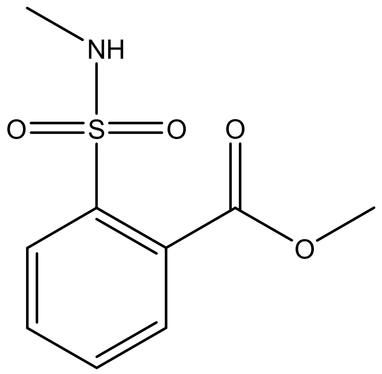

Prepared by treating **26fi** (0.57 g, 2.5 mmol) in THF (10 ml) with LDA (1 M, 2 eq, 5 ml) in the manner described in illustrative procedure 7. The crude product was obtained as pale yellow solid (0.51g, 79 (0.46 g, 80%); the components were then separated by column chromatography on silica eluting with petroleum ether (40 – 60 °C fraction) and ethyl acetate (2.5:1). The desired product which obtained as white needles (0.33 g, 58%).

HRMS (ESI) *m/z* calcd for $C_9H_{11}O_4NS^+$: 230.04816 $[M+H]^+$; found: 230.04637.

27fii*

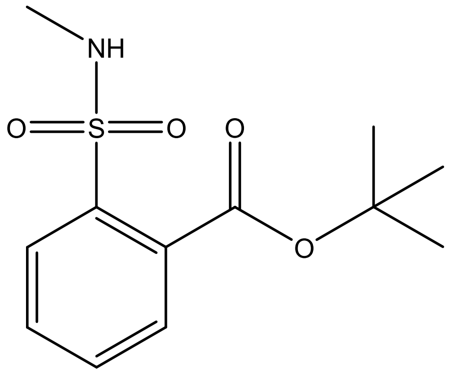

Prepared by treating **26fii** (0.679 g, 2.5 mmol) in THF (10 ml) with LDA (1 M, 2 eq, 5 ml) in the manner described in illustrative procedure 7. A yellow oil (73 %). was obtained. The crude product was obtained as orange oil (0.51g, 79 (0.49 g, 79%); the components were then separated by column chromatography on silica eluting with

petroleum ether (40 – 60 °C fraction) and ethyl acetate (2:1). The desired product which obtained as a colourless oil (0.44 g, 64%)

HRMS (ESI) *m/z* calcd for $C_{12}H_{18}O_4NS^+$: 272.09511 [M+H]$^+$; found: 272.09540.

27gi*

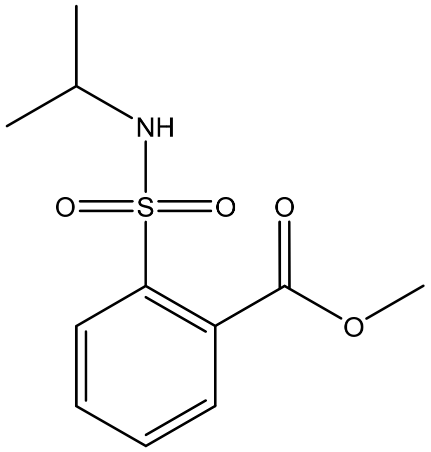

Prepared by treating **26gi** (1.29 g, 5.0 mmol) in THF (20 ml) with LDA (1 M, 2 eq, 10 ml) in the manner described in illustrative procedure 7. A white solid (0.99 g, 77%) was obtained. Recrystallisation from ethanol/water gave white rhombs (0.71 g, 55%), mp 58-9 °C.

HRMS (ESI) *m/z* calcd for $C_{11}H_{16}O_4NS^+$: 258.07946 [M+H]$^+$; found: 258.07938.

27gii*

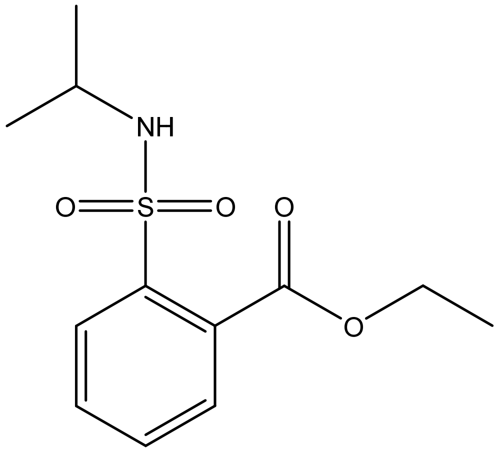

Prepared by treating **26gii** (0.678 g, 2.5 mmol) in THF (10 ml) with LDA (1 M, 2 eq, 5 ml) in the manner described in illustrative procedure 7. A white crystalline solid (0.64g, 95 %) was obtained. Recrystallisation from ethanol/water gave white needles (0.42 g, 62%), mp 54-5 °C.

HRMS (ESI) *m/z* calcd for $C_{12}H_{18}O_4NS^+$: 272.09511 $[M+H]^+$; found: 272.09506.

27giii

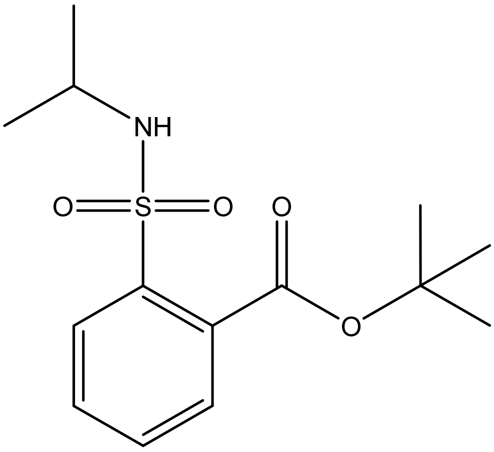

Prepared by treating **26giii** (0.749 g, 2.5 mmol) in THF (10 ml) with LDA (1 M, 2 eq, 5 ml) in the manner described in illustrative procedure 7. A white crystalline solid (0.73, 98%) was obtained. Recrystallisation from ethanol/water gave white iridescent leaflets (0.56 g, 75%), mp 126-7 C (lit 108-110 C).

HRMS (ESI) *m/z* calcd for $C_{14}H_{21}O_4NS^+$: 300.12641 $[M+H]^+$; found: 300.12662

27hi*

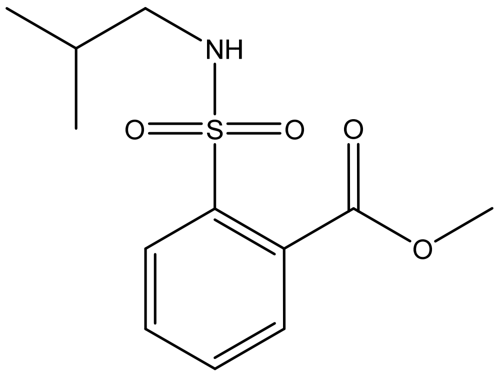

Prepared by treating **26hi** (0.678 g, 5.0 mmol) in THF (20 ml) with LDA (1 M, 2 eq, 10 ml) in the manner described in illustrative procedure 7. A yellow oil (0.61g, 90%) was obtained.

HRMS (ESI) *m/z* calcd for $C_{12}H_{18}O_4NS^+$: 272.09511 $[M+H]^+$; found: 272.09555.

27hii*

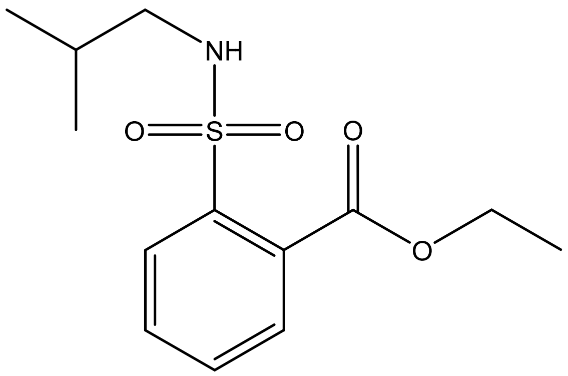

Prepared by treating **26hii** (71.4 mg, 0.25 mmol) in THF (1 ml) with LDA (1 M, 2 eq, 0.5 ml) in the manner described in illustrative procedure 7. A pale yellow oil (53.5 mg, 75%) was obtained.

HRMS (ESI) *m/z* calcd for $C_{13}H_{20}O_4NS^+$: 286.11076 $[M+H]^+$; found: 286.11108.

27hiii*

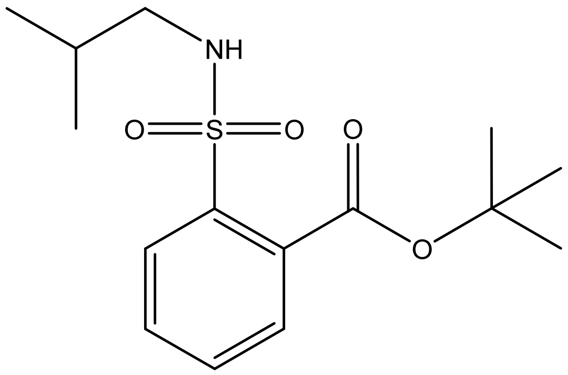

Prepared by treating **26hiii** (78.5 mg, 0.25 mmol) in THF (1 ml) with LDA (1 M, 2 eq, 0.5 ml) in the manner described in illustrative procedure 7. A pale yellow oil (64 mg, 80%) was obtained.

HRMS (ESI) *m/z* calcd for $C_{15}H_{24}O_4NS^+$: 314.14206 $[M+H]^+$; found: 314.14194.

27ji*

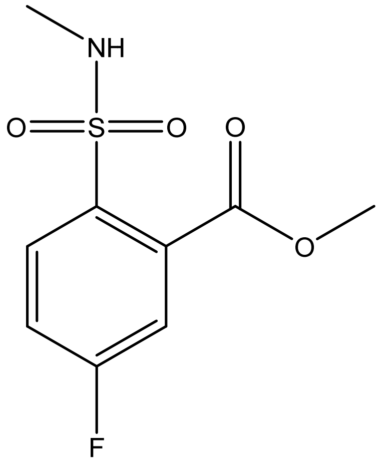

Prepared by treating **26ji** (0.6175 g, 2.5 mmol) in THF (10 ml) with LDA (1 M, 2 eq, 5 ml) in the manner described in illustrative procedure 7. The crude product was obtained as pale brown solid (0.55 g, 90%); the components were then separated by column chromatography on silica eluting with petroleum ether (40 – 60 °C fraction) and ethyl acetate (2:1). The desired product which obtained as white needles (0.358 g, 58%).

27jii*

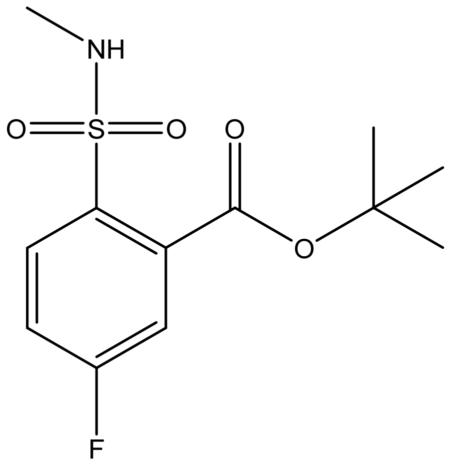

Prepared by treating **26jii** (0.7225 g, 2.5 mmol) in THF (10 ml) with LDA (1 M, 2 eq, 5 ml) in the manner described in illustrative procedure 7. The crude product was obtained as clear viscous oil (0.62 g, 86%).

HRMS (ESI) *m/z* calcd for $C_{12}H_{17}O_4NSF^+$: 290.08568 $[M+H]^+$; found: 290.08640.

27jiii*

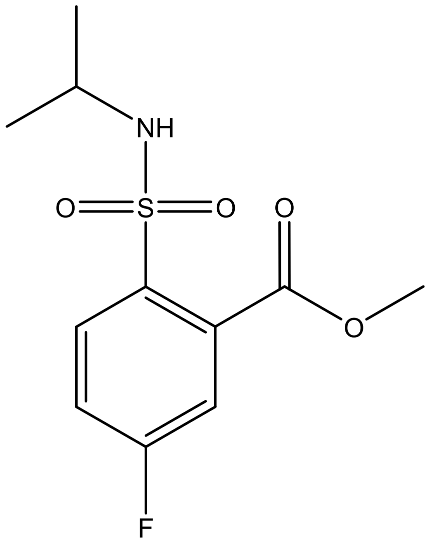

Prepared by treating **26jiii** (0.687g, 2.5 mmol) in THF (10 ml) with LDA (1 M, 2 eq, 5 ml) in the manner described in illustrative procedure 7. The crude product was obtained as pink solid (0.625 g, 91%). Recrystallisation from ethanol/water gave white needles (0.584 g, 85%), mp 66-7 °C.

HRMS (ESI) *m/z* calcd for $C_{11}H_{15}O_4NSF^+$: 276.07003 $[M+H]^+$; found: 276.06879.

27jiv*

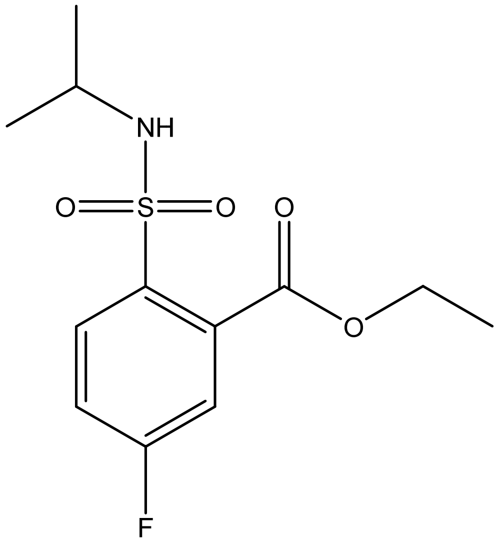

Prepared by treating **26jiv** (0.73 g, 2.5 mmol) in THF (10 ml) with LDA (1 M, 2 eq, 5 ml) in the manner described in illustrative procedure 7. The crude product was obtained as off-white solid (0.62 g, 91%). Recrystallisation from ethanol/water gave white iridescent leaflets (0.58 g, 85%).

HRMS (ESI) *m/z* calcd for $C_{12}H_{17}O_4NSF^+$: 290.08568 $[M+H]^+$; found: 290.08557.

27jv*

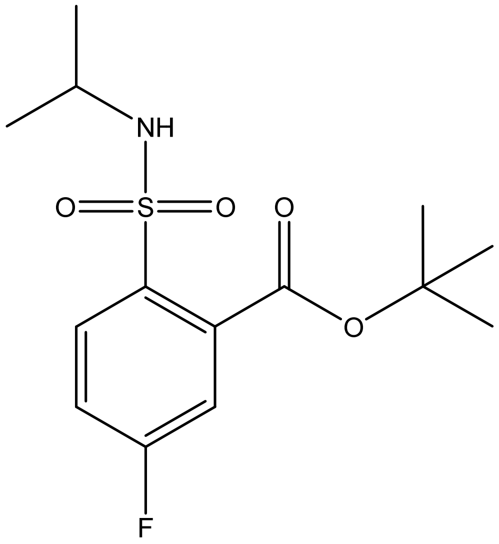

Prepared by treating **26jv** (0.79 g, 2.5 mmol) in THF (10 ml) with LDA (1 M, 2 eq, 5 ml) in the manner described in illustrative procedure 7. The crude product was obtained as pink solid (0.74 g, 91%). Recrystallisation from ethanol/water gave pink fluffy iridescent leaflets (0.66 g, 84%), mp 88-90 °C.

HRMS (ESI) *m/z* calcd for $C_{14}H_{21}O_4NSF^+$: 318.11698 $[M+H]^+$; found: 318.11694.

27ki*

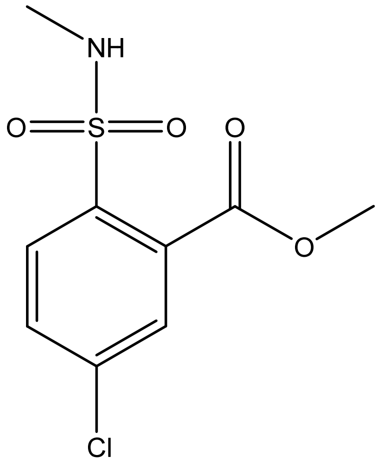

Prepared by treating **26ki** (0.6575 g, 2.5 mmol) in THF (10 ml) with LDA (1 M, 2 eq, 5 ml) in the manner described in illustrative procedure 7. The crude product was obtained as pink solid (0.65 g, 99%); the components were then separated by column chromatography on silica eluting with petroleum ether (40 – 60 °C fraction) and ethyl acetate (2.5:1). The desired product which obtained as white iridescent leaflets (0.50 g, 76%).

HRMS (ESI) *m/z* calcd for $C_9H_{11}O_4NSCl^+$: 264.00918 $[M+H]^+$; found: 264.00815.

27kii*

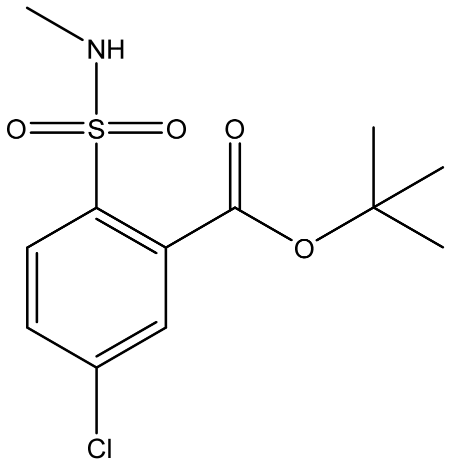

Prepared by treating **26kii** (0.7625 g, 2.5 mmol) in THF (10 ml) with LDA (1 M, 2 eq, 5 ml) in the manner described in illustrative procedure 7. The crude product was obtained as glass (0.71 g, 93%).

HRMS (ESI) *m/z* calcd for $C_{12}H_{17}O_4NSCl^+$: 306.05613 [M+H]$^+$; found: 306.05682.

27kiii*

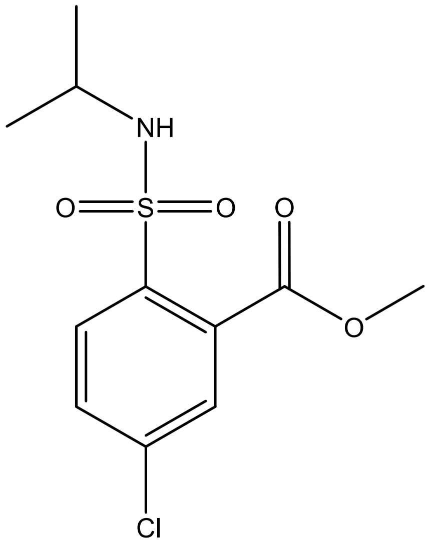

Prepared by treating **26kiii** (0.7275 g, 2.5 mmol) in THF (10 ml) with LDA (1 M, 2 eq, 5 ml) in the manner described in illustrative procedure 7. The crude product was obtained as pink solid (0.65 g, 89%). Recrystallisation from ethanol/water gave pink needles (0.56 g, 77%).

HRMS (ESI) *m/z* calcd for $C_{11}H_{15}O_4NSCl^+$: 292.04048 [M+H]$^+$; found: 292.04048.

27kiv*

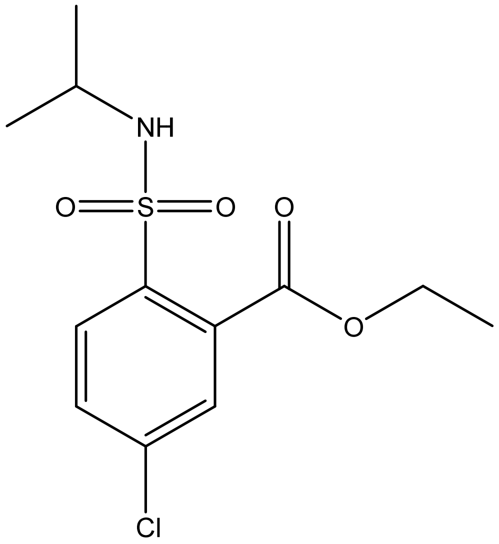

Prepared by treating **26kiv** (0.7625 g, 2.5 mmol) in THF (10 ml) with LDA (1 M, 2 eq, 5 ml) in the manner described in illustrative procedure 7. The crude product was obtained as pink solid (0.69 g, 91%). Recrystallisation from ethanol/water gave pink needles (0.66 g, 87%).

HRMS (ESI) *m/z* calcd for $C_{12}H_{17}O_4NSCl^+$: 306.05613 [M+H]$^+$; found: 306.05627.

27kv*

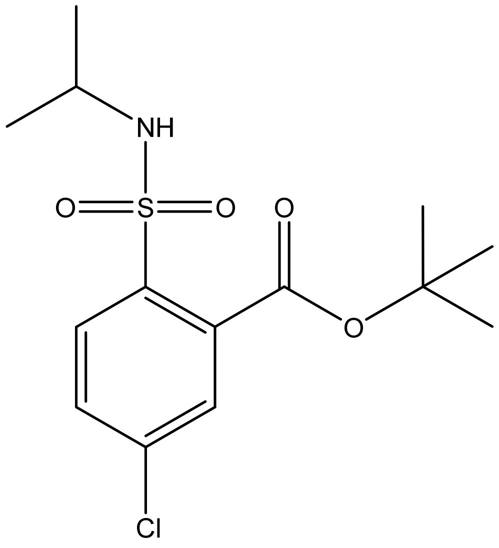

Prepared by treating **26kv** (0.8325 g, 2.5 mmol) in THF (10 ml) with LDA (1 M, 2 eq, 5 ml) in the manner described in illustrative procedure 7. The crude product was obtained as pale yellow solid (0.77 g, 93%). Recrystallisation from ethanol/water gave white iridescent leaflets (0.71 g, 85%), mp 89-90 °C.

HRMS (ESI) *m/z* calcd for $C_{14}H_{21}O_4NSCl^+$: 334.08743 $[M+H]^+$; found: 334.08740.

27li*

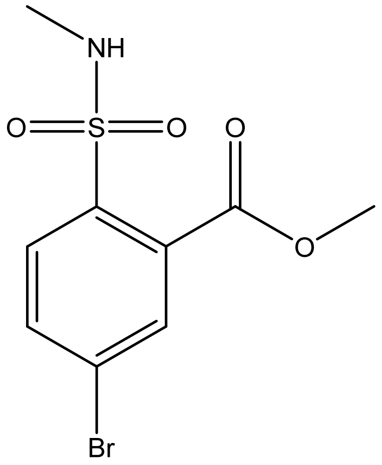

Prepared by treating **26li** (0.7675 g, 2.5 mmol) in THF (10 ml) with LDA (1 M, 2 eq, 5 ml) in the manner described in illustrative procedure 7. The crude product was obtained as pink solid (0.71 g, 92%); the components were then separated by column chromatography on silica eluting with petroleum ether (40 – 60 °C fraction) and ethyl acetate (2.5:1). The desired product which obtained as a white iridescent leaflets (0.64 g, 84%).

HRMS (ESI) *m/z* calcd for $C_9H_{11}O_4NSBr^+$: 307.95867 $[M+H]^+$; found: 307.95718.

27lii*

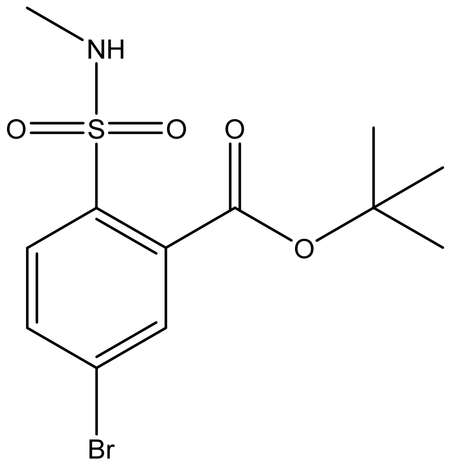

Prepared by treating **26lii** (0.8725 g, 2.5 mmol) in THF (10 ml) with LDA (1 M, 2 eq, 5 ml) in the manner described in illustrative procedure 7. The crude product was obtained as glass (0.85 g, 97%).

HRMS (ESI) *m/z* calcd for $C_{12}H_{17}O_4NSBr^+$: 350.00562 $[M+H]^+$; found: 350.00574.

27liii*

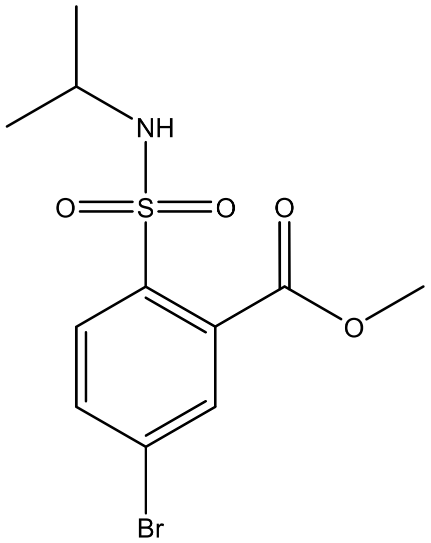

Prepared by treating **26liii** (0.840 g, 2.5 mmol) in THF (10 ml) with LDA (1 M, 2 eq, 5 ml) in the manner described in illustrative procedure 7. The crude product was obtained as pink semi solid (0.74 g, 88%); the components were then separated by column chromatography on silica eluting with petroleum ether (40 – 60 °C fraction)

and ethyl acetate (2:1). The desired product which obtained as white needles (0.52 g, 62%), mp 84-5 °C.

HRMS (ESI) *m/z* calcd for $C_{11}H_{15}O_4NSBr^+$: 335.98997 [M+H]$^+$; found: 335.99057.

27liv*

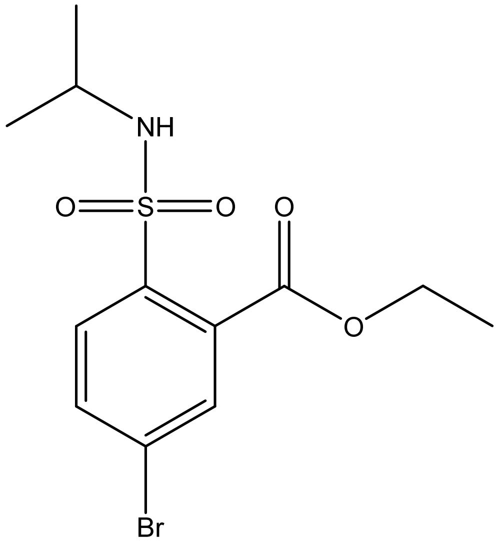

Prepared by treating **26liv** (0.875 g, 2.5 mmol) in THF (10 ml) with LDA (1 M, 2 eq, 5 ml) in the manner described in illustrative procedure 7. The crude product was obtained as pale yellow solid (0.86 g, 98%). Recrystallisation from ethanol/water gave white iridescent leaflets (0.76 g, 87%),

HRMS (ESI) *m/z* calcd for $C_{12}H_{17}O_4NSBr^+$: 350.00562 [M+H]$^+$; found: 350.00552.

27lv*

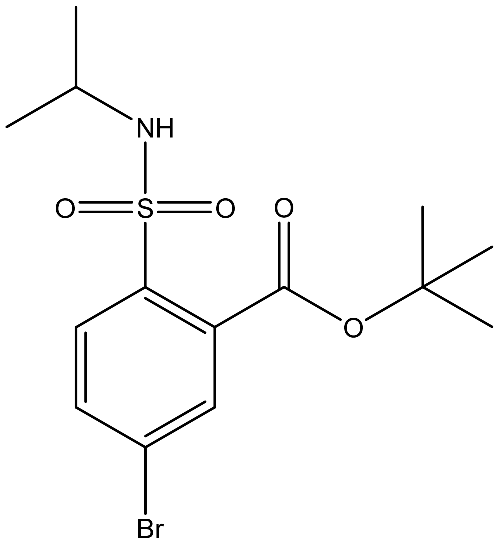

Prepared by treating **26lv** (0.945 g, 2.5 mmol) in THF (10 ml) with LDA (1 M, 2 eq, 5 ml) in the manner described in illustrative procedure 7. The crude product was obtained as white solid (0.87 g, 92%). Recrystallisation from ethanol/water gave white iridescent leaflets (0.793 g, 84%), mp 81-83 °C.

HRMS (ESI) *m/z* calcd for $C_{14}H_{21}O_4NSBr^+$: 378.03692 [M+H]$^+$; found: 378.03699.

27mi*

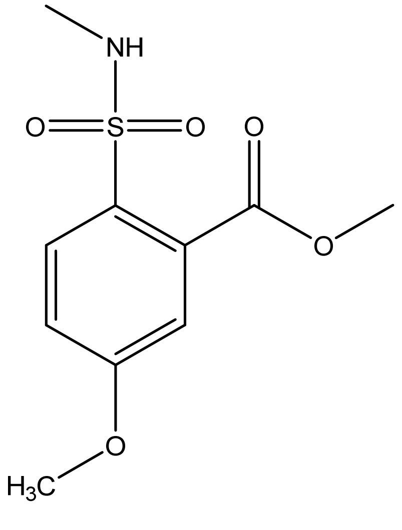

Prepared by treating **26mi** (0.6475 g, 2.5 mmol) in THF (10 ml) with LDA (1 M, 2 eq, 5 ml) in the manner described in illustrative procedure 7. The crude product was obtained as pale yellow semi solid (0.6086 g, 94%); the components were then

separated by column chromatography on silica eluting with petroleum ether (40 – 60 °C fraction) and ethyl acetate (2:1). The desired product which obtained as white needles (0.356 g, 55%), 86-87 °C.

HRMS (ESI) *m/z* calcd for $C_{10}H_{14}O_5NS^+$: 260.05872 [M+H]$^+$; found: 260.05756.

27mii*

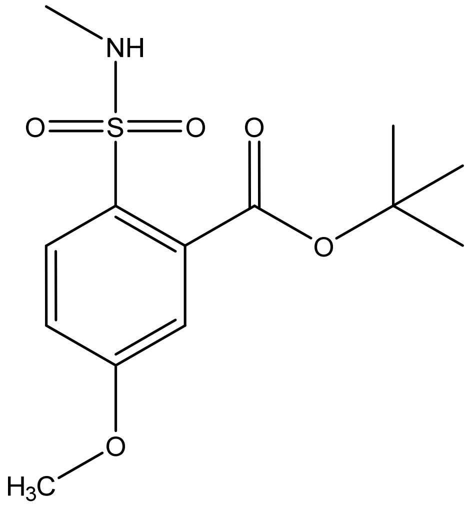

Prepared by treating **26mii** (0.7525 g, 2.5 mmol) in THF (10 ml) with LDA (1 M, 2 eq, 5 ml) in the manner described in illustrative procedure 7. The crude product was obtained as white solid (0.6697 g, 89%). Recrystallisation from ethanol/water gave white needles (0.52 g, 75%).

HRMS (ESI) *m/z* calcd for $C_{13}H_{20}O_5NS^+$: 302.10567 [M+H]$^+$; found: 302.10626.

27miii*

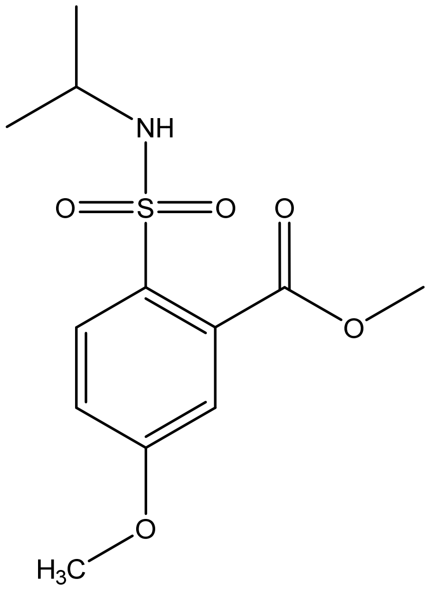

Prepared by treating **26miii** (0.7175 g, 2.5 mmol) in THF (10 ml) with LDA (1 M, 2 eq, 5 ml) in the manner described in illustrative procedure 7. The crude product was obtained as white solid (0.617 g, 86%). Recrystallisation from ethanol/water gave white iridescent leaflets (0.4519 g, 63%).

HRMS (ESI) *m/z* calcd for $C_{12}H_{18}O_5NS^+$: 288.09002 $[M+H]^+$; found: 288.08932.

27miv*

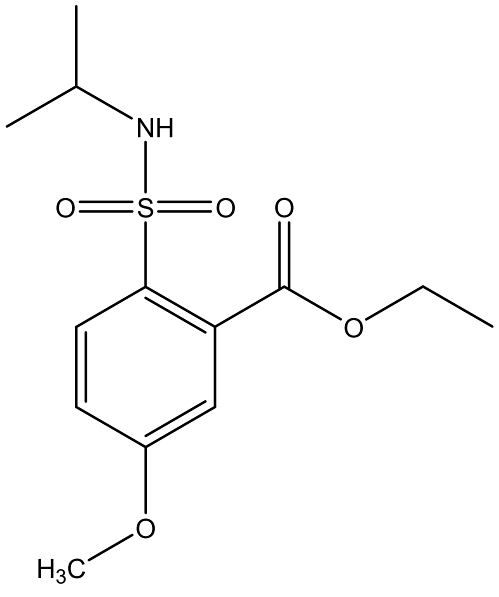

Prepared by treating **26miv** (0.752 g, 2.5 mmol) in THF (10 ml) with LDA (1 M, 2 eq, 5 ml) in the manner described in illustrative procedure 7. The crude product was obtained as white solid (0.677 g, 90%). Recrystallisation from ethanol/water gave white needles (0.631 g, 84%), mp 56-7 °C.

HRMS (ESI) *m/z* calcd for $C_{13}H_{20}O_5NS^+$: 302.10567 $[M+H]^+$; found: 302.10568.

27mv*

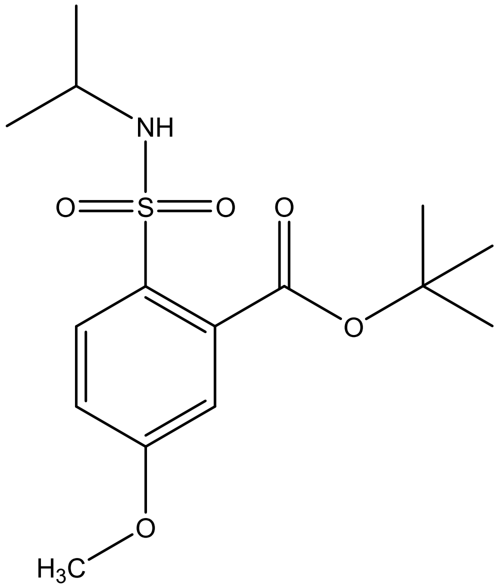

Prepared by treating **26mv** (0.8225 g, 2.5 mmol) in THF (10 ml) with LDA (1 M, 2 eq, 5 ml) in the manner described in illustrative procedure 7. The crude product was pale yellow solid (0.764 g, 93%). Recrystallisation from ethanol/water gave off white needles (0.69 g, 85%), mp 56-7 °C.

HRMS (ESI) *m/z* calcd for $C_{15}H_{24}O_5NS^+$: 330.13697 $[M+H]^+$; found: 330.13687.

27ni*

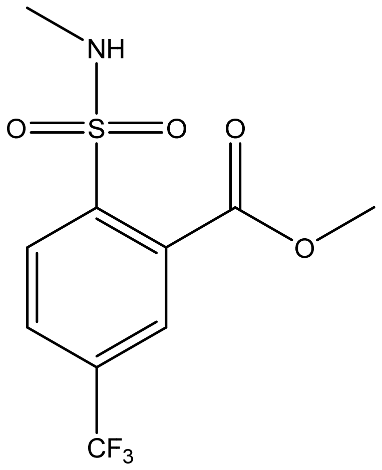

Prepared by treating **26ni** (0.679 g, 2.5 mmol) in THF (10 ml) with LDA (1 M, 2 eq, 5 ml) in the manner described in illustrative procedure 7. The crude product was obtained as orange viscous oil (0.49 g, 79%); the components were then separated by column chromatography on silica eluting with petroleum ether (40 – 60 °C fraction) and ethyl acetate (2:1). The desired product which obtained as a white needles (0.33 g, 54%).

27nii*

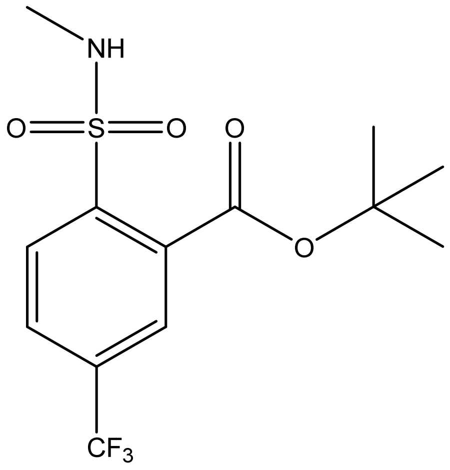

Prepared by treating **26nii** (0.679 g, 2.5 mmol) in THF (10 ml) with LDA (1 M, 2 eq, 5 ml) in the manner described in illustrative procedure 7. The crude product was

obtained as white solid (0.49 g, 79%). Recrystallisation from ethanol/water gave white iridescent leaflets (0.584 g, 85%), mp 66-7 °C.

HRMS (ESI) *m/z* calcd for $C_{13}H_{17}O_4NSF_3^+$: 340.08249 [M+H]$^+$; found: 340.08243.

27niii*

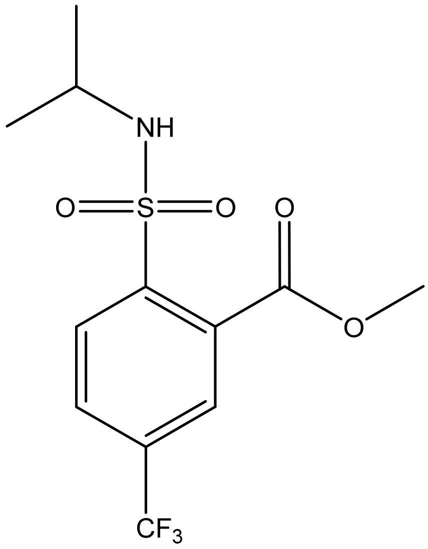

Prepared by treating **26nii** (0.8125 g, 2.5 mmol) in THF (10 ml) with LDA (1 M, 2 eq, 5 ml) in the manner described in illustrative procedure 7. The crude product was obtained as white solid (0.755 g, 93%). Recrystallisation from ethanol/water gave white needles (0.6175 g, 76%).

HRMS (ESI) *m/z* calcd for $C_{12}H_{15}O_4NSF_3^+$: 326.06684 [M+H]$^+$; found: 326.06668.

27niv*

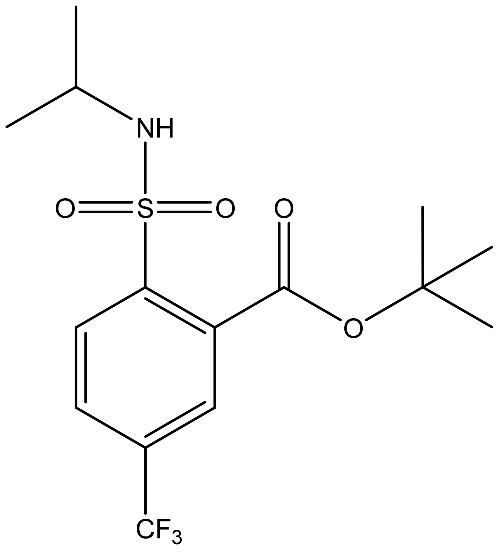

Prepared by treating **26niv** (0.9175 g, 2.5 mmol) in THF (10 ml) with LDA (1 M, 2 eq, 5 ml) in the manner described in illustrative procedure 7. The crude product was obtained as white solid (0.8165 g, 89%). Recrystallisation from ethanol/water gave white iridescent leaflets (0.762 g, 83%), mp 66-7 °C.

HRMS (ESI) *m/z* calcd for $C_{15}H_{21}O_4NSF_3^+$: 368.11379 $[M+H]^+$; found: 368.11383.

27oi*

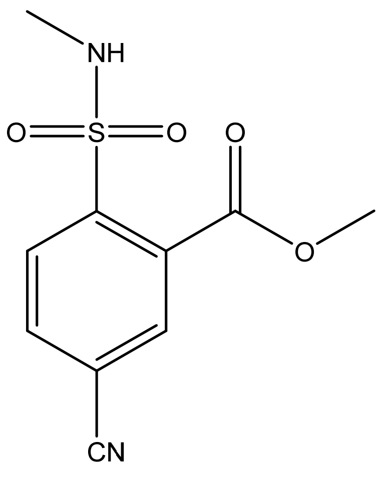

Prepared by treating **26oi** (0.635 g, 2.5 mmol) in THF (10 ml) with LDA (1 M, 2 eq, 5 ml) in the manner described in illustrative procedure 7. The crude product was obtained as yellow oil (0.44 g, 70%); the components were then separated by column

chromatography on silica eluting with petroleum ether (40 – 60 °C fraction) and ethyl acetate (2:1). The desired product which obtained as white needles (0.309 g, 45%).

27oii*

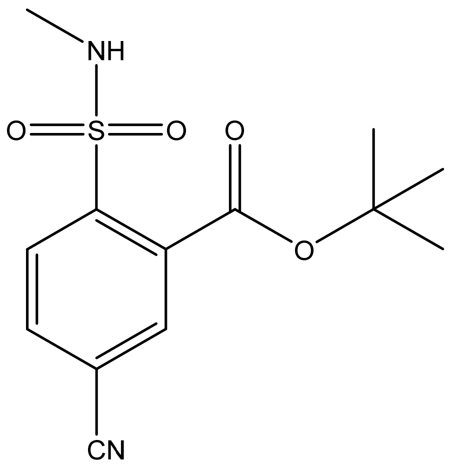

Prepared by treating **26oii** (0.74 g, 2.5 mmol) in THF (10 ml) with LDA (1 M, 2 eq, 5 ml) in the manner described in illustrative procedure 7. The crude product was obtained as off white solid (0.636 g, 86%).

HRMS (ESI) *m/z* calcd for $C_{13}H_{17}O_4N_2S^+$: 297.09035 $[M+H]^+$; found: 297.09058.

27oiii*

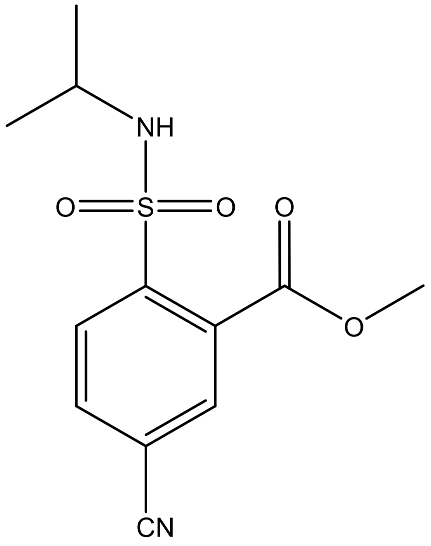

Prepared by treating **26oiii** (0.705 g, 2.5 mmol) in THF (10 ml) with LDA (1 M, 2 eq, 5 ml) in the manner described in illustrative procedure 7. The crude product was

obtained as yellow solid (0.528 g, 75%). Recrystallisation from ethanol/water gave white iridescent leaflets (0.409 g, 58%), mp 125-6 °C.

HRMS (ESI) *m/z* calcd for $C_{12}H_{15}O_4N_2S^+$: 283.07470 [M+H]$^+$; found: 283.07468.

27oiv*

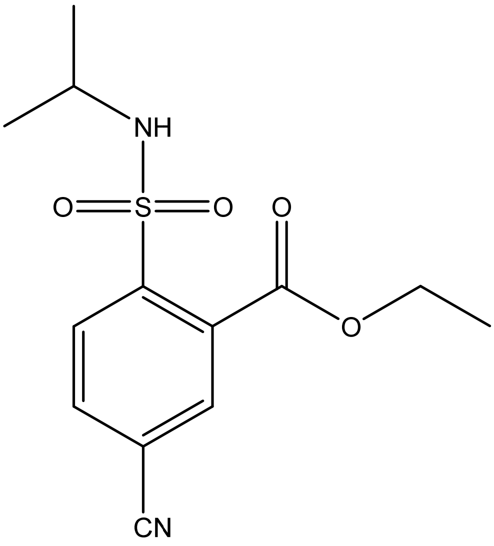

Prepared by treating **26oiv** (0.74 g, 2.5 mmol) in THF (10 ml) with LDA (1 M, 2 eq, 5 ml) in the manner described in illustrative procedure 7. The crude product was obtained as brown solid (0.584 g, 79%). Recrystallisation from ethanol/water gave beige solid (0.496 g, 67%).

27ov*

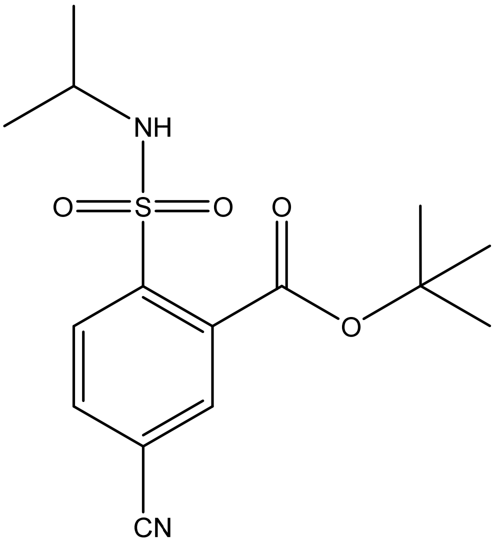

Prepared by treating **26ov** (0.81 g, 2.5 mmol) in THF (10 ml) with LDA (1 M, 2 eq, 5 ml) in the manner described in illustrative procedure 7. The crude product was obtained as white solid (0.713 g, 88%). Recrystallisation from ethanol/water gave white iridescent leaflets (0.616 g, 76%), mp 75-6 °C.

27pi*

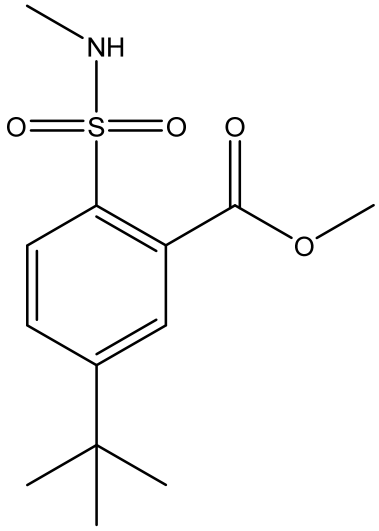

Prepared by treating **26pi** (0.7125 g, 2.5 mmol) in THF (10 ml) with LDA (1 M, 2 eq, 5 ml) in the manner described in illustrative procedure 7. The crude product was obtained as orange oil (0.69 g, 97%). Recrystallisation from ethanol/water gave white needles (0.55 g, 75%); mp 91-2 °C.

27pii*

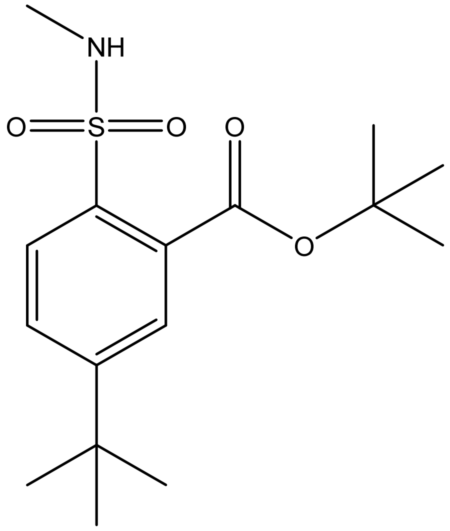

Prepared by treating **26pii** (0.7125 g, 2.5 mmol) in THF (10 ml) with LDA (1 M, 2 eq, 5 ml) in the manner described in illustrative procedure 7. The crude product was obtained as orange oil (0.68 g, 95%). Recrystallisation from ethanol/water gave white rhombs (0.65 g, 90%).

HRMS (ESI) *m/z* calcd for $C_{16}H_{26}O_4NS^+$: 328.15771 $[M+H]^+$; found: 328.15808.

27piii*

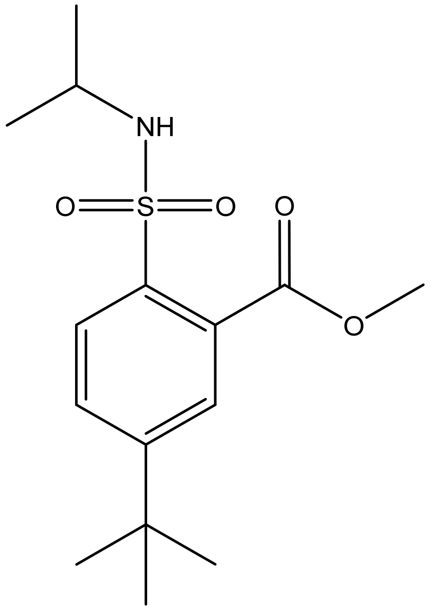

Prepared by treating **26piii** (0.782 g, 2.5 mmol) in THF (10 ml) with LDA (1 M, 2 eq, 5 ml) in the manner described in illustrative procedure 7. The crude product was obtained as pale orange solid (0.719 g, 92%); the components were then separated by column chromatography on silica eluting with petroleum ether (40 – 60 °C fraction) and ethyl acetate (2:1). The desired product which obtained as a white needles (0.571 g, 73%).

HRMS (ESI) *m/z* calcd for $C_{15}H_{24}O_4NS^+$: 314.14206 $[M+H]^+$; found: 314.14230.

27piv*

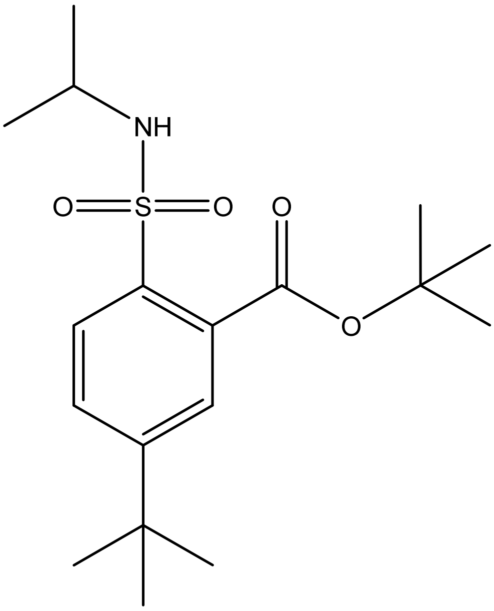

Prepared by treating **26piv** (0.887 g, 2.5 mmol) in THF (10 ml) with LDA (1 M, 2 eq, 5 ml) in the manner described in illustrative procedure 7. The crude product was obtained as white solid (0.860 g, 97%). Recrystallisation from ethanol/water gave white iridescent leaflets (0.754 g, 85%); mp 99-101 °C

HRMS (ESI) *m/z* calcd for $C_{18}H_{30}O_4NS^+$: 356.18901 $[M+H]^+$; found: 356.18903.

5 Saccharins

28a

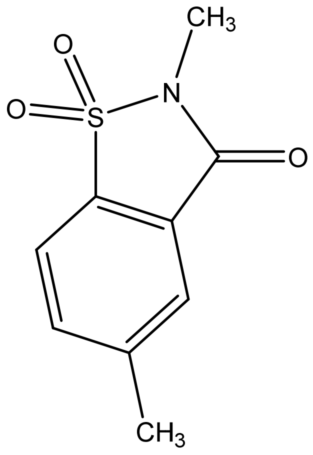

White crystalline solid (mp 100-101 C), isolated by chromatography as the slower running component from the mixture obtained in rearrangement of **26ai**. Recrystallisation from ethanol gave white crystals, mp 126-7 °C, lit 108-110 °C.

HRMS (ESI) *m/z* calcd for $C_9H_{10}O_3NS^+$: 212.03759 $[M+H]^+$; found: 212.03763.

28b

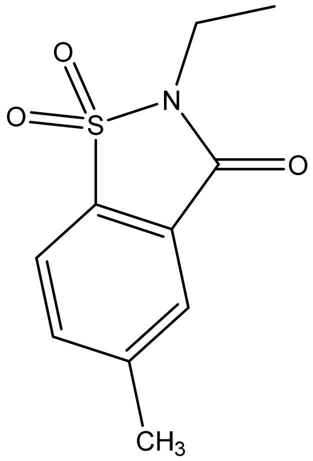

White solid isolated by chromatography as the slower running component from the mixture obtained in rearrangement of **26bi**. Recrystallisation from ethanol gave white crystals white iridescent leaflets (mp 66-67 °C) (lit 86-88 °C).

28c

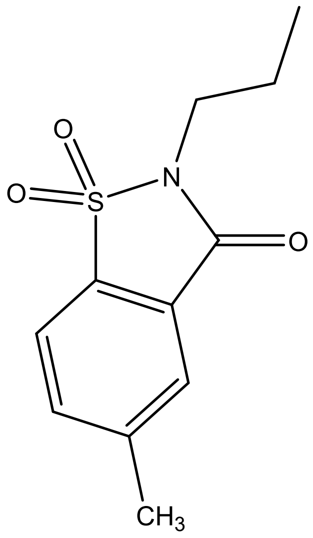

Off white crystalline solid isolated by chromatography as the slower running component from the mixture obtained in rearrangement of **26ci**. Recrystallisation from ethanol gave white crystals white rhombs (mp 55-6 °C).

28f

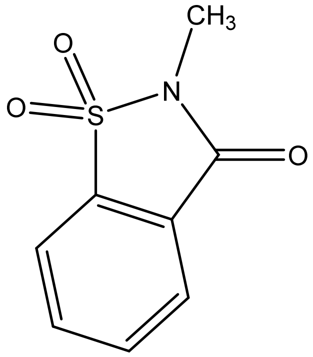

White rhombs (mp 130-131 °C), isolated by chromatography as the slower running component from the mixture obtained in rearrangement of **26fi;** mp 128-129 °C, lit mp 132-3 °C.
